# Antiangiogenic Activity of Flavonoids: A Systematic Review and Meta-Analysis

**DOI:** 10.3390/molecules25204712

**Published:** 2020-10-14

**Authors:** Mai Khater, Francesca Greco, Helen M. I. Osborn

**Affiliations:** 1 School of Pharmacy, University of Reading, Whiteknights, Reading RG6 6AD, UK; m.a.a.khater@pgr.reading.ac.uk (M.K.); f.greco@reading.ac.uk (F.G.); 2 Therapeutic Chemistry Department, Pharmaceutical & Drug Industries Research Division, National Research Centre, Cairo 12622, Egypt

**Keywords:** flavonoids, angiogenesis, inflammation, cancer, in-vivo angiogenesis, CAM assay, SAR

## Abstract

An imbalance of angiogenesis contributes to many pathologies such as cancer, arthritis and retinopathy, hence molecules that can modulate angiogenesis are of considerable therapeutic importance. Despite many reports on the promising antiangiogenic properties of naturally occurring flavonoids, no flavonoids have progressed to the clinic for this application. This systematic review and meta-analysis therefore evaluates the antiangiogenic activities of a wide range of flavonoids and is presented in two sections. The first part of the study (Systematic overview) included 402 articles identified by searching articles published before May 2020 using ScienceDirect, PubMed and Web of Science databases. From this initial search, different classes of flavonoids with antiangiogenic activities, related pathologies and use of in vitro and/or in/ex vivo angiogenesis assays were identified. In the second part (Meta-analysis), 25 studies concerning the antiangiogenic evaluation of flavonoids using the in vivo chick chorioallantoic membrane (CAM) assay were included, following a targeted search on articles published prior to June 2020. Meta-analysis of 15 out of the 25 eligible studies showed concentration dependent antiangiogenic activity of six compared subclasses of flavonoids with isoflavones, flavonols and flavones being the most active (64 to 80% reduction of blood vessels at 100 µM). Furthermore, the key structural features required for the antiangiogenic activity of flavonoids were derived from the pooled data in a structure activity relationship (SAR) study. All in all, flavonoids are promising candidates for the development of antiangiogenic agents, however further investigations are needed to determine the key structural features responsible for their activity.

## 1. Introduction

Angiogenesis is the process of forming new blood vessels. Physiologically, angiogenesis is pivotal for tissue growth and regeneration [1] which is beneficial for many processes including embryogenesis and wound healing. Regulation of angiogenesis is complex and is maintained by the balance between endogenous stimulators (e.g., vascular endothelial growth factor (VEGF), platelet derived growth factors (PDGFs) and hypoxia-inducible factors (HIFs)), and inhibitors (e.g., angiostatin and endostatin). Other body conditions also contribute to the regulation of angiogenesis under physiological conditions. For example, certain metabolic demands such as the need for more oxygen can induce VEGF secretion and angiogenesis in heart and brain tissues [2,3]. Since angiogenesis affects many organs and tissues in the body, an imbalance in its regulation has been associated with different pathologies [4]. For instance, cancer, rheumatoid arthritis and diabetic retinopathy feature an upregulation of proangiogenic factors [5]. Conversely, if antiangiogenic factors were upregulated, several cardiovascular diseases are more likely to happen [6]. The use of drugs like Bevacizumab (Avastin^®^, Genentech) and Aflibercept (Eylea^®^, Regeneron) for the treatment of cancer and ocular diseases, emphasizes the imperative medicinal applications of antiangiogenic agents [7,8].

Flavonoids are widely distributed in fruits, vegetables and nuts. They are one of the most important chemical classes of natural compounds showing various pharmacological profiles that include anticancer [9,10,11], anti-inflammatory [12], cardioprotective [13] and neuroprotective activities [14].

The antiangiogenic activity of flavonoids has been extensively studied over the last two decades. Several studies document the ability of flavonoids to inhibit the proliferation and migration of endothelial cells by interfering with key angiogenesis signaling cascades such as the mitogen activated protein kinase (MAPK) and phosphoinositide 3-kinase (PI3K) pathways. Nevertheless, they can inhibit the expression of major proangiogenic factors such as VEGF and matrix metalloproteinases (MMPs) [2,7,15].

Researchers rely on different in vitro and in/ex vivo assays to quantitatively assess the effects of chemical compounds on angiogenesis [16,17]. Each of these assays can probe one or more of the different steps involved in the angiogenesis process such as cell proliferation, migration and tubulogenesis.

Despite considerable research concerning the antiangiogenic activities of flavonoids, to date they have neither progressed to the market nor clinical trials for that purpose. Therefore, the aim of this review is to systematically assess the antiangiogenic activities of flavonoids to provide greater insight into their potential as therapeutic agents. This study is comprised of two parts: Section 1 provides a systematic overview of the classes of flavonoids that have been investigated for their antiangiogenic activities, along with a summary of the different in vitro and/or in/ex vivo angiogenesis assays that have been used; Section 2 is a meta-analysis study of a quantitatively comparative subset of data, based on the in vivo chick chorioallantoic membrane (CAM) assay, to statistically evaluate the antiangiogenic effects of flavonoids.

## 2. Results

### 2.1. Section 1: Systematic Overview

#### 2.1.1. Search Results

**Figure 1:**
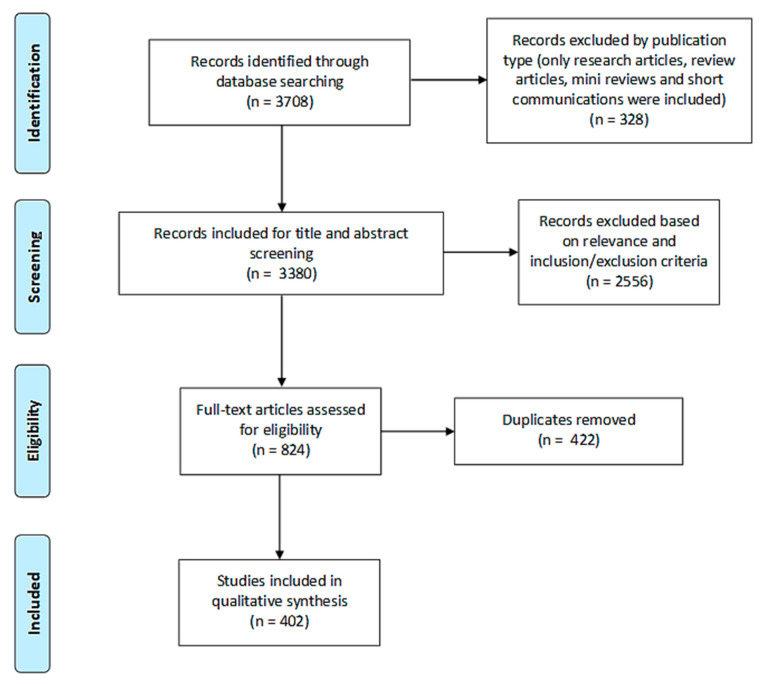
PRISMA flow diagram of study search and selection process of Section 1.

For Section 1 of the study, 3708 records were initially identified in three electronic databases (1555 from ScienceDirect, 1984 from PubMed and 169 from Web of Science). Search results were then limited to research articles, review articles, short communications and systematic reviews and the remaining 3380 articles were subjected to title and abstract screening. 2556 records were found to be irrelevant of the subject in focus or did not fulfill the inclusion criteria. After the removal of duplicates (422), 402 articles were finally included in the qualitative analysis for Section 1 of this study (Figure 1).

#### 2.1.2. Study Characteristics

**Figure 2:**
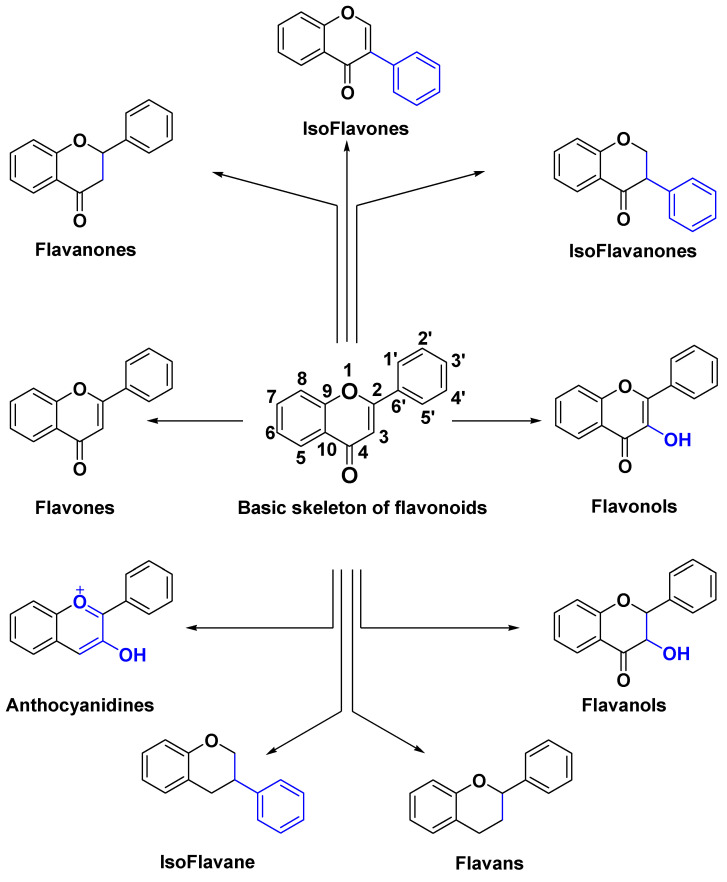
Chemical structures of classes of flavonoids.

The pool of studies included was classified with respect to: (a) flavonoid class (Figure 2), (b) flavonoid name, (c) disease, (d) in vitro test and (e) in/ex vivo tests. Characteristics of the included studies are summarized in Table 1 (flavonols are used as a representative example in Table 1 and Table S1 contains similar data for all classes of flavonoids). A total of 402 research and review articles were considered. All of the included articles reported angiogenesis related in vitro and/or in/ex vivo assays for different classes of flavonoids.

#### 2.1.3. Data Analysis

**Figure 3:**
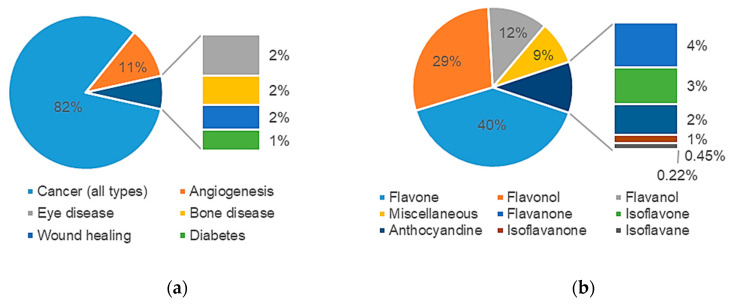
Profiling of papers retrieved in Section 1 with respect to: (**a**) pathology type; (**b**) chemical class of flavonoid.

The majority of articles (332, 82%) focused on the implications of angiogenesis on cancer growth and metastasis. 7% of the articles studied antiangiogenic effects of flavonoids on other diseases such as diabetes, bone and eye diseases, whilst 11% focused on the antiangiogenic activity of flavonoids without application to a specific pathology (Figure 3a). A profiling of the studies retrieved with respect to chemical class of flavonoids is shown in Figure 3b.

**Figure 4:**
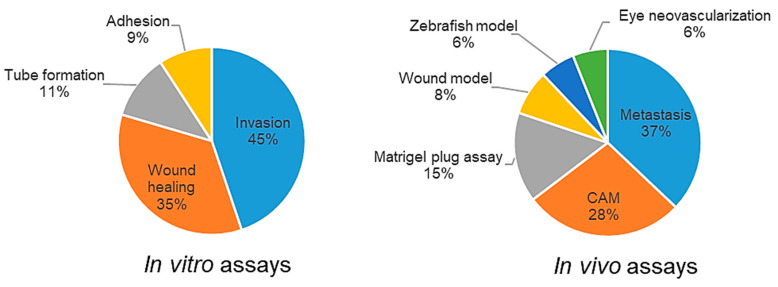
Types of assays used for in vitro and in vivo antiangiogenic evaluation of flavonoids.

Figure 4 summarizes the types of in vitro and in vivo assays that were utilized in the studies. From a pool of 342 research articles included in this study, 152 articles (44%) reported a combination of in vitro and in/ex vivo assays in their studies. The percentage of research articles that depended only on in/ex vivo tests to evaluate antiangiogenic activity of flavonoids were comparatively low compared to those conducting only in vitro assays (3% vs. 53%, respectively).

### 2.2. Section 2: Meta-Analysis

#### 2.2.1. Search Results

**Figure 5:**
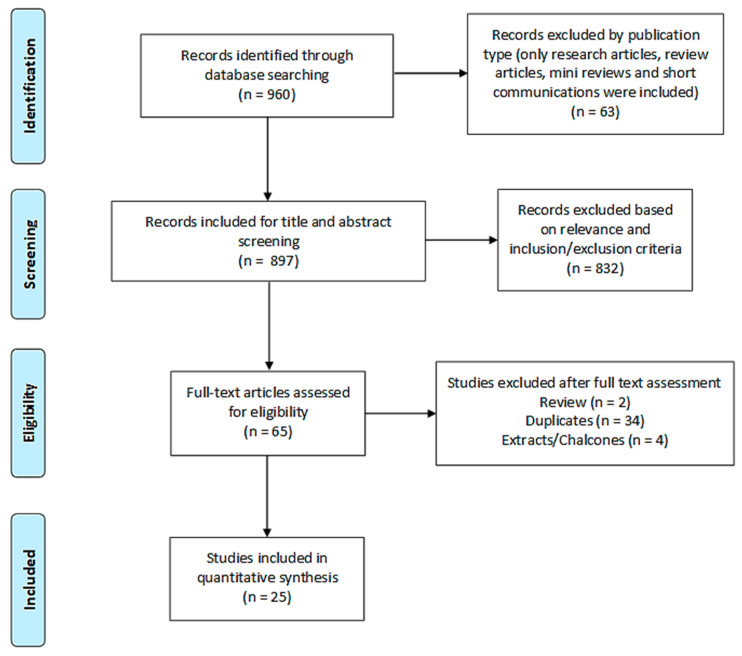
PRISMA flow diagram of study search and selection process of Section 2.

The second subset search, which is the basis of the meta-analysis forming Section 2 of this study, followed the same general methodology as detailed in the initial overview. 960 records were identified from four electronic databases (381 from ScienceDirect, 496 from PubMed, 65 from Web of Science and 18 from Google Scholar). 25 research articles were finally included in the quantitative analysis after the sequential steps of screening and sifting, as shown in Figure 5.

#### 2.2.2. Study Characteristics

The main study characteristics of the research articles included in Section 2 for the meta-analysis are summarized in Table 2 by study name.

#### 2.2.3. Meta-Analysis (Antiangiogenic Effect of Flavonoids on CAMs)

25 studies reporting the CAM assay for the in vivo evaluation of flavonoids were eligible for the meta-analysis. The number of blood vessels relative to the control was used as the outcome measure, the lower the ratio the higher the antiangiogenic activity. The studies were grouped into 3 sub-sets based on the controls used. In the first set (12 studies), the normal vasculature of the CAM was used as a control without any interventions that would induce angiogenesis. The second and third sets, however, tested the antiangiogenic activity of flavonoids on CAMs with abnormal angiogenesis using either proangiogenic factors for set 2 (4 studies) or cancer cell lines for set 3 (9 studies). 10 studies [18,95,100,132,137,139,140,141,145,149] out of the 25 eligible studies were not included in any of the conducted meta-analyses as they failed to report the required data outcomes or did not fit under any particular subgroup.

Set 1: Antiangiogenic effect of flavonoids under normal conditions

**Figure 6:**
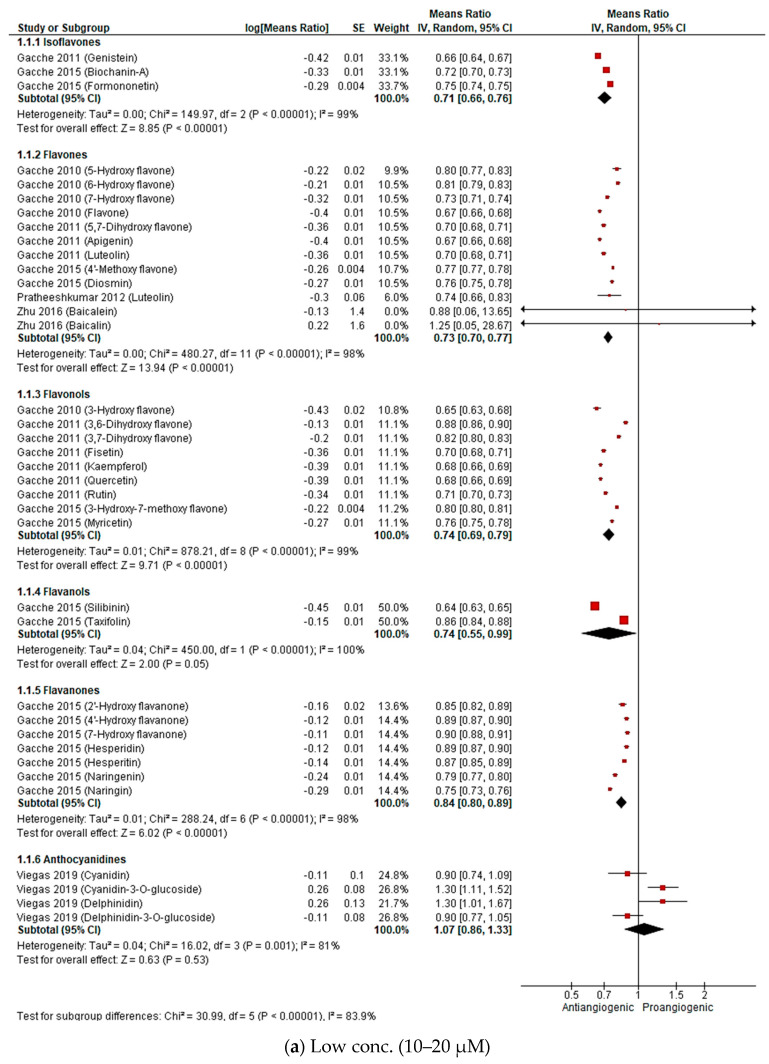

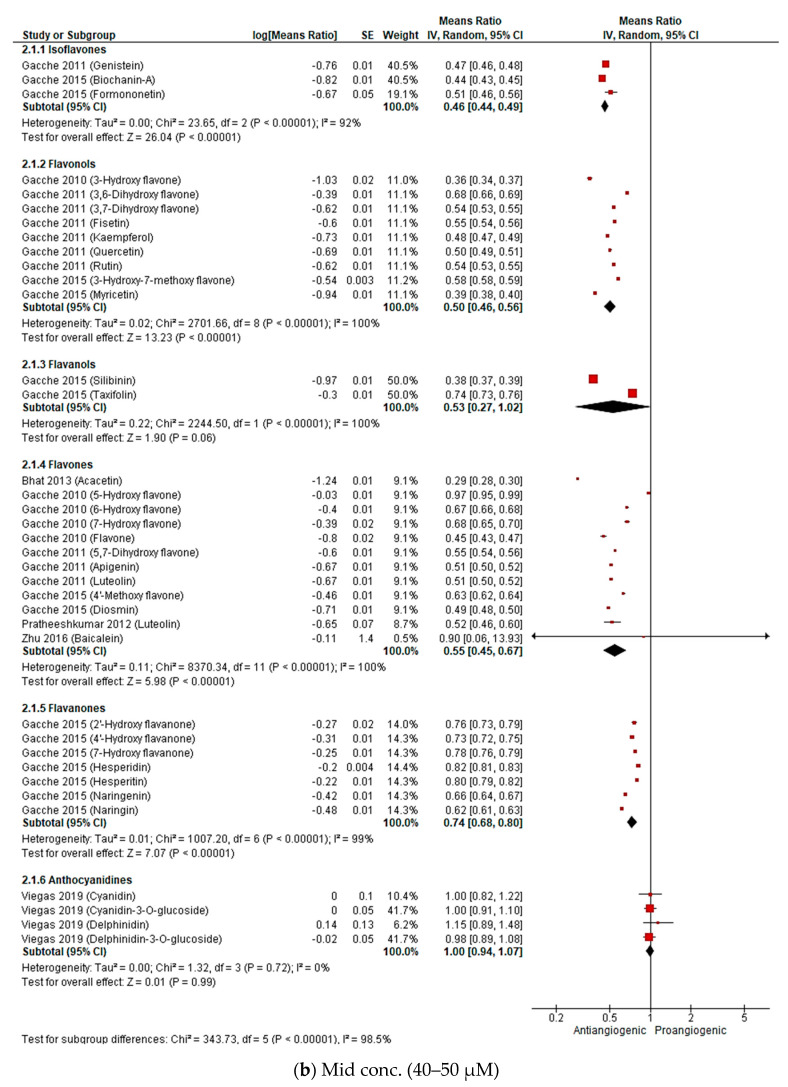

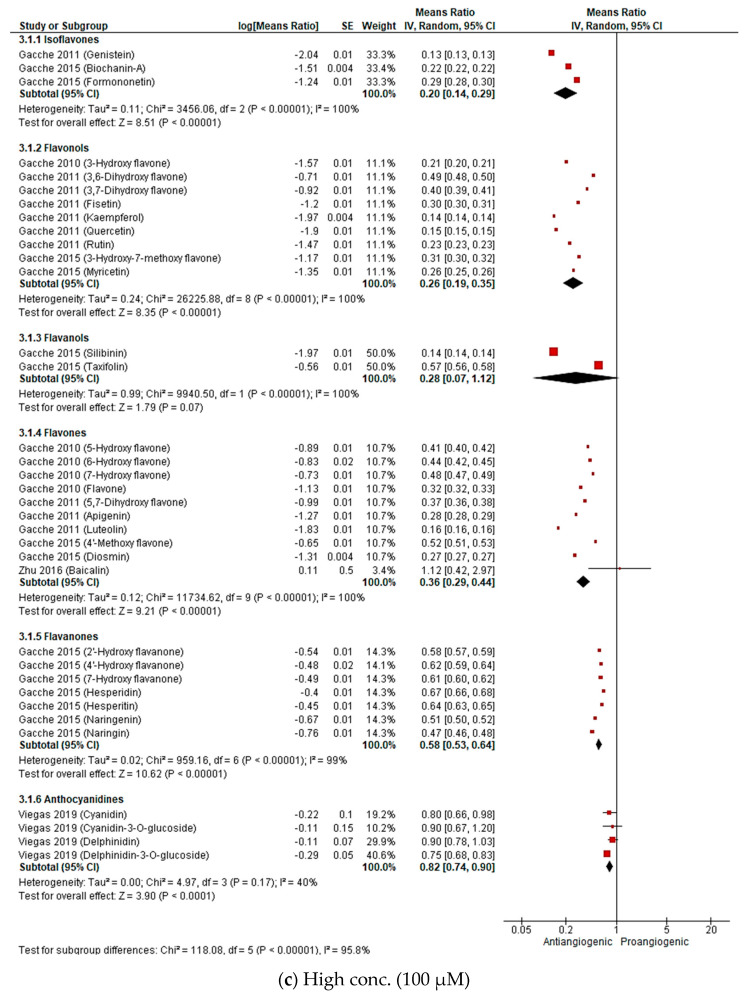
Forest plots of means ratio and 95% confidence interval (CI) of number of blood vessels relative to control at 3 concentration ranges as calculated by inverse variance (IV) method: (**a**) low (10–20 µM); (**b**) medium (40–50 µM); (**c**) high (100 µM).

To ensure consistency in our comparison, for the meta-analysis of set 1, the concentrations were grouped into three ranges i.e., low (10–20 µM), medium (40–50 µM) and high (100 µM). Flavonoids were sub grouped based on their chemical class as shown in Figure 6. Pooled results indicate that all subclasses, except for anthocyanidines, demonstrate concentration dependent antiangiogenic activity expressed as a reduction in the number of blood vessels in a CAM. For the flavonols subgroup, for instance, the overall means ratios (summary estimates of antiangiogenic activity of a subgroup of flavonoids relative to control) were 0.74 (95%CI: 0.69, 0.79; *p*-value < 0.00001), 0.50 (95%CI: 0.46, 0.56; *p*-value < 0.00001) and 0.26 (95%CI: 0.19, 0.35; *p*-value < 0.00001) for the low, mid and high concentrations, respectively. On the other hand, the anthocyanidines subgroup exhibited only a minor overall reduction of 18% at the highest concentration and a slightly proangiogenic effect (overall means ratio: 1.07; 95%CI: 0.86, 1.33; *p*-value: 0.53) at 20 µM.

**Figure 7:**
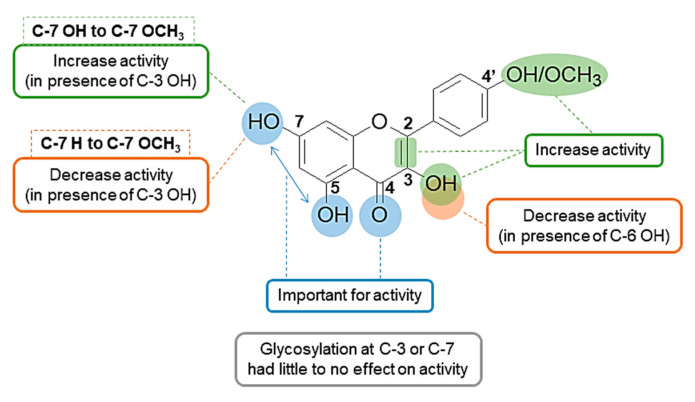
Summary of antiangiogenic SAR of flavonoids.

In addition to the forest plot analysis that gives a general idea about the overall in vivo antiangiogenic activity of flavonoids and identifies trends of activity among the different subclasses, some structure activity relationship (SAR) conclusions were drawn from the pooled results (Figure 7).

First, there was no correlation between the number of hydroxyl groups and antiangiogenic activity. However, the position of the hydroxyl groups appeared to be of importance as most of the highly active flavonoids had hydroxyl groups at positions 3, 5 and 7 and/or 4′ (e.g., as demonstrated for 3-OH flavone, acacetin, biochanin A, apigenin, silibinin and kaempferol). The 7-OH group can be considered to be of greatest importance for activity since 7-OH flavone showed higher activity in the low and medium concentrations compared to the 5-OH analogue. Absence of the 3-OH group caused up to a 14% decrease in activity at the 50 and 100 µM concentrations, as demonstrated, for example, for 3-OH flavone vs. flavone, kaempferol vs. apigenin and 3,7-diOH flavone vs. 7-OH flavone. This was also true for quercetin vs. luteolin but with only a trivial drop of activity of 1 to 2%. However, this was not the case for 3,6-diOH flavone vs. 6-OH flavone where removal of the 3-OH group slightly increased the activity by 1 to 5% at the mid and high concentrations.

Secondly, unsaturation of the C2 and C3 bond is a common feature of most of the highly active flavonoids and is important for activity. 7-OH flavone and 7-OH flavanone are two good examples that exemplify this, as demonstrated by a reduction of the number of vessels: 27%, 32% and 52% for 7-OH flavone and 10%, 22% and 39% for 7-OH flavanone at 10 µM, 50 µM, and 100 µM, respectively.

Third, there are examples of where the presence of a methoxy group at position 4′ increases activity (e.g., biochanin A, diosmin and formononetin). However, the presence of a methoxy group at C7 caused a decrease in the activity when compared to the unsubstituted analogue (ie for the 3-OH flavone vs. 3-OH-7-OCH_3_ flavone, reduction of number of vessels: 35% and 20% at 10 µM, 64% and 42% at 50 µM, 79% and 69% at 100 µM, respectively). On the other hand, conversion of the 7-OH group in 3,7-diOH flavone to a 7-OCH_3_ group in 3-OH-7-OCH_3_ flavone slightly improved the activity (reduction of number of vessels) from 18% to 20% at 10 µM and from 63% to 69% at 100 µM, respectively. Finally, glycosylation at positions 3 or 7 showed neither a pronounced nor a consistent effect on the antiangiogenic activity of flavonoids. While a decrease in activity was observed with quercetin vs. rutin, hesperitin vs. hesperidin and cyanidin vs. cyanidin-3-*O*-glucoside, an increase was observed in the cases of naringin vs. naringenin and delphinidin vs. delphinidin-3-*O*-glucoside.

Set 2: Antiangiogenic effect of flavonoids under inflammatory conditions

**Figure 8:**
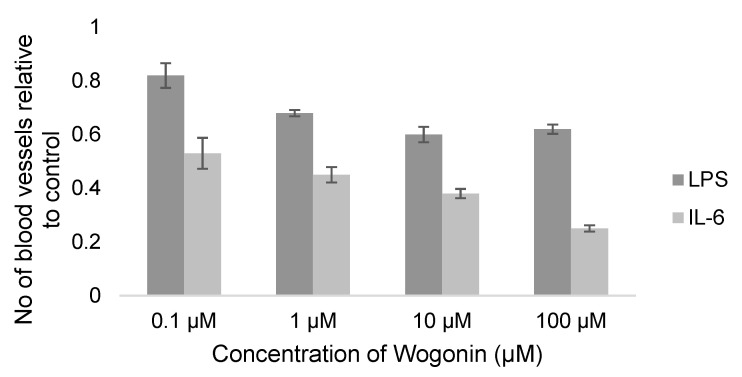
Reported antiangiogenic effect of wogonin on LPS and IL-6 induced angiogenesis ± SEM.

Lin et al. evaluated the antiangiogenic activity of the flavone wogonin on LPS (the main component of gram negative bacterial membrane) and IL-6 induced angiogenesis in two reports [130,147]. The documented reduction in the number of CAM blood vessels by wogonin was shown to be dose dependent in both cases but more prominent in the case of IL-6 induced angiogenesis (75% as opposed to 38% in the case of LPS induced angiogenesis at 100 µM) (Figure 8). The authors also probed the possible mechanisms of wogonin’s inhibition of this inflammation-induced angiogenesis through different in vitro techniques such as western blotting and polymerase chain reaction (PCR) in which both LPS and IL-6 resulted in an upregulation of the IL-6/IL-6R pathway [130,147]. Although wogonin attenuated the IL-6/IL-6R pathway and levels of VEGF in both cases, it exhibited different expression of downstream vascular endothelial growth factor receptors (VEGFRs). Only VEGFR2 expression was downregulated with wogonin LPS-induced angiogenesis inhibition as opposed to VEGFR1 downregulation with IL-6 induced angiogenesis inhibition. This data needs further investigation in order to understand why these two similar mechanisms lead to the downregulation of two different downstream receptors (VGFR2 and VEGFR1) and to address the impact of this on the antiangiogenic potency. Inhibition of LPS-induced angiogenesis was also reported for wogonoside, which is the 7-glucuronic acid of wogonin, by Chen et al. [139] 150 ng/CAM of wogonoside reduced neo-vascularization of CAMs by 43%. Additionally, wogonoside downregulated mammalian toll-like receptor (TLR4), extracellular signal-regulated kinase (ERK1/2) and p38MAPK in a western blotting assay [139].

Set 3: Antiangiogenic effect of flavonoids under tumor conditions.

**Figure 9:**
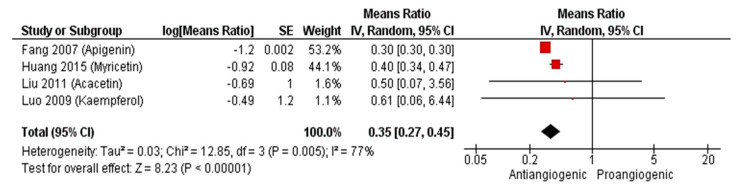
Forest plot of means ratio and 95% confidence interval (CI) of number of blood vessels relative to control of flavonoids on OVCAR-3 cell lines.

**Figure 10:**
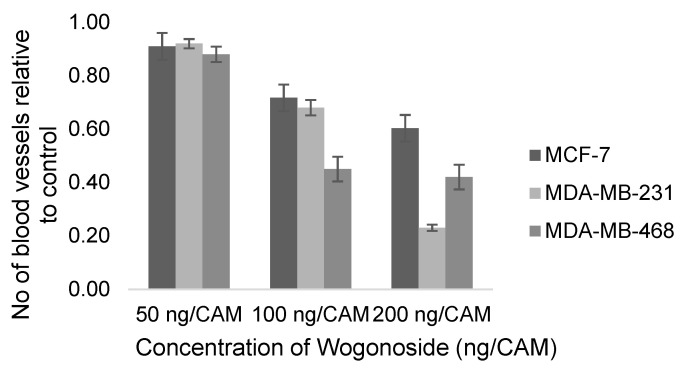
Reported antiangiogenic effect of wogonoside on breast cancer cell lines; MCF-7, MDA-MB-231 and MDA-MB-468 ± SEM.

Since angiogenesis plays a vital role in tumor growth and metastasis, several studies have focused on the antiangiogenic evaluation of promising cytotoxic agents. Figure 9 shows the estimated antiangiogenic effect of the 4 flavonoids apigenin, myricetin, acacetin and keampferol on the ovarian cancer cell line (OVCAR-3) at 10–20 µM. The reduction in the number of CAM blood vessels ranged from 30 to 60% with an overall summary outcome of 0.35 (95%CI: 0.27, 0.45; *p*-value < 0.00001). HIFα and VEGF were significantly downregulated, as evidenced by immunoblotting analysis of CAM OVCAR-3 tissues that were treated with apigenin or acacetin [131,144]. The antiangiogenic activity of the flavone wogonoside was evaluated on the estrogen receptor positive (MCF-7) and two triple negative breast (MDA-MB-231 and MDA-MB-468) cancer cell lines by Huang et al. [138,142]. At 50 ng/CAM, wogonoside’s effect on the 3 cell lines was not prominent (Figure 10). However, a 55% reduction of the number of blood vessels was observed at 100 ng/CAM for the MDA-MB-468 cell line. A two-fold increase in the concentration of wogonoside to 200 ng/CAM did not, however, result in an increased antiangiogenic effect on the same cell line. On the other hand, reduction of the neo-vascularization for the MDA-MB-231 cell line increased from 32% to 77% upon increasing the concentration from 100 to 200 ng/CAM. Huang et al. demonstrated the ability of wogonoside to target the Hedgehog signaling pathway, which is upregulated in triple negative breast cancer, in MDA-MB-231 and MDA-MB-468 cell lines [138]. Expression of the Hedgehog downstream transmembrane protein smoothened (SMO) and glioma-associated oncogene homolog protein (Gli), is significantly increased in triple negative breast cancer [150] leading to an elevation in VEGF levels [151]. According to Huang and his colleagues, wogonoside promoted SMO degradation and inhibited Gli1 activity as well as expression of VEGF [138].

#### 2.2.4. Sensitivity Analysis

The high heterogeneity (*I*^2^ > 80%) observed for all subgroups in the generated forest plots, except for the anthocyanidines subgroup at the mid and high concentrations analyses (*I*^2^ *=* 0% and 40%, respectively), was expected given that each class included different flavonoid molecules. In that context, a sensitivity analysis was conducted by a leave-one-out strategy to assess the robustness of the results and determine the contribution of each flavonoid to heterogeneity. Overall, the results showed good robustness and the overall summary estimates did not show significant changes upon the systematic removal of individual studies (Tables S2–S4). This was the case in all subgroups with the exception of the flavanol subgroup which showed some difference in the overall summary at all concentrations. At the 40–50 µM range for instance, the overall pooled means ratio changed from 0.53 (95%CI: 0.27, 1.02, *I*^2^ = 100%) to 0.74 (95%CI: 0.73, 0.76, NA) and 0.38 (95%CI: 0.37, 0.39, NA) upon removal of the Gacche 2015 (Silibinin) and Gacche 2015 (Taxifolin) flavonoids, respectively (Table S3). This indicates that data provided on the flavanols subgroup is not sufficient to draw meaningful conclusions. Likewise, heterogeneity (*I*^2^) of the subgroups totals did not show significant change, with very few exceptions, upon implementation of the leave-one-out strategy (Tables S2–S4). This might be due to the fact that most of the flavonoids in a single subgroup belong to the same study, consequently, there are no differences in their experimental designs. In that case heterogeneity is believed to be either of clinical or statistical origin.

## 3. Discussion

Flavonoids have been reported to modulate several angiogenic factors and cascades in either a proangiogenic or an antiangiogenic manner which is postulated to be dose dependent [2,148]. A good illustration of this dual effect is demonstrated by the flavone baicalin; low doses were reported to stimulate angiogenesis [152] whilst high doses showed an inhibitory effect [153]. Due to the emerging importance of the use of angiogenesis modulators in the treatment of various pathological conditions including cancer, diabetes, bone, eye, cardiovascular and neurological disorders, the identification of flavonoids altering angiogenesis has gained new significance [2,154]. To the best of our knowledge, no systematic reviews have been conducted to quantitatively assess the antiangiogenic effects of flavonoids, despite the potential of such a study to have a positive impact on the treatment of serious health issues like cancer and rheumatoid arthritis. Given the breadth of the literature related to the antiangiogenic effects of flavonoids, a systematic search of the literature was initially conducted in this research program to identify (a) the extent to which angiogenesis modulation effects had been proposed for flavonoids and (b) the most widely used in vitro and in/ex vivo assays to determine the antiangiogenic activities of flavonoids.

Various study designs have been used in the literature to report on the antiangiogenic activity of chemical compounds. There are a number of comprehensive reviews in the literature comparing the different available angiogenesis assay models [16,17,155,156]. Although in vitro studies are less expensive and quicker to perform than in vivo studies, the results do not always convert into the same effect, in vivo. In vitro assays usually focus on monitoring the individual steps of angiogenesis such as migration or proliferation of endothelial cells rather than the collective formation of new tube-like structures [16]. In vivo assays offer the considerable advantage of mimicking more closely the body’s physiological conditions which is particularly important in angiogenic studies due to the complex nature of the process. While in vivo angiogenesis assays can be more informative, they present some cost, time and experimental design limitations. Inflammation resulting from the trauma that is caused by some assays, for instance, can stimulate several proangiogenic factors which compromise the sensitivity and specificity of the results [17]. Hence, it is recommended that a combination of in vitro and in vivo assays is used to provide consistent and complementary results. In relation to this, 44% of the research articles included in the conducted preliminary search reported a combination of in vitro and in/ex vivo assays.

Herein, a meta-analysis study was carried out in order to quantitatively evaluate the antiangiogenic effects of flavonoids. Only articles implementing the CAM assay in their study design were included. This is because the CAM assay is currently the most widely used in vivo angiogenic assay and, as such, it allows a comparison across different flavonoid types and offers many advantages over other angiogenic assays [157,158,159]. For instance, it is fairly simple, inexpensive, suitable for large scale screening and also offers the important advantage of expressing almost all of the known angiogenic factors [17,156]. Set-up of the assay is briefly as follows: fertilized chicken eggs are incubated at 37 °C for 3 days, a small hole is made in the egg shell to remove some of the albumin in order to facilitate detachment of the CAM from the shell. Compounds under investigation are added to approximately 5 to 10 day old chicks on specific carriers, such as matrigel or sterile filter/plastic discs, through a small window cut in the egg shell. After 48 to 72 h, existing blood vessels or tubules can be visualized and evaluated by light or electron microscopy [17,156]. Nevertheless, the CAM test comes with certain limitations such as sensitivity to oxygen tension and difficulty of visualization of newly formed vessels due to the presence of pre-existing ones [157].

Meta-analysis of results of the antiangiogenic evaluation of flavonoids via the in vivo CAM assay showed increasing activities with increasing concentrations. The evaluated flavonoids also demonstrated antiangiogenic activities of varying potencies. In light of this, results were inspected to gain some insights on the SAR of antiangiogenic activity of flavonoids (Figure 7). Although SARs of chemical compounds change based on the sought pharmacological activity, there are some common structural features of flavonoids that are recognized as important for activity [160]. Combination of the C2=C3 double bond and a 4-C=O is favorable for the antiviral/bacterial [161], anticancer [162,163], cardioprotective [164], anti-inflammatory [165], and antioxidant [164] activities of flavonoids. This conjugation maintains the planarity of the molecule and helps with the electron delocalization between rings A and C which is important for interaction with several targets [160]. Similarly, the 5, 7 di-OH is important for many of the biological activities of flavonoids [164,166,167,168]. This can be explained by the fact that flavonoids exert different pharmacological activities that have mutual and/or overlapping mechanisms. For example, the antioxidant activity of flavonoids contributes to their anti-inflammatory activity and both contribute to their anticancer activity. Moreover, several targets in the body have structurally similar binding sites and this is a phenomenon that is partially responsible for drug promiscuity or polypharmacology (binding of a drug to multiple targets). This was, in fact, observed for binding of the flavonoid quercetin with phosphatidylinositol 4,5-bisphosphate 3-kinase (PI3KCG) and the serine/threonine proto oncogene, PIM1 kinase [169].

With respect to the antiangiogenic activity of flavonoids, limited SAR studies have been reported. Lam et al. tested the antiangiogenic activity of a number of polymethoxylated flavonoids in vitro and in vivo [170]. The authors concluded that methylation of C5, C6, C7 and/or C4′ OH groups increased the activity which is in agreement with Ravishankar et al. [93] who reported the in vitro antiangiogenic activity of a number of quercetin and luteolin derivatives. Our results also suggest that the presence of a 4′-OCH_3_ increases the antiangiogenic activity. Despite this, there were some discrepancies between the aforementioned SAR conclusions. In this SAR analysis we showed that the presence of a 3-OH group enhanced the antiangiogenic activity, which is in contrast to the report from Ravishankar et al. that noted that the same 3-OH caused a drop in the activity yet methylation of that OH increased the activity [93]. A study by Lam et al. reported that glycosylation at C7 dramatically decreased the activity [170] while our study showed such modification to cause a minor or no decrease in the activity and even a slight increase in some cases. These inconsistencies are likely to be a result of the different experimental methodologies and flavonoid concentrations used in each study. Additionally, the different evaluated flavonoids might exert their antiangiogenic activities by binding to different targets that require different structural features. This highlights the need for larger scale studies to more fully probe the antiangiogenic SAR of flavonoids taking in consideration the employed mechanisms of action.

Since the relation between inflammation and angiogenesis is well established and many flavonoids possess anti-inflammatory activities, several studies assessed the antiangiogenic effects of flavonoids on inflammation-induced angiogenesis. Inflammatory cells like T-lymphocytes and macrophages secrete cytokines that can control the survival, proliferation, activation and migration of endothelial cells [171,172]. Endothelial cells can additionally produce several cytokines and chemokines themselves [173]. Flavonoids such as baicalin, quercetin and kaempferol caused a reduction in both inflammatory and angiogenic markers in cultured macrophages and human umbilical vein endothelial cells (HUVECs) [174,175].

Bacterial infections also trigger angiogenesis through inflammatory pathways. In that context, binding of LPS to the TLR4 receptor located on the surface of endothelial cells leads to upregulation of ERK1/2 and p38MAPK pathways and increases production of pro-inflammatory cytokines like IL-6 [176,177]. Pro-inflammatory cytokines like IL-6 and tumor necrosis factor α (TNFα) can interact with VEGF expression and promote angiogenesis [178,179]. The flavone, wogonin, and its glucoside, wogonoside, showed promising antiangiogenic activity against LPS induced angiogenesis [130]. Wogonin also inhibited IL-6 induced angiogenesis in a concentration dependent manner where it was reported to downregulate VEGFR1 not VEGFR2 genetic expression [147]. While VEGR2 is the main receptor for VEGF and is downregulated by many flavonoids [91,180], VEGFR1‘s role in angiogenesis is still not fully understood and needs further investigation.

As mentioned earlier, cancer is one of the most serious pathologies related to angiogenesis. When cells grow malignantly beyond a certain size, they need more vascularization to receive oxygen and nutrients i.e., tumors depend on angiogenesis to grow above a certain limit, and to metastasize [181]. The tumor vasculature is characterized by an imbalance between pro and anti-angiogenic factors where several angiogenic stimulators like VEGF and HIF are overexpressed. The HIFs are major regulators of angiogenesis and orchestrate many of the steps involved [182]. Under physiological conditions, HIFs are released in response to low oxygen levels in the blood (hypoxia) and stimulate angiogenesis at various levels from endothelial cell proliferation to activating the transcription of angiogenic genes like VEGF and platelet derived growth factor (PDGF). During malignancy, HIF dependent angiogenesis is activated either in response to the predominant hypoxic environment or by the genetic transformations caused by cancer. Flavonoids can downregulate HIFα and VEGF in different cancer cell lines such as OVCAR-3, A2780, MCF-7 and PC-3 [42,70,95,131,144,145]. Many studies have also reported the ability of the flavonoids 3-hydroxy flavone, hesperidin, apigenin, fisetin and many others to reduce tumor size, capillary density and metastasis of different cancers, such as osteosarcoma, melanoma, lung and breast cancers, in xenograft mice [26,183,184,185,186,187].

Although this meta-analysis demonstrated the overall promising in vivo antiangiogenic activity of flavonoids whether in normal, inflammatory or tumor conditions, there were some limitations to the study. First, the standard forms and guidelines used in a systematic analysis are only applicable for clinical or animal trials. Consequently, the quality of the retrieved studies and publication bias were not taken into account here, as this would be methodologically inappropriate. As such, large scale animal studies and meta-analyses evaluating the antiangiogenic activity of flavonoids are much needed in the future to provide more definitive conclusions about the role of flavonoids in angiogenesis.

Second, despite subgrouping flavonoids based on their chemical class and using the random effects model, heterogeneity remained high in this study. There are three types of heterogeneity as defined by the Cochrane handbook for systematic reviews, (i) clinical: differences in participants, interventions or outcomes, (ii) methodological: differences in study design, risk of bias and (iii) statistical: variation in intervention effects or results [188]. Looking deeper into the generated forest plots we concluded the cause of heterogeneity to be clinical and/or statistical. This is mainly because most of the flavonoids in a single subgroup are from the same study hence methodological heterogeneity was excluded. This was further supported by the fact that no single flavonoid was found to solely contribute to the heterogeneity when applying the leave-one-out strategy in the sensitivity analysis. In that case, heterogeneity is mainly due to the different flavonoids used in the study (variation in interventions) in addition to other factors like variable outcomes (number of blood vessels). This clinical heterogeneity can lead to a statistical heterogeneity manifested as a variation among the effects or results (ratio of means of number of blood vessels).

## 4. Materials and Methods

This review and meta-analysis were conducted according to Preferred Reporting Items for systematic reviews and Meta Analyses (PRISMA) guidelines [189].

### 4.1. Search Strategy

For Section 1, a literature search was conducted using ScienceDirect, PubMed and Web of Science databases between 3 April 2020 and 23 April 2020 with no time limits. The first set of keywords, (flavonoid, flavone, flavonol, flavanol, anthocyanidin, polyphenol) was combined systematically using the Boolean operator AND with the second set, (angiogenesis, antiangiogenic, proangiogenic, “cell migration”, “wound healing”) in all databases (Table S5).

With regards to the detailed meta-analysis for Section 2, the literature search was carried out using ScienceDirect, PubMed, Web of Science and Google Scholar databases between 8 June 2020 and 10 June 2020 with no time limits. The first set of keywords, (flavonoid, flavone, flavonol, flavanol, anthocyanidin, polyphenol) was combined systematically using the Boolean operator AND with the second set, (angiogenesis, “chick chorioallantoic membrane”, “in vivo angiogenesis”) in all databases (Table S6).

### 4.2. Inclusion and Exclusion Criteria

Studies were included in the Section 1 overview search if they met the following eligibility criteria: (i) natural or synthetic flavonoids (ii) in vitro, in vivo and/or ex vivo angiogenesis assays (iii) focus on cancer, diabetes, bone regeneration or eye diseases. For the meta-analysis Section 2, the inclusion criteria were: (i) natural or synthetic flavonoids (ii) in vivo CAM angiogenesis assays. Articles not written in English and/or focusing on chalcones, plant extracts/total flavonoids content, combination of compounds, nanoformulations, prodrugs, neurological disorders or cardiovascular diseases were excluded from both searches. This systematic review and meta-analysis followed PRISMA guidelines (Table S7).

### 4.3. Data Extraction

Initially, articles’ titles and abstracts were screened based on relevance and inclusion/exclusion criteria. Full texts were checked in some cases when abstracts failed to provide a detailed description. Eligible articles were retrieved and data extracted into a specially designed form. The first set of extracted data for Section 1 included title, publication type, year of publication, flavonoid, disease of focus and conducted in vitro and/or in/ex vivo angiogenesis assays. The second set of data were extracted for the meta-analysis Section 2 study and included title, year of publication, flavonoid, angiogenesis promotor, cancer cell line, concentration, time and duration of flavonoid treatment, results representation and number of CAMs used for each test concentration (*n*).

### 4.4. Data Analysis

Means of the number of blood vessels in a CAM relative to control were used as the outcome measure. Concentrations were reported in µM in all analyses except for analysis of wogonoside’s antiangiogenic effect on breast cancer cell lines in which ng/CAM was used. Values are represented as means ratio ± standard error of means (SEM). For studies reporting standard deviation (SD), the SEM was calculated by dividing SD by square root of the corresponding study sample size. Pool effect size was expressed as means ratio and 95% CI and was calculated using the inverse variance (IV) method. The random effects model was used because it accounts for between study variability. Heterogeneity was assessed using Higgins’ *I*^2^ measure where *I*^2^ ≥ 50% indicates substantial heterogeneity [190]. Sensitivity analysis was applied to evaluate the effect of each flavonoid on summary effect size and on heterogeneity. It is based on the sequential removal of one study at a time. Statistical analysis was performed using Review Manager Version 5.1 (The Nordic Cochrane Centre, The Cochrane Collaboration, Copenhagen, Denmark) and Microsoft Excel 2016.

## 5. Conclusions

Despite the promising antiangiogenic activity of flavonoids presented in many literature studies, no flavonoids have reached clinical trials for this application. This systematic review and meta-analysis therefore aimed to provide further insight into this area by evaluating the in vivo antiangiogenic activity of flavonoids as determined by the widely reported, clinically relevant CAM assay. A comprehensive overview of the antiangiogenic activities of flavonoids with regards to the class of flavonoids, pathology and assays used was presented. Results have shown that the biggest fraction of studies focused on the flavone subclass, cancer related angiogenesis, and in vitro assays. Furthermore, an overall evaluation of the in vivo antiangiogenic activity of flavonoids was offered focusing on SAR and mechanistic considerations. Isoflavones, flavonols and flavones were found to be the most active classes of flavonoids where antiangiogenic activity was dose dependent. Several structural features were considered, from which it was concluded that the position of the hydroxyl substituents and the degree of unsaturation are key for high activity. Even though there were some limitations such as the miscellany of the studied flavonoids and the high heterogeneity, this study provided substantial information that will underpin further investigations by addressing current gaps in the literature regarding the antiangiogenic activity of flavonoids, and highlighting their future prospective as potentially clinically active antiangiogenic agents.

## Figures and Tables

**Table 1:** Characteristics of the studies included in Section 1 for flavonols subclass (see Table S1 for all 9 subclasses).

Flavonol	Disease	In Vitro Tests	In/Ex Vivo Tests	Author, Year
Beturetol	Angiogenesis		CAM	Hisanori Hattori, 2011 [18]
Casticin	Cancer *			Shanaya Ramchandani, 2020 [19]
Denticulatain	Lung Cancer		ZFM	Da Song Yang, 2015 [20]
Dihydrokaempferide	Angiogenesis		CAM	Hisanori Hattori, 2011 [18]
Fisetin	Cancer *			Dharambir Kashyap, 2018 [21]
	Cancer *			Thamaraiselvan Rengarajan, 2016 [22]
	Cancer *			Deeba N.Syed, 2016 [23]
	Cancer *			Lall K. Rahul, 2016 [24]
	Breast Cancer	In		Cheng Fang Tsai, 2018 [25]
	Breast Cancer	WH, In		Xu Sun, 2018 [26]
	Breast Cancer	WH, In	Mets in mice	Jie Li, 2018 [27]
	Cervical Cancer	In		Ruey Hwang Chou, 2013 [28]
	Glioma	In		Chien Min Chen, 2015 [29]
	Hepatic Cancer	In		Xiang Feng Liu, 2017 [30]
	Leukemia	In		Anna Klimaszewska-Wiśniewska, 2019 [31]
	Lung Cancer	WH, In		Saba Tabasum, 2019 [32]
	Lung Cancer	WH, In, Ad		Junjian Wang, 2018 [33]
	Prostate Cancer	WH, In, Ad		Chi Sheng Chien, 2010 [34]
	Renal Cancer	In		Yih Shou Hsieh, 2019 [35]
	Retinopathy		RbCN	A M Joussen, 2000 [36]
Galangin	Hepatic Cancer *			Dengyang Fang, 2019 [37]
	Angiogenesis	TF, Ad		Jong Deog Kim, 2006 [38]
	Glioma	TF, In	CAM, MD in mice	Daliang Chen, 2019 [39]
	Glioma	In		Deqiang Lei, 2018 [40]
	Hepatic Cancer	WH, In, Ad		Shang Tao Chien, 2015 [41]
	Ovarian Cancer	TF	CAM	Haizhi Huang, 2015 [42]
	Renal Cancer	WH, In		Jingyi Cao, 2016 [43]
	Renal Cancer	In		Yun Zhu, 2018 [44]
Gossypin	Gastric Cancer	In		Wang Li, 2019 [45]
Herbacetin	Melanoma	In		Lei Li, 2019 [46]
Hyperoside	Arthritis	WH, In	CIAM in mice	Xiang Nan Jin, 2016 [47]
Icariin	Bone disease *			Xin Zhang, 2014 [48]
	Cancer *			Meixia Chen, 2016 [49]
	Angiogenesis	TF, In	RAR	Byung Hee Chung, 2008 [50]
	Esophageal Cancer	In		Zhen Fang Gu, 2017 [51]
	Ovarian Cancer	WH		Pengzhen Wang, 2019 [52]
	Wound healing		EWM in rats	Wangkheirakpam Ramdas Singh, 2019 [53]
Icariside	Cancer *			Meixia Chen, 2016 [49]
	Glioma	WH, In		Kai Quan, 2017 [54]
Isoviolanthin	Hepatic Cancer	WH, In		Shangping Xing, 2018 [55]
Isosakuranetin	Angiogenesis		CAM	Hisanori Hattori, 2011 [18]
Kaempferol	Cancer *			Allen Y. Chen, 2013 [56]
	Cancer *			Dharambir Kashyap, 2017 [57]
	Angiogenesis	WH, TB, In		Hsien Kuo Chin, 2018 [58]
	Angiogenesis	WH, TB	ZFM	Fang Liang, 2015 [59]
	Angiogenesis		CAM	Shigenori Kumazawa, 2013 [60]
	Angiogenesis	TF, Ad		Jong Deog Kim, 2006 [38]
	Diabetes		EWM in rats	Yusuf Özay, 2019 [61]
	Glioma	WH		Vivek Sharma, 2007 [62]
	Glioma	In	Mets in mice	S.C. Shen, 2006 [63]
	Hepatic Cancer	WH, In	Mets in mice	Youyou Qin, 2015 [64]
	Hepatic Cancer	In		Genglong Zhu, 2018 [65]
	Lung Cancer	WH, In		Eunji Jo, 2015 [66]
	Medulloblastoma	Ad		David Labbé, 2009 [67]
	Oral Cancer	In		Chiao Wen Lin, 2013 [68]
	Osteosarcoma	WH, In, Ad		Hui Jye Chen, 2013 [69]
	Ovarian Cancer		CAM	Haitao Luo, 2009 [70]
	Pancreatic Cancer	In		Jungwhoi Lee, 2016 [71]
	Renal Cancer	WH, In	Mets in mice	Tung Wei Hung, 2017 [72]
	Retinal Vascularization	WH, In		Hsiang Wen Chien, 2019 [73]
Kaempferol-3-*O*-[(6-caffeoyl)-β- glucopyranosyl (1→3) α-rhamnopyranoside]-7-*O*-α-rhamnopyranoside	Angiogenesis	WH		Marco Clericuzio, 2012 [74]
Kaempferide	Angiogenesis		CAM	Hisanori Hattori, 2011 [18]
Morin	Arthritis	WH, TB	CIAM in rats	Ni Zeng, 2015 [75]
	Arthritis	WH, TB	CIAM in rats	Mengfan Yue, 2018 [76]
	Leukemia	Ad		Nagaja Capitani, 2019 [77]
	Melanoma	WH		Hua Wen Li, 2016 [78]
Myricetin	Melanoma *			Nam Joo Kang, 2011 [79]
	Angiogenesis	TF, Ad		Jong Deog Kim, 2006 [38]
	Breast Cancer	In	CAM, MD in mice, RAR	Zhiqing Zhou, 2019 [80]
	Breast Cancer	WH, In, Ad	Mets in mice	Yingqian Ci, 2018 [81]
	Glioma	WH, In		Wen Ta Chiu, 2010 [82]
	Hepatic Cancer	In		Noriko Yamada, 2020 [83]
	Hepatic Cancer	WH, In		Hongxin Ma, 2019 [84]
	Lung Cancer	WH, In, Ad		Yuan Wei Shih, 2009 [85]
	Medullobalstoma	In, Ad		David Labbé, 2009 [67]
	Ovarian Cancer	TF	CAM	Haizhi Huang, 2015 [42]
Quercetin	Breast Cancer *			Maryam Ezzati, 2020 [86]
	Cancer *			Si-min Tang, 2020 [87]
	Cancer *			Dharambir Kashyap, 2016 [88]
	Colorectal Cancer *			Saber G. Darband, 2018 [89]
	Angiogenesis	WH, In		Nu Ry Song, 2014 [90]
	Angiogenesis	WH, TB	ZFM	Chen Lin, 2012 [91]
	Angiogenesis	TF, Ad		Jong Deog Kim, 2006 [38]
	Bladder Cancer	WH, In		Yu Hsiang Lee, 2019 [92]
	Breast Cancer	WH		Divyashree Ravishankar, 2015 [93]
	Breast Cancer		MD in mice	Xin Zhao, 2016 [94]
	Breast Cancer		CAM	Soo Jin Oh, 2010 [95]
	Breast Cancer	WH, In		Asha Srinivasan, 2016 [96]
	Breast Cancer	WH, In		Cheng Wei Lin, 2008 [97]
	Breast Cancer	In		Amilcar Rivera Rivera, 2016 [98]
	Cancer	TF	ZFM	Daxian Zhao, 2014 [99]
	Cancer	TF, In	CAM	Wen Fu Tan, 2003 [100]
	Cancer		MD in mice	Xiangpei Zhao, 2012 [101]
	Cancer	WH, In		Lung Ta Lee, 2004 [102]
	Cancer	WH		Dong Eun Lee, 2013 [103]
	Colorectal Cancer	WH, In	Mets in mice	Ji Ye Kee, 2016 [104]
	Glioma	WH		Hong Chao Pan, 2015 [105]
	Glioma	WH, In		Wen Ta Chiu, 2010 [82]
	Glioma	WH, In		Yue Liu, 2017 [106]
	Glioma	In		Jonathan Michaud-Levesque, 2012 [107]
	Glioma	WH		Alessandra Bispo da Silva, 2020 [108]
	Glioma	WH, TB, In		Yue Liu, 2017 [109]
	Hepatic Cancer	In		Noriko Yamada, 2020 [83]
	Hepatic Cancer	WH, In		Jun Lu, 2018 [110]
	Lung Cancer	WH		Anna Klimaszewska-Wiśniewska, 2017 [111]
	Lung Cancer	In		Tzu Chin Wu, 2018 [112]
	Lung Cancer	In		Yo Chuen Lin, 2013 [113]
	Medulloblastoma	In, Ad		David Labbé, 2009 [67]
	Melanoma	In		Mun Kyung Hwang, 2009 [114]
	Melanoma	In		Hui Hui Cao, 2015 [115]
	Melanoma	WH, In	Mets in mice	Hui Hui Cao, 2014 [116]
	Oral Cancer	In		Junfang Zhao, 2019 [117]
	Osteoblasts	In		Tae Wook Nam, 2008 [118]
	Osteosarcoma	WH, In, Ad		Shenglong Li, 2019 [119]
	Osteosarcoma	WH, In	Mets in mice	Haifeng Lan, 2017 [120]
	Osteosarcoma	WH, Ad		Kersten Berndt, 2013 [121]
	Pancreatic Cancer	WH, In		Ying Tang Huang, 2005 [122]
	Pancreatic Cancer	WH, In		Yu Dinglai 2017 [123]
	Prostate Cancer	WH, In		Firdous Ahmad Bhat, 2014 [124]
	Prostate Cancer	TF, In	MD in mice	Feiya Yang, 2016 [125]
	Retinoblastoma	In		Wei Song, 2017 [126]
Quercetin-3-*O*-[(6-caffeoyl)-β-glucopyranosyl(1→3) α-rhamnopyranoside]-7-*O*-α-rhamnopyranoside	Angiogenesis	WH		Marco Clericuzio, 2012 [74]
Rutin	Angiogenesis		CAM	César Muñoz Camero, 2018 [127]
	Angiogenesis		CAM	Shigenori Kumazawa, 2013 [60]
	Cancer	WH, In, Ad		Mohamed ben Sghaier, 2016 [128]
	Glioma	WH		Alessandra Bispo da Silva, 2020 [108]
	Neuroblastoma	WH, In		Hongyan Chen, 2013 [129]

* Review article; TB, Tube Formation; WH, Wound Healing; In, Invasion; Ad, Adhesion; Mets, Metastasis; CAM, Chick Chorioallantoic Membrane; MPA, Matrigel Plug Assay; RAR, Rat Aortic Ring; EWM, Excision Wound Model; SF, Skin Flap; RRN, Rat Retinal Neovascularization; MAR, Mice Aortic Ring; MD, Microvessel Density; MRN, Mice Retinal Neovascularization; MCN, Mice Corneal Neovascularization; RbCN, Rabbit Corneal Neovascularization; ZFM, Zebra Fish Model; RCN, Rat Corneal Neovascularization; CIAM, Collagen Induced Arthritis Model; DASM, Dorsal air Sac Model; IWM, Incision Wound Model.

**Table 2:** Characteristics of the studies included in Section 2.

Author, Year	Flavonoid	Angiogenesis Promoter	Cell Line	Concentration	Time, Duration of Treatment	Results Representation	*n*
Soo Jin Oh, 2010 [95]	Quercetin	NA	TAMR-MCF-7	3, 10, 30 µM	NA	Number of branches	5 to 7
Chiu-Mei Lin, 2006 [130]	Wogonin	LPS (1µg/mL)	NA	10^−5^, 10^−6^, 10^−7^, 10^−8^ M	10th day, 48 h	Percentage of vascular counts (%)	3
Ling-Zhi Liu, 2011 [131]	Acacetin	NA	OVCAR-3	10 µM	9th day, 4 days	Relative angiogenesis	5
Kai Zhao, 2018 [132]	Wogonin, LW-215	NA	NA	Wogonin: 80 ng/CAM, LW-215: 2, 4, 8 ng/CAM	10th day, 48 h	The number of new vessels (% of control)	3
Haizhi Huang, 2015 [42]	Galangin, myricetin	NA	OVCAR-3	G: 40 µM, M: 20 µM	9th day, 5 days	Blood vessels (%)	6
Olga Viegas, 2019 [133]	Cyanidin, C-3-*O*-glucoside, delphinidin, D-3-*O*-glucoside	NA	NA	20, 40, 80, 100, 200 µM	11th day, 48 h	% of control	5
Wen-fu Tan, 2003 [100]	Quercetin	NA	NA	25, 50, 100 nmol/10 µL/CAM	10th day, 48 h	Microscopic pictures	10
Rajesh Gacche, 2010 [134]	Flavone, 3/5/6/7/-Hydroxy flavone	NA	NA	10, 50, 100 µM	10th day, 48 h	Antiangiogenic activity (%) of selected flavonoids	8
R.N. Gacche, 2011 [135]	3, 6-DHF, 3, 7-DHF, 5, 7-DHF, apigenin, genistein, kaempferol, luteolin, fisetin, rutin, quercetin	NA	NA	10, 50, 100 µM in 0.05% DMSO/20 µL/CAM	10th day, 48 h	Antiangiogenic activity (%) of selected flavonoids	8
R.N. Gacche, 2015 [136]	4′-Methoxy flavone, 3-Hydroxy-7-methoxy flavone, Formononetin, Biochanin-A, Diosmin, Hesperitin, Hesperidin, 2′-Hydroxy flavanone, 4′-Hydroxy flavanone, 7-Hydroxy flavanone, Myricetin, Taxifolin, Silibinin, Silymarin, Naringenin, Naringin, Catechin	NA	NA	10, 50, 100 µM in 0.05% DMSO/20 µL/CAM	10th day, 48 h	Antiangiogenic activity (%) of selected flavonoids	8
Yan Chen, 2010 [137]	LYG-202	NA	NA	2.4, 12, 60 ng/CAM	10th day, 48 h	Percentage of vascular counts (% of control)	10
Hisanori Hattori, 2011 [18]	Beturetol, isosakuranetin	NA	NA	300 ng/CAM	5th day, 7 days	Inhibition % of angiogenesis at 300 ng/CAM.	10
Yujie Huang, 2019 [138]	Wogonoside	NA	MDA-MB-231, MDA-MB-468	50, 100, 200 ng/CAM	10th day, 48 h	Number of new vessels (% cells)	3
Yan Chen, 2009 [139]	Wogonoside	LPS (1µg/mL)	NA	1.5, 15, 150 ng/CAM	10th day, 48 h	Number of vessels (% of LPS)	10
Xiaobo Li, 2017 [140]	Luteolin	Gas6 (300 ng/mL)	NA	10, 20 µM	6th day, 48 h	Relative vascular density (% of control)	3
Siva Prasad Panda, 2019 [141]	TMF	NA	EAT	10, 17, 25 µg/mL	5th day, 11 days	Microscopic pictures	5
Yujie Huang, 2016 [142]	Wogonoside	NA	MCF-7	50, 100, 200 ng/CAM	10th day, 48 h	Number of new vessels (% MCF-7)	3
Tariq A. Bhat, 2013 [143]	Acacetin	NA	NA	50 µM	6th day, (every 48 h for 8 days)	% capillary formation	5 independent areas on CAMs for each treatment
Jing Fang, 2007 [144]	Apigenin	NA	OVCAR-3, PC-3	OVCAR-3: 7.5, 15 µM, PC-3: 10, 20 µM	9th day, 4 days	Quantification of blood vessels on the CAM	8
Jianchu Chen, 2015 [145]	Nobiletin	NA	A2780	20 µM	9th day, 5 days	Blood vessel count	10
Poyil Pratheeshkumar, 2012 [146]	Luteolin	NA	NA	20, 40 µM	8th day, 48 h	Relative vascular density	3
Chiu-Mei Lin, 2006 [147]	Wogonin	IL-6 (10 ng/mL)	NA	10^−5^, 10^−6^, 10^−7^, 10^−8^ M	10th day, 48 h	Percentage of vascular count (%)	3
Dongqing Zhu, 2016 [148]	Baicalin, baicalein	NA	NA	0.5, 2, 10, 50 µg/mL and 0.2, 1, 5 mg/mL	7.5th day, 48 h	Number of new blood vessels	30
Haitao Luo, 2009 [70]	Kaempferol	NA	OVCAR-3	20 µM	9th day, 5 days	Blood vessel count	5
Laure Favot, 2003 [149]	Delphinidin	NA	NA	2, 10, 25, 50 µg	8th day, 48 h	Microscopic pictures	5

*n* = number of CAMs used in each experiment; NA, Not available; DHF, Dihydroxyflavone; TMF, Trimethoxyflavonoid; TMAR, Tamoxifen breast cancer resistant cell line; MCF-7, Breast cancer cell line; LPS, Lipopolysaccharide; OVCAR-3, Ovarian cancer cell line; MDA-MB-231, MDA-MB-468, Triple negative breast cancer cell lines; Gas6, Growth arrest specific 6; EAT, Mouse breast carcinoma (Ehrlich-Lettre Ascites); PC-3, Prostate cancer cell line; A2780, ovarian cancer cell line; IL-6, Interleukin 6.

## Supplementary Materials

The following are available online, Table S1: Study characteristics of Section 1, Tables S2–S4: Sensitivity analysis, Tables S5 and S6: Database search results, Table S7: PRISMA checklist.
