# Supplementary material for: Antiangiogenic Activity of Flavonoids: A Systematic Review and Meta-Analysis
Source: Molecules. 2020 Oct 14;25(20):4712. doi: 10.3390/molecules25204712 (PMC7594036; doi:10.3390/molecules25204712)
Supplement: Supplementary file 1 [file molecules-25-04712-s001.zip › Table S1.docx]

**Table 1.** Characteristics of the studies included in section 1.

| **Class** | **Flavonoid** | **Disease** | ***In vitro* tests** | ***In/ex vivo* tests** | **Author, year** |
| --- | --- | --- | --- | --- | --- |
| Flavones | 3-Hydroxyflavone | Angiogenesis | TF |  | Myoung H. Kim, 2003 [1] |
|  |  | Osteosarcoma | WH, In, Ad | Mets in mice | Ko Hsiu Lu, 2016 [2] |
|  | 5-Hydroxyflavone | Oral Cancer |  | CAM | Shun Fa Yang, 2008 [3] |
|  | 7-Hydroxyflavone |  |  |  |  |
|  | 5, 6, 7-Trihydroxyflavone |  |  |  |  |
|  | 4’, 5, 7 -Trihydroxyflavone |  |  |  |  |
|  | 5,6,7-Trimethoxyflavonoid salicylate | Gastric Cancer | WH |  | Renbo Liu, 2020 [4] |
|  | 7,8-Dihydroxyflavone | Melanoma | WH, In |  | Deok Yong Sim, 2016 [5] |
|  | Acacetin | Angiogenesis |  | CAM | Ling Zhi Liu, 2011[6] |
|  |  | Cancer | TF, In | CAM, MPA in mice, RAR | Tariq A. Bhat, 2014 [7] |
|  |  | Lung Cancer | In, Ad |  | Yaou Fong, 2010 [8] |
|  |  | Lung cancer | WH, In, Ad |  | Shang Tao Chien, 2011 [9] |
|  |  | Prostate Cancer | WH, In |  | Kun Hung Shen, 2010 [10] |
|  | Apigenin | Cancer* |  |  | Dharambir Kashyap, 2018 [11] |
|  |  | Cancer* |  |  | Deendayal Patel, 2007 [12] |
|  |  | Gastric Cancer* |  |  | Émilie C.Lefort, 2013 [13] |
|  |  | Angiogenesis | TF, In |  | Sylvie Lamy, 2012 [14] |
|  |  | Angiogenesis | TF |  | Myoung H. Kim, 2003 [1] |
|  |  | Breast Cancer | In, Ad |  | F. Lindenmeyer, 2011 [15] |
|  |  | Breast Cancer | WH, In, Ad |  | Wei Jiunn Lee, 2008 [16] |
|  |  | Cancer |  | CAM, MPA in mice | Jing Fang, 2007 [17] |
|  |  | Cancer | WH, In |  | Lei Wang, 2013 [18] |
|  |  | Cancer | WH |  | Cornelia Spoerlein, 2013 [19] |
|  |  | Cervical Cancer | In |  | Jaroslaw Czyz, 2005 [20] |
|  |  | Choriocarcinoma | In |  | Whasun Lim, 2016 [21] |
|  |  | Colorectal Cancer | WH, In | Mets in mice | Li Chunhua, 2013 [22] |
|  |  | Colorectal Cancer | WH, In | Mets in mice | Jin Dai, 2016 [23] |
|  |  | Glioma | WH |  | Paulo L.C. Coelho, 2016 [24] |
|  |  | Glioma | WH |  | Paulo L.C. Coelho, 2019 [25] |
|  |  | Lung Cancer |  | MPA in mice | Ling Zhi Liu, 2005 [26] |
|  |  | Melanoma | In |  | Md Abul Hasnat, 2015 [27] |
|  |  | Melanoma | WH, In |  | Guangming Zhao, 2017 [28] |
|  |  | Melanoma | WH, In, Ad | Mets in mice | Hui Hui Cao, 2016 [29] |
|  |  | Melanoma | WH | CAM | Alexandra Ghițu, 2019 [30] |
|  |  | Ovarian Cancer | TF |  | Jing Fang, 2005 [31] |
|  |  | Ovarian Cancer | WH, In, Ad | Mets in mice | Xiao Men Hu, 2008 [32] |
|  |  | Pancreatic Cancer | WH |  | Jing He, 2015 [33] |
|  |  | Prostate Cancer | WH |  | Suat Erdogan, 2016 [34] |
|  |  | Prostate Cancer | WH, In | Mets in mice | Ming Hsien Chien, 2019 [35] |
|  |  | Prostate Cancer | WH, In, Ad |  | Carrie A. Franzen, 2019 [36] |
|  |  | Prostate Cancer | WH | MPA in mice | Sanjeev Shukla, 2015 [37] |
|  | Apigenin-7-*O*-glucoside | Angiogenesis |  | CAM | César Muñoz Camero, 2018 [38] |
|  | Artocarpin | Wound healing | WH, TB, In | EWM in mice | Chung Ju Yeh, 2017 [39] |
|  | Baicalein | Cancer* |  |  | Gao Ying, 2016 [40] |
|  |  | Angiogenesis | WH, TB | CAM, RAR | Yun Ling, 2011 [41] |
|  |  | Breast Cancer | WH, In | Mets in mice | Xing Cong Ma, 2016 [42] |
|  |  | Breast Cancer | WH, In, Ad |  | Ling Wang, 2010 [43] |
|  |  | Breast Cancer | WH, In, Ad |  | Dandan Shang, 2015 [44] |
|  |  | Breast Cancer | In |  | Heesung Chung, 2015 [45] |
|  |  | Colorectal Cancer | In |  | Yuxia Chai, 2017 [46] |
|  |  | Colorectal Cancer | In |  | C. Lalou, 2013 [47] |
|  |  | Gastric Cancer | WH, In |  | Fenglin Chen, 2014 [48] |
|  |  | Glioma | WH, In |  | Wen Ta Chiu, 2010 [49] |
|  |  | Hepatic Cancer | WH, In, Ad |  | Yung Wei Chiu, 2011 [50] |
|  |  | Hepatic Cancer | In |  | Xiaolan Yu, 2018 [51] |
|  |  | Lung Cancer | WH, TB | Mets in mice | Yang Gyu Park, 2017 [52] |
|  |  | Melanoma | WH, In |  | Eun Ok Choi, 2017 [53] |
|  |  | Osteosarcoma | In |  | Jian Zhang, 2018 [54] |
|  |  | Osteosarcoma | In, Ad |  | Yi Zhang, 2013 [55] |
|  |  | Pancreatic Cancer | WH, In |  | Rong Tao Zhou, 2017 [56] |
|  |  | Wound healing |  | SF in rats | Renjin Lin, 2018 [57] |
|  | Baicalin | Breast Cancer | In |  | Heesung Chung, 2015 [45] |
|  |  | Breast Cancer | WH, In |  | Tao Zhou, 2017 [58] |
|  |  | Colorectal Cancer | WH, In, Ad | Mets in mice | Bolin Yang, 2020 [59] |
|  |  | Glioma | In |  | Yihao Zhu, 2018 [60] |
|  |  | Hepatic Cancer | In |  | Xiongjian Wu, 2018 [61] |
|  |  | Lung Cancer | WH, In |  | Jiawen You, 2018 [62] |
|  |  | Ovarian Cancer | WH, In |  | Chen Gao, 2017 [63] |
|  |  | Retinopathy |  | RRN | Hyoung Jo, 2015 [64] |
|  | Chrysin | Cancer* |  |  | Eshvendar Reddy Kasala, 2015 [65] |
|  |  | Angiogenesis | WH | MAR | Sha-sha Tian, 2014 [66] |
|  |  | Breast Cancer | WH, In |  | Bing Yang, 2014 [67] |
|  |  | Cancer |  | MD in mice | Xiangpei Zhao, 2012 [68] |
|  |  | Cancer | WH |  | Cornelia Spoerlein, 2013 [19] |
|  |  | Colorectal Cancer | In |  | C. Lalou, 2013 [47] |
|  |  | Gastric Cancer | In |  | Yong Xia, 2015 [69] |
|  |  | Lung Cancer | In |  | Tzu Chin Wu, 2018 [70] |
|  |  | Prostate Cancer |  | MPA in mice | Beibei Fu, 2007 [71] |
|  |  | Retinopathy |  | MRN | Min Kyung Kang, 2016 [72] |
|  | Chrysoeriol | Breast Cancer | In |  | K. Amrutha, 2014 [73] |
|  | Cirsiliol | Melanoma | WH, In |  | Priyanka Prasad, 2019 [74] |
|  | Didymin | Angiogenesis | WH, TB, In | MPA in mice, MAR | Kirtikar Shukla, 2019 [75] |
|  |  | Neuroblastoma | WH |  | Jyotsana Singhal, 2012 [76] |
|  | Diosmetin | Glioma | WH, In |  | Yuli Yan, 2020 [77] |
|  |  | Lung Cancer | WH |  | Yuli Yan, 2020 [78] |
|  |  | Melanoma | WH, TB | MD in mice, RAR | Jawun Choi, 2019 [79] |
|  | Eupafolin | Hepatic Cancer | WH, TB, In | MPA in mice, MD in mice | Honglei Jiang, 2017 [80] |
|  | Eupatilin | Gastric Cancer | TF |  | Jae Ho Cheong, 2011 [81] |
|  |  | Glioma | WH, In |  | Xiaowei Fei, 2019 [82] |
|  | Eupatorin | Cancer | WH, TB |  | Iva Dolečková, 2012 [83] |
|  | Flavone-6-C-*β*-D-glucopyranoside | Ovarian Cancer | In |  | Yuanda Du, 2015 [84] |
|  | Flavopiridol | Lung Cancer | WH |  | Irem Dogan Turacli, 2019 [85] |
|  | GL-V9 | Breast Cancer | WH, In, Ad |  | Liwen Li, 2011 [86] |
|  | Hispidulin | Colon Cancer | WH, In | E-cadherin level inhibition in mice tumor | Jing Xie, 2015 [87] |
|  |  | Hepatic Cancer | WH, In | Mets in mice | Mei Han, 2018 [88] |
|  |  | Pancreatic Cancer | WH, TB, In | MCN,MD in mice, RAR | Lijun He, 2011 [89] |
|  |  | Renal Cancer | In | Mets in mice | Ming Quan Gao, 2017 [90] |
|  | LFG-500 | Breast Cancer | WH, In, Ad | Mets in mice | Chenglin Li, 2014 [91] |
|  | Linarin | Glioma | WH, In |  | Zi Gang Zhen, 2017 [92] |
|  | Luteolin | Breast Cancer* |  |  | Matthew T Cook, 2018 [93] |
|  |  | Angiogenesis | WH, TB, In | CAM, RAR | Xiaobo Li, 2017 [94] |
|  |  | Angiogenesis | In |  | Manyi Zhu, 2013 [95] |
|  |  | Angiogenesis | TF, In |  | Sylvie Lamy, 2012 [14] |
|  |  | Breast Cancer | WH |  | Divyashree Ravishankar, 2015 [96] |
|  |  | Breast Cancer | WH, In | Mets in mice | Hongyuan Li, 2017 [97] |
|  |  | Cancer (breast and colon) | WH, In, Ad | Mets in mice | Luciana G.Naso, 2016 [98] |
|  |  | Cancer | WH, In |  | Lung Ta Lee, 2004 [99] |
|  |  | Cancer | WH, In |  | Samir Attoub, 2011 [100] |
|  |  | Cancer (epidermal) |  | RbCN, MD in mice | Eleni Bagli, 2004 [101] |
|  |  | Colorectal | WH, In | Mets in mice | Yuanyuan Yao, 2019 [102] |
|  |  | Diabetes |  | EWM in rats | Santram Lodhi, 2013 [103] |
|  |  | Gastric Cancer | In |  | Jun Lu, 2015 [104] |
|  |  | Gastric Cancer | In |  | Ming de Zang, 2017 [105] |
|  |  | Gastric Cancer | In | Mets in mice | Yansong Pu, 2018 [106] |
|  |  | Glioma | WH |  | Qiang Wang, 2017 [107] |
|  |  | Glioma | WH |  | Ruthrotha B.Selvi, 2015 [108] |
|  |  | Glioma | WH, In |  | Wen Yu Cheng, 2013 [109] |
|  |  | Glioma | WH |  | Yollanda E. M. Franco, 2020 [110] |
|  |  | Hepatic Cancer | In |  | Wei Jiunn Lee, 2006 [111] |
|  |  | Lung Caner | WH, In |  | Guanmin Meng, 2016 [112] |
|  |  | Lung Cancer | In, Ad |  | Junshan Ruan, 2012 [113] |
|  |  | Lung Cancer | In |  | Yo Chuen Lin, 2013 [114] |
|  |  | Melanoma | WH, TB, In, Ad |  | Chunyu Li, 2019 [115] |
|  |  | Melanoma | WH, In |  | Xin Yao, 2019 [116] |
|  |  | Melanoma | In, Ad | Mets in mice | Jun Shan Ruan, 2012 [117] |
|  |  | Oral Cancer | WH, In |  | Bharath Kumar Velmurugan, 2020 [118] |
|  |  | Pancreatic Cancer | WH, In |  | Ying Tang Huang, 2005 [119] |
|  |  | Prostate Cancer | WH, TB, In | CAM, RAR | Poyil Pratheeshkumar, 2012 [120] |
|  |  | Retinopathy |  | RbCN | A M Joussen, 2000 [121] |
|  | luteolin-3’-*O*-*β*-D-glucopyranoside | Ovarian Cancer | In |  | Yuanda Du, 2015 [84] |
|  | Luteoloside | Hepatic Cancer | WH, In | Mets in mice | Shao Hua Fan, 2014 [122] |
|  | LW-215 | Angiogenesis | TF, In | CAM, RAR | Kai Zhao, 2018 [123] |
|  | LYG-202 | Angiogenesis | TF, In | CAM, RAR | Yan Chen, 2010 [124] |
|  |  | Breast Cancer | TF, In | CAM, MPA in mice, MD in mice, RAR | Kai Zhao, 2018 [125] |
|  | Morusin | Hepatic Cancer | WH, In, Ad | Mets in mice | Wea Lung Lin, 2015 [126] |
|  | Nobiletin | Angiogenesis | TF | ZFM | Kai Heng Lam, 2011 [127] |
|  |  | Breast Cancer | WH, TB, In |  | S. P. Nipin, 2017 [128] |
|  |  | Breast Cancer | WH, TB, In |  | S. P. Nipin, 2018 [129] |
|  |  | Breast Cancer | WH |  | Jianli Liu, 2018 [130] |
|  |  | Gastric Cancer | WH, In, Ad |  | Yi Chieh Lee, 2011 [131] |
|  |  | Glioma | WH, In |  | Li Ming Lien, 2016 [132] |
|  |  | Glioma | WH, In |  | Xiang Zhang, 2017 [133] |
|  |  | Hepatic Cancer | In, Ad |  | Ming Der Shi, 2013 [134] |
|  |  | Lung Cancer | In, Ad | Mets in mice | Chunli Da, 2016 [135] |
|  |  | Ovarian Cancer |  | CAM | Jianchu Chen, 2015 [136] |
|  |  | Renal Cancer | In |  | Feng Liu, 2019 [137] |
|  |  | Wound healing |  | SF in mice | Renhao Jiang, 2020 [138] |
|  | Orientin | Breast Cancer | WH |  | Soo Jin Kim, 2018 [139] |
|  | Isoorientin | Lung Cancer | WH |  | Hsu Kai Huang, 2020 [140] |
|  | Oroxylin | Breast Cancer | WH, In, Ad | Mets in mice | Zhijian Lu, 2012 [141] |
|  |  | Colorectal Cancer | In |  | C.Lalou, 2013 [47] |
|  | Oroxylin A 7*β*- glucuronomide | Colorectal Cancer | In |  | C.Lalou, 2013 [47] |
|  | Pectolinarigenin | Breast Cancer | WH, In | Mets in mice | Yali Li, 2019 [142] |
|  |  | Colorectal | WH, In | Mets in mice | Cailing Gan, 2019 [143] |
|  |  | Hepatic Cancer | WH, In |  | Sheng Liu, 2020 [144] |
|  | Radix Tetrastigma hemsleyani flavone (RTHF) | Colorectal Cancer | WH, In |  | Xiaowei Wu, 2018 [145] |
|  | Scultellarin | Angiogenesis | WH, TB |  | Zhong Xiu Zi Gao, 2010 [146] |
|  |  | Bladder Cancer | In | Mets in mice | Wei Ling Lv, 2019 [147] |
|  |  | Glioma | In |  | Shi Lei Tang, 2019 [148] |
|  |  | Hepatic Cancer | WH, In | Mets in mice | Yang Ke, 2017 [149] |
|  |  | Melanoma | WH, TB, In, Ad |  | Chun Yu Li, 2019 [150] |
|  |  | Renal Cancer | In |  | Wenting Deng, 2018 [151] |
|  | Scultellarein | Cancer |  | CAM, RCN | Prabhu Thirusangu, 2017 [152] |
|  | Tricetin | Oral Cancer | In |  | Tsung Te Chung, 2017 [153] |
|  |  | Osteosarcoma | WH, In |  | Pin Yu Chang, 2017 [154] |
|  | Tricin | Glioma | WH, In |  | Dai Jung Chung, 2018 [155] |
|  |  | Hepatic Cancer | In |  | Naoko Seki, 2012 [156] |
|  | VI-14 | Breast Cancer | WH, In, Ad |  | Fanni Li, 2012 [157] |
|  | Vicenin | Lung Cancer | WH, In |  | Yingyi Luo, 2019 [158] |
|  | Vitexin | Pheochromocytoma | TF, In |  | Hwa Jung Choi, 2006 [159] |
|  | Wogonin | Cancer* |  |  | Do Luong Huynh, 2017 [160] |
|  |  | Angiogenesis | WH | MAR | Sha-sha Tian, 2014 [66] |
|  |  | Angiogenesis | TF, In | CAM | Chiu Mei Lin, 2006 [161] |
|  |  | Cancer | TF, In | CAM, MD in mice, RAR | Xiuming Song, 2013 [162] |
|  |  | Glioma | In | Mets in mice | S. C. Shen, 2006 [163] |
|  |  | Myeloma | WH, TB, In | MD in mice | Rong Fu, 2016 [164] |
|  |  | Pancreatic Cancer | WH, In |  | Ying Tang Huang, 2005 [119] |
|  | Wogonoside | Angiogenesis | WH, TB | CAM, RAR | Yan Chen, 2009 [165] |
|  |  | Breast Cancer | WH, TB | CAM, MD in mice, RAR | Yujie Huang, 2016 [166] |
|  |  | Endometrial Cancer | In | Mets in mice | Shaorong Chen, 2019 [167] |
|  | WYC02-9 | Cervical Cancer | WH, TB, In | ZFM, MPA in mice, MD in mice | Yun-ju Chen, 2013 [168] |
| Flavonols | Beturetol | Angiogenesis |  | CAM | Hisanori Hattori, 2011 [169] |
|  | Casticin | Cancer* |  |  | Shanaya Ramchandani, 2020 [170] |
|  | Denticulatain | Lung Cancer |  | ZFM | Da Song Yang, 2015 [171] |
|  | Dihydrokaempferide | Angiogenesis |  | CAM | Hisanori Hattori, 2011 [169] |
|  | Fisetin | Cancer* |  |  | Dharambir Kashyap, 2018 [172] |
|  |  | Cancer* |  |  | Thamaraiselvan Rengarajan, 2016 [173] |
|  |  | Cancer* |  |  | Deeba N.Syed, 2016 [174] |
|  |  | Cancer* |  |  | Lall K. Rahul, 2016 [175] |
|  |  | Breast Cancer | In |  | Cheng Fang Tsai, 2018 [176] |
|  |  | Breast Cancer | WH, In |  | Xu Sun, 2018 [177] |
|  |  | Breast Cancer | WH, In | Mets in mice | Jie Li, 2018 [178] |
|  |  | Cervical Cancer | In |  | Ruey Hwang Chou, 2013 [179] |
|  |  | Glioma | In |  | Chien Min Chen, 2015 [180] |
|  |  | Hepatic Cancer | In |  | Xiang Feng Liu, 2017 [181] |
|  |  | Leukemia | In |  | Anna Klimaszewska‑Wiśniewska, 2019 [182] |
|  |  | Lung Cancer | WH, In |  | Saba Tabasum, 2019 [183] |
|  |  | Lung Cancer | WH, In, Ad |  | Junjian Wang, 2018 [184] |
|  |  | Prostate Cancer | WH, In, Ad |  | Chi Sheng Chien, 2010 [185] |
|  |  | Renal Cancer | In |  | Yih Shou Hsieh, 2019 [186] |
|  |  | Retinopathy |  | RbCN | A M Joussen, 2000 [121] |
|  | Galangin | Hepatic Cancer* |  |  | Dengyang Fang, 2019 [187] |
|  |  | Angiogenesis | TF, Ad |  | Jong Deog Kim, 2006 [188] |
|  |  | Glioma | TF, In | CAM, MD in mice | Daliang Chen, 2019 [189] |
|  |  | Glioma | In |  | Deqiang Lei, 2018 [190] |
|  |  | Hepatic Cancer | WH, In, Ad |  | Shang Tao Chien, 2015 [191] |
|  |  | Ovarian Cancer | TF | CAM | Haizhi Huang, 2015 [192] |
|  |  | Renal Cancer | WH, In |  | Jingyi Cao, 2016 [193] |
|  |  | Renal Cancer | In |  | Yun Zhu, 2018 [194] |
|  | Gossypin | Gastric Cancer | In |  | Wang Li, 2019 [195] |
|  | Herbacetin | Melanoma | In |  | Lei Li, 2019 [196] |
|  | Hyperoside | Arthritis | WH, In | CIAM in mice | Xiang Nan Jin, 2016 [197] |
|  | Icariin | Bone disease* |  |  | Xin Zhang, 2014 [198] |
|  |  | Cancer* |  |  | Meixia Chen, 2016 [199] |
|  |  | Angiogenesis | TF, In | RAR | Byung Hee Chung, 2008 [200] |
|  |  | Esophageal Cancer | In |  | Zhen Fang Gu, 2017 [201] |
|  |  | Ovarian Cancer | WH |  | Pengzhen Wang, 2019 [202] |
|  |  | Wound healing |  | EWM in rats | Wangkheirakpam Ramdas Singh, 2019 [203] |
|  | Icariside | Cancer* |  |  | Meixia Chen, 2016 [199] |
|  |  | Glioma | WH, In |  | Kai Quan, 2017 [204] |
|  | Isoviolanthin | Hepatic Cancer | WH, In |  | Shangping Xing, 2018 [205] |
|  | Isosakuranetin | Angiogenesis |  | CAM | Hisanori Hattori, 2011 [169] |
|  | Kaempferol | Cancer* |  |  | Allen Y. Chen, 2013 [206] |
|  |  | Cancer* |  |  | Dharambir Kashyap, 2017 [207] |
|  |  | Angiogenesis | WH, TB, In |  | Hsien Kuo Chin, 2018 [208] |
|  |  | Angiogenesis | WH, TB | ZFM | Fang Liang, 2015 [209] |
|  |  | Angiogenesis |  | CAM | Shigenori Kumazawa, 2013 [210] |
|  |  | Angiogenesis | TF, Ad |  | Jong Deog Kim, 2006 [188] |
|  |  | Diabetes |  | EWM in rats | Yusuf Özay, 2019 [211] |
|  |  | Glioma | WH |  | Vivek Sharma, 2007 [212] |
|  |  | Glioma | In | Mets in mice | S.C. Shen, 2006 [163] |
|  |  | Hepatic Cancer | WH, In | Mets in mice | Youyou Qin, 2015 [213] |
|  |  | Hepatic Cancer | In |  | Genglong Zhu, 2018 [214] |
|  |  | Lung Cancer | WH, In |  | Eunji Jo, 2015 [215] |
|  |  | Medulloblastoma | Ad |  | David Labbé, 2009 [216] |
|  |  | Oral Cancer | In |  | Chiao Wen Lin, 2013 [217] |
|  |  | Osteosarcoma | WH, In, Ad |  | Hui Jye Chen, 2013 [218] |
|  |  | Ovarian Cancer |  | CAM | Haitao Luo, 2009 [219] |
|  |  | Pancreatic Cancer | In |  | Jungwhoi Lee, 2016 [220] |
|  |  | Renal Cancer | WH, In | Mets in mice | Tung Wei Hung, 2017 [221] |
|  |  | Retinal Vascularization | WH, In |  | Hsiang Wen Chien, 2019 [222] |
|  | Kaempferol-3-*O*-[(6-caffeoyl)-β- glucopyranosyl (1→3) α-rhamnopyranoside]-7- *O*-α-rhamnopyranoside | Angiogenesis | WH |  | Marco Clericuzio, 2012 [223] |
|  | Kaempferide | Angiogenesis |  | CAM | Hisanori Hattori, 2011 [169] |
|  | Morin | Arthritis | WH, TB | CIAM in rats | Ni Zeng, 2015 [224] |
|  |  | Arthritis | WH, TB | CIAM in rats | Mengfan Yue, 2018 [225] |
|  |  | Leukemia | Ad |  | Nagaja Capitani, 2019 [226] |
|  |  | Melanoma | WH |  | Hua Wen Li, 2016 [227] |
|  | Myricetin | Melanoma* |  |  | Nam Joo Kang, 2011 [228] |
|  |  | Angiogenesis | TF, Ad |  | Jong Deog Kim, 2006 [188] |
|  |  | Breast Cancer | In | CAM, MD in mice, RAR | Zhiqing Zhou, 2019 [229] |
|  |  | Breast Cancer | WH, In, Ad | Mets in mice | Yingqian Ci, 2018 [230] |
|  |  | Glioma | WH, In |  | Wen Ta Chiu, 2010 [49] |
|  |  | Hepatic Cancer | In |  | Noriko Yamada, 2020 [231] |
|  |  | Hepatic Cancer | WH, In |  | Hongxin Ma, 2019 [232] |
|  |  | Lung Cancer | WH, In, Ad |  | Yuan Wei Shih, 2009 [233] |
|  |  | Medullobalstoma | In, Ad |  | David Labbé, 2009 [216] |
|  |  | Ovarian Cancer | TF | CAM | Haizhi Huang, 2015 [192] |
|  | Quercetin | Breast Cancer* |  |  | Maryam Ezzati, 2020 [234] |
|  |  | Cancer* |  |  | Si-min Tang, 2020 [235] |
|  |  | Cancer* |  |  | Dharambir Kashyap, 2016 [236] |
|  |  | Colorectal Cancer* |  |  | Saber G. Darband, 2018 [237] |
|  |  | Angiogenesis | WH, In |  | Nu Ry Song, 2014 [238] |
|  |  | Angiogenesis | WH, TB | ZFM | Chen Lin, 2012 [239] |
|  |  | Angiogenesis | TF, Ad |  | Jong Deog Kim, 2006 [188] |
|  |  | Bladder Cancer | WH, In |  | Yu Hsiang Lee, 2019 [240] |
|  |  | Breast Cancer | WH |  | Divyashree Ravishankar, 2015 [96] |
|  |  | Breast Cancer |  | MD in mice | Xin Zhao, 2016 [241] |
|  |  | Breast Cancer |  | CAM | Soo Jin Oh, 2010 [242] |
|  |  | Breast Cancer | WH, In |  | Asha Srinivasan, 2016 [243] |
|  |  | Breast Cancer | WH, In |  | Cheng Wei Lin, 2008 [244] |
|  |  | Breast Cancer | In |  | Amilcar Rivera Rivera, 2016 [245] |
|  |  | Cancer | TF | ZFM | Daxian Zhao, 2014 [246] |
|  |  | Cancer | TF, In | CAM | Wen Fu Tan, 2003 [247] |
|  |  | Cancer |  | MD in mice | Xiangpei Zhao, 2012 [68] |
|  |  | Cancer | WH, In |  | Lung Ta Lee, 2004 [99] |
|  |  | Cancer | WH |  | Dong Eun Lee, 2013 [248] |
|  |  | Colorectal Cancer | WH, In | Mets in mice | Ji Ye Kee, 2016 [249] |
|  |  | Glioma | WH |  | Hong Chao Pan, 2015 [250] |
|  |  | Glioma | WH, In |  | Wen Ta Chiu, 2010 [49] |
|  |  | Glioma | WH, In |  | Yue Liu, 2017 [251] |
|  |  | Glioma | In |  | Jonathan Michaud-Levesque, 2012 [252] |
|  |  | Glioma | WH |  | Alessandra Bispo da Silva, 2020 [253] |
|  |  | Glioma | WH, TB, In |  | Yue Liu, 2017 [254] |
|  |  | Hepatic Cancer | In |  | Noriko Yamada, 2020 [231] |
|  |  | Hepatic Cancer | WH, In |  | Jun Lu, 2018 [255] |
|  |  | Lung Cancer | WH |  | Anna Klimaszewska-Wiśniewska, 2017 [256] |
|  |  | Lung Cancer | In |  | Tzu Chin Wu, 2018 [70] |
|  |  | Lung Cancer | In |  | Yo Chuen Lin, 2013 [114] |
|  |  | Medulloblastoma | In, Ad |  | David Labbé, 2009 [216] |
|  |  | Melanoma | In |  | Mun Kyung Hwang, 2009 [257] |
|  |  | Melanoma | In |  | Hui Hui Cao, 2015 [258] |
|  |  | Melanoma | WH, In | Mets in mice | Hui Hui Cao, 2014 [259] |
|  |  | Oral Cancer | In |  | Junfang Zhao, 2019 [260] |
|  |  | Osteoblasts | In |  | Tae Wook Nam, 2008 [261] |
|  |  | Osteosarcoma | WH, In, Ad |  | Shenglong Li, 2019 [262] |
|  |  | Osteosarcoma | WH, In | Mets in mice | Haifeng Lan, 2017 [263] |
|  |  | Osteosarcoma | WH, Ad |  | Kersten Berndt, 2013 [264] |
|  |  | Pancreatic Cancer | WH, In |  | Ying Tang Huang, 2005 [119] |
|  |  | Pancreatic Cancer | WH, In |  | Yu Dinglai 2017 [265] |
|  |  | Prostate Cancer | WH, In |  | Firdous Ahmad Bhat, 2014 [266] |
|  |  | Prostate Cancer | TF, In | MD in mice | Feiya Yang, 2016 [267] |
|  |  | Retinoblastoma | In |  | Wei Song, 2017 [268] |
|  | Quercetin-3-*O*-[(6-caffeoyl)-β-glucopyranosyl(1→3) α-rhamnopyranoside]-7-*O*-α-rhamnopyranoside | Angiogenesis | WH |  | Marco Clericuzio, 2012 [223] |
|  | Rutin | Angiogenesis |  | CAM | César Muñoz Camero, 2018 [38] |
|  |  | Angiogenesis |  | CAM | Shigenori Kumazawa, 2013 [210] |
|  |  | Cancer | WH, In, Ad |  | Mohamed ben Sghaier, 2016 [269] |
|  |  | Glioma | WH |  | Alessandra Bispo da Silva, 2020 [253] |
|  |  | Neuroblastoma | WH, In |  | Hongyan Chen, 2013 [270] |
| Flavanones | 2’-Hydroxyflavanone | Breast Cancer | WH |  | Jyotsana Singhal, 2017 [271] |
|  |  | Prostate Cancer | WH, In |  | Shiqi Wu, 2018 [272] |
|  | 7,2’,4’-Triihydroxy-5-methoxy-8-dimethylallylflavanone | Angiogenesis | WH, TB, Ad |  | Xiu Li Zhang, 2013 [273] |
|  | Alpinetin | Ovarian Cancer | WH |  | Xuezhi Zhao, 2018 [274] |
|  | Chamaejasmenin | Breast Cancer | WH, In | Mets in mice | Qi Li, 2016 [275] |
|  | Eriodictyol | Glioma | WH, In |  | Wenjun Li, 2020 [276] |
|  | Hesperidin | Diabetes |  | EWM in rats | Wenbin Li, 2018 [277] |
|  |  | Melanoma | WH | Mets in mice | Eui Baek Byun, 2019 [278] |
|  | HLBT-100 | Cancer |  | RAR | Henry I.C. Lowe, 2017 [279] |
|  | HMFRR | Lung Cancer | WH, In, Ad |  | Qing Shi, 2017 [280] |
|  | Liquiritigenin | Cervical Cancer | TF |  | Si Rou Xie, 2012 [281] |
|  | Naringin | Glioma | WH, In |  | Sonia Aroui, 2016 [282] |
|  |  | Glioma | In |  | Sonia Aroui, 2016 [283] |
|  | Naringenin | Angiogenesis | TF | MPA in mice | Irene Pafumi, 2017 [284] |
|  |  | Cancer |  | MD in mice | Xiangpei Zhao, 2012 [68] |
|  | Pinocembrin | Retinoblastoma | In, Ad |  | Kun Shiang Chen, 2014 [285] |
| Flavanols | Epigallocatechin gallate | Angiogenesis* |  |  | Daniel Karas, 2017 [286] |
|  |  | Cancer* |  |  | Zhe Hou, 2004 [287] |
|  |  | Cancer* |  |  | Vidushi S. Neergheen, 2010 [288] |
|  |  | Cancer* |  |  | Animesh Chowdhury, 2016 [289] |
|  |  | Cancer* |  |  | Michael Xavier Doss, 2005 [290] |
|  |  | Cancer/Diabetes* |  |  | Layth Abdulmajeed Abdulkhaleq, 2017 [291] |
|  |  | Melanoma* |  |  | Suchitra Katiyar, 2007 [292] |
|  |  | Miscellaneous diseases* |  |  | Muhammad Saeed, 2017 [293] |
|  |  | Angiogenesis | TF | MPA in mice | A. K.Singh, 2002 [294] |
|  |  | Angiogenesis | TF, In |  | Jun Shi, 2017 [295] |
|  |  | Bladder Cancer | WH, In |  | Ke Wang Luo, 2017 [296] |
|  |  | Bladder Cancer | WH, In |  | Ke Wang Luo, 2018 [297] |
|  |  | Breast Cancer | WH, In |  | Yimin Zhang, 2009 [298] |
|  |  | Cancer | TF, In | DASM in mice | Satoru Yamakawa, 2004 [299] |
|  |  | Cervical Cancer | In, Ad |  | Oana Tudoran, 2012 [300] |
|  |  | Glioma | In |  | Borhane Annabi, 2002 [301] |
|  |  | Glioma | In |  | Hong Li, 2014 [302] |
|  |  | Hepatic Cancer | WH, In |  | Mao Chuan Zhen, 2006 [303] |
|  |  | Kaposi Sarcoma | In | MPA in mice, MD in mice | Gianfranco Fassina, 2004 [304] |
|  |  | Lung Cancer | TF | MPA in mice | Xiangyong Li, 2013 [305] |
|  |  | Lung Cancer | WH, In |  | Jingli Shi, 2015 [306] |
|  |  | Lung Cancer | In |  | Yea Tzy Deng, 2011 [307] |
|  |  | Medulloblastoma | In, Ad |  | Anthony Pilorget, 2003 [308] |
|  |  | Melanoma | In | MD in mice | Noritaka Ohga, 2009 [309] |
|  |  | Nasopharyngeal Carcinoma | WH, In |  | Chien Hung Lin, 2012 [310] |
|  |  | Nasopharyngeal Carcinoma | In |  | Chih Yeu Fang, 2015 [311] |
|  |  | Nasopharyngeal Carcinoma | WH |  | Chien Hung Lin, 2020 [312] |
|  |  | Neuroblastoma | TF, In |  | Md Motarab Hossain, 2012 [313] |
|  |  | Neuroblastoma | In |  | Spiridione Garbisa, 2001 [314] |
|  |  | Oral Cancer | In |  | Yung Chuan Ho, 2007 [315] |
|  |  | Oral Cancer | WH, In, Ad |  | Pei Ni Chen, 2011 [316] |
|  |  | Osteoblasts | WH, In |  | Tetsu Kawabata, 2018 [317] |
|  |  | Osteoblasts | WH, In |  | Tetsu Kawabata, 2018 [318] |
|  |  | Ovarian Cancer | In | MD in mice | Francesca Spinella, 2006 [319] |
|  |  | Pancreatic Cancer | In |  | Atsushi Masamune, 2005 [320] |
|  |  | Prostate Cancer | In |  | Damian Duhon, 2010 [321] |
|  |  | Retinopathy | In, Ad |  | Chi Ming Chan, 2010 [322] |
|  |  | Retinopathy | TF | MCN | Hak Sung Lee, 2014 [323] |
|  | Epicatechin-3-gallate | Diabetes |  | IWM in rats | Kelly J. McKelvey, 2012 [324] |
|  |  | Lung Cancer | WH, In, Ad |  | Shu Fang Huang, 2016 [325] |
|  | Silymarin | Breast/Prostate Cancer | TF |  | Cheng Jiang, 2000 [326] |
|  | Silibinin | Prostate Cancer* |  |  | Harold Ting, 2013 [327] |
|  |  | Bladder Cancer | In | Mets in mice | Kaijie Wu, 2013 [328] |
|  |  | Bladder Cancer | WH, In |  | Feng Li, 2018 [329] |
|  |  | Breast Cancer | In |  | Mohadeseh Dastpeyman, 2012 [330] |
|  |  | Breast Cancer | WH, In |  | Hyo Joo Byun, 2017 [331] |
|  |  | Colon Cancer | In |  | Chiu Mei Lin, 2012 [332] |
|  |  | Glioma | In |  | Kwang Won Kim, 2009 [333] |
|  |  | Prostate Cancer | In | Mets in mice | Harold Ting, 2013 [334] |
|  |  | Prostate Cancer | TF |  | Gagan Deep, 2017 [335] |
|  |  | Prostate Cancer | In, Ad |  | Mohammad Javad Mokhtari, 2008 [336] |
|  |  | Renal Cancer | In |  | Liang Liang, 2012 [337] |
|  |  | Retinopathy |  | RRN | C. H. Lin, 2013 [338] |
|  | Taxifolin | Osteosarcoma | In |  | Xin Chen, 2018 [339] |
| Flavanes | BAS1 & BAS4 | Glioma | WH |  | L. A.L. Maués, 2019 [340] |
| Isoflavones | Alpinumisoflavone | Melanoma | WH, In | Mets in mice | Ming Gao, 2017 [341] |
|  | Biochanin | Lung Cancer | WH, In |  | Yan Wang, 2018 [342] |
|  | Corylin | Hepatic Cancer | WH, In |  | Chi Yuan Chen, 2018 [343] |
|  | DCMF | Wound healing | WH | EWM in mice | Phorl Sophors, 2016 [344] |
|  | Furowanin | Colorectal Cancer | WH, In | Mets in mice | Jinxia Zhao, 2019 [345] |
|  | Genistein | Cancer* |  |  | Janet M. Pavese, 2010 [346] |
|  |  | Angiogenesis | TF |  | Myoung H.Kim, 2003 [1] |
|  |  | Breast Cancer | In | MD in mice | Z.Shao, 2000 [347] |
|  |  | Cancer | WH |  | Cornelia Spoerlein, 2013 [19] |
|  |  | Hepatic Cancer | WH |  | Qian Zhang, 2019 [348] |
|  |  | Retinopathy |  | RbCN | A M Joussen, 2000 [121] |
|  | 4’, 6, 7-trimethoxyisoflavone (TMF) | Angiogenesis |  | CAM | Siva Prasad Panda, 2020 [349] |
|  |  | Wound healing | WH |  | Ngoc Thuy Bui, 2014 [350] |
| Isoflavanones | Deguelin | Cancer* |  |  | Ying Wang, 2013 [351] |
|  |  | Angiogenesis | TF, In | MPA in mice | Raffaella Dell'Eva, 2007 [352] |
|  |  | Pancreatic Cancer | WH, In |  | Wen Zheng, 2016 [353] |
| Isoflavanes | Glabridin | Breast Cancer | WH, TB, In | MPA in mice | Ya Ling Hsu, 2011 [354] |
|  |  | Lung Cancer | WH, TB, In | MPA in mice | Ying Ming Tsai, 2011 [355] |
| Anthocyanidines | Cyanidin | Angiogenesis | TF |  | Matsunaga Nozomu, 2010 [356] |
|  | Delphinidin | Angiogenesis* |  |  | Kanika Patel, 2013 [357] |
|  |  | Angiogenesis | TF, In | MPA in mice | Sylvie Lamy, 2006 [358] |
|  |  | Angiogenesis | WH | CAM | Laure Favot, 2003 [359] |
|  |  | Angiogenesis | TF, In |  | Sylvie Lamy, 2012 [14] |
|  |  | Angiogenesis | TF |  | Matsunaga Nozomu, 2010 [356] |
|  |  | Breast Cancer |  | CAM | Olga Viegas, 2019 [360] |
|  |  | Colorectal Cancer | In, Ad | Mets in mice | Chi Chou Huang, 2019 [361] |
|  |  | Lung Cancer |  | MPA in mice | Mun Hyeon Kim, 2017 [362] |
|  |  | Osteosarcoma | WH, In |  | Hae Mi Kang, 2018 [363] |
|  | Malvidin | Angiogenesis | TF |  | Matsunaga Nozomu, 2010 [356] |
| Miscellaneous  (articles with > 4 Flavonoids) |  | Angiogenesis* |  |  | Lucia Morbidelli, 2016 [364] |
|  |  | Angiogenesis* |  |  | Ladislav Mirossay, 2018 [365] |
|  |  | Angiogenesis* |  |  | Józef Dulak, 2005 [366] |
|  |  | Angiogenesis* |  |  | Carmen Diniz, 2017 [367] |
|  |  | Breast Cancer* |  |  | Andrea Kapinova, 2017 [368] |
|  |  | Cancer* |  |  | J.Mojzis, 2008 [369] |
|  |  | Cancer* |  |  | Divyashree Ravishankar, 2013 [370] |
|  |  | Cancer* |  |  | Yihai Cao, 2002 [371] |
|  |  | Cancer* |  |  | Loïc Le Marchand, 2002 [372] |
|  |  | Cancer* |  |  | Vijayalakshmi Nandakumar, 2008 [373] |
|  |  | Cancer* |  |  | Maria Angeles Martin, 2013 [374] |
|  |  | Cancer* |  |  | Jian Ping Zhang, 2016 [375] |
|  |  | Cancer* |  |  | Mirza Aghazadeh-Attari, 2020 [376] |
|  |  | Cancer* |  |  | Nam Joo Kang, 2011 [377] |
|  |  | Cancer* |  |  | Suleman S.Hussain, 2016 [378] |
|  |  | Cancer* |  |  | Hanna Lewandowska, 2016 [379] |
|  |  | Cancer* |  |  | Ching Shu Lai, 2011 [380] |
|  |  | Cancer* |  |  | Chithan Kanadaswami, 2005 [381] |
|  |  | Cancer* |  |  | Gary David Stoner, 2008 [382] |
|  |  | Colon Cancer* |  |  | Jasleen Kaur, 2015 [383] |
|  |  | Colorectal Cancer* |  |  | João R. Araújo, 2011 [384] |
|  |  | Eye disease* |  |  | Zhihan Xu, 2017 [385] |
|  |  | Hepatic Cancer* |  |  | Dimitrios Stagos, 2012 [386] |
|  |  | Hepatic Cancer* |  |  | Estefanny Ruiz García, 2018 [387] |
|  |  | Myeloma* |  |  | F.Pojero, 2019 [388] |
|  |  | Ocular angiogenesis* |  |  | Rania S. Sulaiman, 2014 [389] |
|  |  | Angiogenesis |  | CAM | Gacche Rajesh, 2010 [390] |
|  |  | Angiogenesis |  | ZFM | In Kei Lam, 2012 [391] |
|  |  | Angiogenesis | In |  | Theodore Fotsis, 1997 [392] |
|  |  | Angiogenesis | WH |  | Chalermlat Suktap, 2018 [393] |
|  |  | Cancer |  | CAM | R. N.Gacche, 2015 [394] |
|  |  | Cancer | WH |  | Jiukai Zhang, 2014 [395] |
|  |  | Cancer |  | CAM | Rajesh N.Gacche, 2011 [396] |
|  |  | Glioma | WH |  | Balbino L.Santos, 2015 [397] |
|  |  | Glioma | In |  | Amira Ouanouki, 2017 [398] |
|  |  | Hepatic Cancer | WH, TB |  | Ning Li, 2015 [399] |
|  |  | Lung Cancer | WH |  | Chun Gu Wang, 2018 [400] |
|  |  | Lung Cancer | In, Ad | Mets in mice | Yung Chin Hsiao, 2007 [401] |

*, Review article; TB, Tube Formation; WH, Wound Healing; In, Invasion; Ad, Adhesion; Mets, Metastasis; CAM, Chick Chorioallantoic Membrane; MPA, Matrigel Plug Assay; RAR, Rat Aortic Ring; EWM, Exicion Wound Model; SF, Skin Flap; RRN, Rat Retinal Neovascularization; MAR, Mice Aortic Ring; MD, Microvessel Density; MRN, Mice Retinal Neovascularization; MCN, Mice Corneal Neovasculazrization; RbCN, Rabbit Corneal Neovascularization; ZFM, Zebra Fish Model; RCN, Rat Corneal Neovascularization; CIAM, Collagen Induced Arthritis Model; DASM, Dorsal air Sac Model; IWM, Incision Wound Model.

**References**

1. Kim, M. H. Flavonoids Inhibit VEGF/BFGF-Induced Angiogenesis in Vitro by Inhibiting the Matrix-Degrading Proteases. *J. Cell. Biochem.* **2003**, *89* (3), 529–538. https://doi.org/10.1002/jcb.10543.

2. Lu, K. H.; Chen, P. N.; Hsieh, Y. H.; Lin, C. Y.; Cheng, F. Y.; Chiu, P. C.; Chu, S. C.; Hsieh, Y. S. 3-Hydroxyflavone Inhibits Human Osteosarcoma U2OS and 143B Cells Metastasis by Affecting EMT and Repressing u-PA/MMP-2 via FAK-Src to MEK/ERK and RhoA/MLC2 Pathways and Reduces 143B Tumor Growth in Vivo. *Food Chem. Toxicol.* **2016**, *97*, 177–186. https://doi.org/10.1016/j.fct.2016.09.006.

3. Yang, S. F.; Yang, W. E.; Kuo, W. H.; Chang, H. R.; Chu, S. C.; Hsieh, Y. S. Antimetastatic Potentials of Flavones on Oral Cancer Cell via an Inhibition of Matrix-Degrading Proteases. *Arch. Oral Biol.* **2008**, *53* (3), 287–294. https://doi.org/10.1016/j.archoralbio.2007.09.001.

4. Liu, R.; Deng, X.; Peng, Y.; Feng, W.; Xiong, R.; Zou, Y. Synthesis and Biological Evaluation of Novel 5 , 6 , 7-Trimethoxy Flavonoid Salicylate Derivatives as Potential Anti-Tumor Agents. *Bioorg. Chem.* **2020**, *96* (August 2019), 103652. https://doi.org/10.1016/j.bioorg.2020.103652.

5. Sim, D. Y.; Jung, H. J. Anticancer Activity of 7,8-Dihydroxyflavone in Melanoma Cells via Downregulation of α-MSH/CAMP/MITF Pathway. *Oncol. Rep.* **2016**, *36* (1), 528–534. https://doi.org/10.3892/or.2016.4825.

6. Liu, L. Z.; Jing, Y.; Jiang, L. L.; Jiang, X. E.; Jiang, Y.; Rojanasakul, Y.; Jiang, B. H. Acacetin Inhibits VEGF Expression, Tumor Angiogenesis and Growth through AKT/HIF-1α Pathway. *Biochem. Biophys. Res. Commun.* **2011**, *413* (2), 299–305. https://doi.org/10.1016/j.bbrc.2011.08.091.

7. Bhat, T. A.; Nambiar, D.; Tailor, D.; Pal, A.; Agarwal, R.; Singh, R. P. Acacetin Inhibits in Vitro and in Vivo Angiogenesis and Downregulates Stat Signaling and VEGF Expression. *Cancer Prev. Res.* **2013**, *6* (10), 1–7. https://doi.org/10.1038/jid.2014.371.

8. Fong, Y.; Shen, K. H.; Chiang, T. A.; Shih, Y. W. Acacetin Inhibits TPA-Induced MMP-2 and u-PA Expressions of Human Lung Cancer Cells through Inactivating JNK Signaling Pathway and Reducing Binding Activities of NF-ΚB and AP-1. *J. Food Sci.* **2010**, *75* (1), 30–38. https://doi.org/10.1111/j.1750-3841.2009.01438.x.

9. Chien, S. T.; Lin, S. S.; Wang, C. K.; Lee, Y. Bin; Chen, K. S.; Fong, Y.; Shih, Y. W. Acacetin Inhibits the Invasion and Migration of Human Non-Small Cell Lung Cancer A549 Cells by Suppressing the P38α MAPK Signaling Pathway. *Mol. Cell. Biochem.* **2011**, *350* (1–2), 135–148. https://doi.org/10.1007/s11010-010-0692-2.

10. Shen, K. H.; Hung, S. H.; Yin, L. Te; Huang, C. S.; Chao, C. H.; Liu, C. L.; Shih, Y. W. Acacetin, a Flavonoid, Inhibits the Invasion and Migration of Human Prostate Cancer DU145 Cells via Inactivation of the P38 MAPK Signaling Pathway. *Mol. Cell. Biochem.* **2010**, *333* (1–2), 279–291. https://doi.org/10.1007/s11010-009-0229-8.

11. Kashyap, D.; Sharma, A.; Singh, H.; Sak, K.; Kumar, V.; Singh, H.; Setzer, W. N.; Sethi, G. Apigenin : A Natural Bioactive Flavone-Type Molecule with Promising Therapeutic Function. **2018**, *48* (April), 457–471. https://doi.org/10.1016/j.jff.2018.07.037.

12. Patel, D.; Shukla, S.; Gupta, S. Apigenin and Cancer Chemoprevention: Progress, Potential and Promise (Review). *Int. J. Oncol.* **2007**, *30* (1), 233–245. https://doi.org/10.3892/ijo.30.1.233.

13. Lefort, É. C.; Blay, J. Apigenin and Its Impact on Gastrointestinal Cancers. *Mol. Nutr. Food Res.* **2013**, *57* (1), 126–144. https://doi.org/10.1002/mnfr.201200424.

14. Lamy, S.; Akla, N.; Ouanouki, A.; Lord-Dufour, S.; Béliveau, R. Diet-Derived Polyphenols Inhibit Angiogenesis by Modulating the Interleukin-6/STAT3 Pathway. *Exp. Cell Res.* **2012**, *318* (13), 1586–1596. https://doi.org/10.1016/j.yexcr.2012.04.004.

15. F. Lindenmeyer, H. Li, S. Menashi, C. Soria, and H. L. Apigenin Acts on the Tumor Cell Invasion Process and Regulates Protease Production. *Nutr. Cancer* **2001**, *39* (1), 139–147. https://doi.org/10.1207/S15327914nc391_19.

16. Lee, W. J.; Chen, W. K.; Wang, C. J.; Lin, W. L.; Tseng, T. H. Apigenin Inhibits HGF-Promoted Invasive Growth and Metastasis Involving Blocking PI3K/Akt Pathway and Β4 Integrin Function in MDA-MB-231 Breast Cancer Cells. *Toxicol. Appl. Pharmacol.* **2008**, *226* (2), 178–191. https://doi.org/10.1016/j.taap.2007.09.013.

17. Fang, J.; Zhou, Q.; Liu, L. Z.; Xia, C.; Hu, X.; Shi, X.; Jiang, B. H. Apigenin Inhibits Tumor Angiogenesis through Decreasing HIF-1α and VEGF Expression. *Carcinogenesis* **2007**, *28* (4), 858–864. https://doi.org/10.1093/carcin/bgl205.

18. Wang, L.; Kuang, L.; Hitron, J. A.; Son, Y. O.; Wang, X.; Budhraja, A.; Lee, J. C.; Pratheeshkumar, P.; Chen, G.; Zhang, Z.; Luo, J.; Shi, X. Apigenin Suppresses Migration and Invasion of Transformed Cells through Down-Regulation of C-X-C Chemokine Receptor 4 Expression. *Toxicol. Appl. Pharmacol.* **2013**, *272* (1), 108–116. https://doi.org/10.1016/j.taap.2013.05.028.

19. Spoerlein, C.; Mahal, K.; Schmidt, H.; Schobert, R. Effects of Chrysin, Apigenin, Genistein and Their Homoleptic Copper(II) Complexes on the Growth and Metastatic Potential of Cancer Cells. *J. Inorg. Biochem.* **2013**, *127*, 107–115. https://doi.org/10.1016/j.jinorgbio.2013.07.038.

20. Czyz, J.; Madeja, Z.; Irmer, U.; Korohoda, W.; Hülser, D. F. Flavonoid Apigenin Inhibits Motility and Invasiveness of Carcinoma Cells in Vitro. *Int. J. Cancer* **2005**, *114* (1), 12–18. https://doi.org/10.1002/ijc.20620.

21. Lim, W.; Park, S.; Bazer, F. W.; Song, G. Apigenin Reduces Survival of Choriocarcinoma Cells by Inducing Apoptosis via the PI3K/AKT and ERK1/2 MAPK Pathways. *J. Cell. Physiol.* **2016**, *231* (12), 2690–2699. https://doi.org/10.1002/jcp.25372.

22. Chunhua, L.; Donglan, L.; Xiuqiong, F.; Lihua, Z.; Qin, F.; Yawei, L.; Liang, Z.; Ge, W.; Linlin, J.; Ping, Z.; Kun, L.; Xuegang, S. Apigenin Up-Regulates Transgelin and Inhibits Invasion and Migration of Colorectal Cancer through Decreased Phosphorylation of AKT. *J. Nutr. Biochem.* **2013**, *24* (10), 1766–1775. https://doi.org/10.1016/j.jnutbio.2013.03.006.

23. Dai, J.; Van Wie, P. G.; Fai, L. Y.; Kim, D.; Wang, L.; Poyil, P.; Luo, J.; Zhang, Z. Downregulation of NEDD9 by Apigenin Suppresses Migration, Invasion, and Metastasis of Colorectal Cancer Cells. *Toxicol. Appl. Pharmacol.* **2016**, *311*, 106–112. https://doi.org/10.1016/j.taap.2016.09.016.

24. Coelho, P. L. C.; Oliveira, M. N.; Da Silva, A. B.; Pitanga, B. P. S.; Silva, V. D. A.; Faria, G. P.; Sampaio, G. P.; Costa, M. D. F. D.; Braga-De-Souza, S.; Costa, S. L. The Flavonoid Apigenin from Croton Betulaster Mull Inhibits Proliferation, Induces Differentiation and Regulates the Inflammatory Profile of Glioma Cells. *Anticancer. Drugs* **2016**, *27* (10), 960–969. https://doi.org/10.1097/CAD.0000000000000413.

25. Coelho, P. L. C.; Amparo, J. A. O.; da Silva, A. B.; da Silva, K. C.; Braga-de-Souza, S.; Barbosa, P. R.; Lopes, G. P. d. F.; Costa, S. L. Apigenin from Croton Betulaster Müll Restores the Immune Profile of Microglia against Glioma Cells. *Phyther. Res.* **2019**, *33* (12), 3191–3202. https://doi.org/10.1002/ptr.6491.

26. Liu, L. Z.; Fang, J.; Zhou, Q.; Hu, X.; Shi, X.; Jiang, B. H. Apigenin Inhibits Expression of Vascular Endothelial Growth Factor and Angiogenesis in Human Lung Cancer Cells: Implication of Chemoprevention of Lung Cancer. *Mol. Pharmacol.* **2005**, *68* (3), 635–643. https://doi.org/10.1124/mol.105.011254.

27. Hasnat, M. A.; Pervin, M.; Lim, J. H.; Lim, B. O. Apigenin Attenuates Melanoma Cell Migration by Inducing Anoikis through Integrin and Focal Adhesion Kinase Inhibition. *Molecules* **2015**, *20* (12), 21157–21166. https://doi.org/10.3390/molecules201219752.

28. Zhao, G.; Han, X.; Cheng, W.; Ni, J.; Zhang, Y.; Lin, J.; Song, Z. Apigenin Inhibits Proliferation and Invasion, and Induces Apoptosis and Cell Cycle Arrest in Human Melanoma Cells. *Oncol. Rep.* **2017**, *37* (4), 2277–2285. https://doi.org/10.3892/or.2017.5450.

29. Cao, H. H.; Chu, J. H.; Kwan, H. Y.; Su, T.; Yu, H.; Cheng, C. Y.; Fu, X. Q.; Guo, H.; Li, T.; Tse, A. K. W.; Chou, G. X.; Mo, H. B.; Yu, Z. L. Inhibition of the STAT3 Signaling Pathway Contributes to Apigenin-Mediated Anti-Metastatic Effect in Melanoma. *Sci. Rep.* **2016**, *6* (1), 1–12. https://doi.org/10.1038/srep21731.

30. Ghițu, A.; Schwiebs, A.; Radeke, H. H.; Avram, S.; Zupko, I.; Bor, A.; Pavel, I. Z.; Dehelean, C. A.; Oprean, C.; Bojin, F.; Farcas, C.; Soica, C.; Duicu, O.; Danciu, C. A Comprehensive Assessment of Apigenin as an Antiproliferative, Proapoptotic, Antiangiogenic and Immunomodulatory Phytocompound. *Nutrients* **2019**, *11* (4), 858–877. https://doi.org/10.3390/nu11040858.

31. Fang, J.; Xia, C.; Cao, Z.; Zheng, J. Z.; Reed, E.; Jiang, B.-H. Apigenin Inhibits VEGF and HIF‐1 Expression via PI3K/AKT/P70S6K1 and HDM2/P53 Pathways. *FASEB J.* **2005**, *19* (3), 342–353. https://doi.org/10.1096/fj.04-2175com.

32. Hu, X. M.; Meng, D.; Fang, J. Apigenin Inhibited Migration and Invasion of Human Ovarian Cancer A2780 Cells through Focal Adhesion Kinase. *Carcinogenesis* **2008**, *29* (12), 2369–2376. https://doi.org/10.1093/carcin/bgn244.

33. He, J.; Ning, C.; Wang, Y.; Ma, T.; Huang, H.; Ge, Y.; Liu, J.; Jiang, Y. Natural Plant Flavonoid Apigenin Directly Disrupts Hsp90/Cdc37 Complex and Inhibits Pancreatic Cancer Cell Growth and Migration. *J. Funct. Foods* **2015**, *18*, 10–21. https://doi.org/10.1016/j.jff.2015.06.052.

34. Erdogan, S.; Doganlar, O.; Doganlar, Z. B.; Serttas, R.; Turkekul, K.; Dibirdik, I.; Bilir, A. The Flavonoid Apigenin Reduces Prostate Cancer CD44+ Stem Cell Survival and Migration through PI3K/Akt/NF-ΚB Signaling. *Life Sci.* **2016**, *162*, 77–86. https://doi.org/10.1016/j.lfs.2016.08.019.

35. Chien, M. H.; Lin, Y. W.; Wen, Y. C.; Yang, Y. C.; Hsiao, M.; Chang, J. L.; Huang, H. C.; Lee, W. J. Targeting the SPOCK1-Snail/Slug Axis-Mediated Epithelial-to-Mesenchymal Transition by Apigenin Contributes to Repression of Prostate Cancer Metastasis. *J. Exp. Clin. Cancer Res.* **2019**, *38* (1), 1–17. https://doi.org/10.1186/s13046-019-1247-3.

36. Franzen, C. A.; Amargo, E.; Todorović, V.; Desai, B. V.; Huda, S.; Mirzoeva, S.; Chiu, K.; Grzybowski, B. A.; Chew, T. L.; Green, K. J.; Pelling, J. C. The Chemopreventive Bioflavonoid Apigenin Inhibits Prostate Cancer Cell Motility through the Focal Adhesion Kinase/Src Signaling Mechanism. *Cancer Prev. Res.* **2009**, *2* (9), 830–841. https://doi.org/10.1158/1940-6207.CAPR-09-0066.

37. Shukla, S.; Kanwal, R.; Shankar, E.; Datt, M.; Chance, M. R.; Fu, P.; MacLennan, G. T.; Gupta, S. Apigenin Blocks IKKa Activation and Suppresses Prostate Cancer Progression. *Oncotarget* **2015**, *6* (31), 31216–31232. https://doi.org/10.18632/oncotarget.5157.

38. Camero, C. M.; Germanò, M. P.; Rapisarda, A.; D’Angelo, V.; Amira, S.; Benchikh, F.; Braca, A.; De Leo, M. Anti-Angiogenic Activity of Iridoids from Galium Tunetanum. *Brazilian J. Pharmacogn.* **2018**, *28* (3), 374–377. https://doi.org/10.1016/j.bjp.2018.03.010.

39. Yeh, C. J.; Chen, C. C.; Leu, Y. L.; Lin, M. W.; Chiu, M. M.; Wang, S. H. The Effects of Artocarpin on Wound Healing: In Vitro and in Vivo Studies. *Sci. Rep.* **2017**, *7* (1), 1–13. https://doi.org/10.1038/s41598-017-15876-7.

40. Gao, Y.; Snyder, S. A.; Jaclyn N. Smith; Chen, Y. C. Anticancer Properties of Baicalein: A Review. *Med. Chem. Res.* **2016**, *25* (8), 1515–1523. https://doi.org/10.1016/j.physbeh.2017.03.040.

41. Ling, Y.; Chen, Y.; Chen, P.; Hui, H.; Song, X.; Lu, Z.; Li, C.; Lu, N.; Guo, Q. Baicalein Potently Suppresses Angiogenesis Induced by Vascular Endothelial Growth Factor through the P53/Rb Signaling Pathway Leading to G1/s Cell Cycle Arrest. *Exp. Biol. Med.* **2011**, *236* (7), 851–858. https://doi.org/10.1258/ebm.2011.010395.

42. Ma, X. C.; Yan, W.; Dai, Z.; Gao, X.; Ma, Y.; Xu, Q.; Jiang, J.; Zhang, S. Baicalein Suppresses Metastasis of Breast Cancer Cells by Inhibiting EMT via Downregulation of SATB1 and Wnt/β-Catenin Pathway. *Drug Des. Devel. Ther.* **2016**, *10*, 1419–1441. https://doi.org/10.2147/DDDT.S102541.

43. Wang, L.; Ling, Y.; Chen, Y.; Li, C. L.; Feng, F.; You, Q. D.; Lu, N.; Guo, Q. L. Flavonoid Baicalein Suppresses Adhesion, Migration and Invasion of MDA-MB-231 Human Breast Cancer Cells. *Cancer Lett.* **2010**, *297* (1), 42–48. https://doi.org/10.1016/j.canlet.2010.04.022.

44. Shang, D.; Li, Z.; Zhu, Z.; Chen, H.; Zhao, L.; Wang, X.; Chen, Y. Baicalein Suppresses 17-β-Estradiol-Induced Migration, Adhesion and Invasion of Breast Cancer Cells via the G Protein-Coupled Receptor 30 Signaling Pathway. *Oncol. Rep.* **2015**, *33* (4), 2077–2085. https://doi.org/10.3892/or.2015.3786.

45. Chung, H.; Choi, H. S.; Seo, E. K.; Kang, D. H.; Oh, E. S. Baicalin and Baicalein Inhibit Transforming Growth Factor-Β1-Mediated Epithelial-Mesenchymal Transition in Human Breast Epithelial Cells. *Biochem. Biophys. Res. Commun.* **2015**, *458* (3), 707–713. https://doi.org/10.1016/j.bbrc.2015.02.032.

46. Chai, Y.; Xu, J.; Yan, B. The Anti-Metastatic Effect of Baicalein on Colorectal Cancer. *Oncol. Rep.* **2017**, *37* (4), 2317–2323. https://doi.org/10.3892/or.2017.5437.

47. Lalou, C.; Basak, A.; Mishra, P.; Mohanta, B. C.; Banik, R.; Dinda, B.; Khatib, A. M. Inhibition of Tumor Cells Proliferation and Migration by the Flavonoid Furin Inhibitor Isolated From Oroxylum Indicum. *Curr. Med. Chem.* **2013**, *20* (4), 583–591. https://doi.org/10.2174/0929867311320040010.

48. Chen, F.; Zhuang, M.; Peng, J.; Wang, X.; Huang, T.; Li, S.; Lin, M.; Lin, H.; Xu, Y.; Li, J.; Chen, Z.; Huang, Y. Baicalein Inhibits Migration and Invasion of Gastric Cancer Cells through Suppression of the TGF-β Signaling Pathway. *Mol. Med. Rep.* **2014**, *10* (4), 1999–2003. https://doi.org/10.3892/mmr.2014.2452.

49. Chiu, W. T.; Shen, S. C.; Chow, J. M.; Lin, C. W.; Shia, L. T.; Chen, Y. C. Contribution of Reactive Oxygen Species to Migration/Invasion of Human Glioblastoma Cells U87 via ERK-Dependent COX-2/PGE2 Activation. *Neurobiol. Dis.* **2010**, *37* (1), 118–129. https://doi.org/10.1016/j.nbd.2009.09.015.

50. Chiu, Y. W.; Lin, T. H.; Huang, W. S.; Teng, C. Y.; Liou, Y. S.; Kuo, W. H.; Lin, W. L.; Huang, H. I.; Tung, J. N.; Huang, C. Y.; Liu, J. Y.; Wang, W. H.; Hwang, J. M.; Kuo, H. C. Baicalein Inhibits the Migration and Invasive Properties of Human Hepatoma Cells. *Toxicol. Appl. Pharmacol.* **2011**, *255* (3), 316–326. https://doi.org/10.1016/j.taap.2011.07.008.

51. Yu, X.; Tang, W.; Yang, Y.; Tang, L.; Dai, R.; Pu, B.; Feng, C.; Xia, J. Long Noncoding RNA NKILA Enhances the Anti-Cancer Effects of Baicalein in Hepatocellular Carcinoma via the Regulation of NF-ΚB Signaling. *Chem. Biol. Interact.* **2018**, *285*, 48–58. https://doi.org/10.1016/j.cbi.2018.02.027.

52. Park, Y. G.; Choi, J.; Jung, H. K.; Kim, B.; Kim, C.; Park, S. Y.; Seol, J. W. Baicalein Inhibits Tumor Progression by Inhibiting Tumor Cell Growth and Tumor Angiogenesis. *Oncol. Rep.* **2017**, *38* (5), 3011–3018. https://doi.org/10.3892/or.2017.6007.

53. Choi, E. O.; Cho, E. J.; Jeong, J. W.; Park, C.; Hong, S. H.; Hwang, H. J.; Moon, S. K.; Son, C. G.; Kim, W. J.; Choi, Y. H. Baicalein Inhibits the Migration and Invasion of B16F10 Mouse Melanoma Cells through Inactivation of the Pi3K/Akt Signaling Pathway. *Biomol. Ther.* **2017**, *25* (2), 213–221. https://doi.org/10.4062/biomolther.2016.094.

54. Zhang, J.; Yang, W.; Zhou, Y. B.; Xiang, Y. X.; Wang, L. S.; Hu, W. K.; Wang, W. J. Baicalein Inhibits Osteosarcoma Cell Proliferation and Invasion through the MiR-183/Ezrin Pathway. *Mol. Med. Rep.* **2018**, *18* (1), 1104–1112. https://doi.org/10.3892/mmr.2018.9036.

55. Zhang, Y.; Song, L.; Cai, L.; Wei, R.; Hu, H.; Jin, W. Effects of Baicalein on Apoptosis, Cell Cycle Arrest, Migration and Invasion of Osteosarcoma Cells. *Food Chem. Toxicol.* **2013**, *53*, 325–333. https://doi.org/10.1016/j.fct.2012.12.019.

56. Zhou, R. T.; He, M.; Yu, Z.; Liang, Y.; Nie, Y.; Tai, S.; Teng, C. B. Baicalein Inhibits Pancreatic Cancer Cell Proliferation and Invasion via Suppression of NEDD9 Expression and Its Downstream Akt and ERK Signaling Pathways. *Oncotarget* **2017**, *8* (34), 56351–56363. https://doi.org/10.18632/oncotarget.16912.

57. Lin, R.; Lin, J.; Li, S.; Ding, J.; Wu, H.; Xiang, G.; Li, S.; Huang, Y.; Lin, D.; Gao, W.; Kong, J.; Xu, H.; Zhou, K. Effects of the Traditional Chinese Medicine Baicalein on the Viability of Random Pattern Skin Flaps in Rats. *Drug Des. Devel. Ther.* **2018**, *12*, 2267–2276. https://doi.org/10.2147/DDDT.S173371.

58. Zhou, T.; Zhang, A.; Kuang, G.; Gong, X.; Jiang, R.; Lin, D.; Li, J.; Li, H.; Zhang, X.; Wan, J.; Li, H. Baicalin Inhibits the Metastasis of Highly Aggressive Breast Cancer Cells by Reversing Epithelial-to-Mesenchymal Transition by Targeting β-Catenin Signaling. *Oncol. Rep.* **2017**, *38* (6), 3599–3607. https://doi.org/10.3892/or.2017.6011.

59. Yang, B.; Bai, H.; Sa, Y.; Zhu, P.; Liu, P. Inhibiting EMT, Stemness and Cell Cycle Involved in Baicalin-Induced Growth Inhibition and Apoptosis in Colorectal Cancer Cells. *J. Cancer* **2020**, *11* (8), 2303–2317. https://doi.org/10.7150/jca.37242.

60. Zhu, Y.; Fang, J.; Wang, H.; Fei, M.; Tang, T.; Liu, K.; Niu, W.; Zhou, Y. Baicalin Suppresses Proliferation, Migration, and Invasion in Human Glioblastoma Cells via Ca 2+ -Dependent Pathway. *Drug Des. Devel. Ther.* **2018**, *12*, 3247–3261. https://doi.org/10.2147/DDDT.S176403.

61. Wu, X.; Zhi, F.; Lun, W.; Deng, Q.; Zhang, W. Baicalin Inhibits PDGF-BB-Induced Hepatic Stellate Cell Proliferation, Apoptosis, Invasion, Migration and Activation via the MiR-3595/ACSL4 Axis. *Int. J. Mol. Med.* **2018**, *41* (4), 1992–2002. https://doi.org/10.3892/ijmm.2018.3427.

62. You, J.; Cheng, J.; Yu, B.; Duan, C.; Peng, J. Baicalin, a Chinese Herbal Medicine, Inhibits the Proliferation and Migration of Human Non-Small Cell Lung Carcinoma (NSCLC) Cells, A549 and H1299, by Activating the SIRT1/AMPK Signaling Pathway. *Med. Sci. Monit.* **2018**, *24*, 2126–2133. https://doi.org/10.12659/MSM.909627.

63. Gao, C.; Zhou, Y.; Li, H.; Cong, X.; Jiang, Z.; Wang, X.; Cao, R.; Tian, W. Antitumor Effects of Baicalin on Ovarian Cancer Cells through Induction of Cell Apoptosis and Inhibition of Cell Migration in Vitro. *Mol. Med. Rep.* **2017**, *16* (6), 8729–8734. https://doi.org/10.3892/mmr.2017.7757.

64. Jo, H.; Jung, S. H.; Yim, H. Bin; Lee, S. J.; Kang, K. D. The Effect of Baicalin in a Mouse Model of Retinopathy of Prematurity. *BMB Rep.* **2015**, *48* (5), 271–276. https://doi.org/10.5483/BMBRep.2015.48.5.131.

65. Kasala, E. R.; Bodduluru, L. N.; Madana, R. M.; Athira, K. V.; Gogoi, R.; Barua, C. C. Chemopreventive and Therapeutic Potential of Chrysin in Cancer: Mechanistic Perspectives. *Toxicol. Lett.* **2015**, *233* (2), 214–225. https://doi.org/10.1016/j.toxlet.2015.01.008.

66. Tian, S.; Jiang, F.; Zhang, K.; Zhu, X.; Jin, B.; Lu, J.; Ding, Z. Fitoterapia Flavonoids from the Leaves of Carya Cathayensis Sarg . Inhibit Vascular Endothelial Growth Factor-Induced Angiogenesis. *Fitoterapia* **2014**, *92*, 34–40. https://doi.org/10.1016/j.fitote.2013.09.016.

67. Yang, B.; Huang, J.; Xiang, T.; Yin, X.; Luo, X.; Huang, J.; Luo, F.; Li, H.; Li, H.; Ren, G. Chrysin Inhibits Metastatic Potential of Human Triple-Negative Breast Cancer Cells by Modulating Matrix Metalloproteinase-10, Epithelial to Mesenchymal Transition, and PI3K/Akt Signaling Pathway. *J. Appl. Toxicol.* **2014**, *34* (1), 105–112. https://doi.org/10.1002/jat.2941.

68. Zhao, X.; Shu, G.; Chen, L.; Mi, X.; Mei, Z.; Deng, X. A Flavonoid Component from Docynia Delavayi (Franch.) Schneid Represses Transplanted H22 Hepatoma Growth and Exhibits Low Toxic Effect on Tumor-Bearing Mice. *Food Chem. Toxicol.* **2012**, *50* (9), 3166–3173. https://doi.org/10.1016/j.fct.2012.05.039.

69. Xia, Y.; Lian, S.; Khoi, P. N.; Yoon, H. J.; Han, J. Y.; Chay, K. O.; Kim, K. K.; Jung, Y. Do. Chrysin Inhibits Cell Invasion by Inhibition of Recepteur d’origine Nantais via Suppressing Early Growth Response-1 and NF-ΚB Transcription Factor Activities in Gastric Cancer Cells. *Int. J. Oncol.* **2015**, *46* (4), 1835–1843. https://doi.org/10.3892/ijo.2015.2847.

70. Wu, T. C.; Chan, S. T.; Chang, C. N.; Yu, P. S.; Chuang, C. H.; Yeh, S. L. Quercetin and Chrysin Inhibit Nickel-Induced Invasion and Migration by Downregulation of TLR4/NF-ΚB Signaling in A549 cells. *Chem. Biol. Interact.* **2018**, *292* (110), 101–109. https://doi.org/10.1016/j.cbi.2018.07.010.

71. Fu, B.; Xue, J.; Li, Z.; Shi, X.; Jiang, B. H.; Fang, J. Chrysin Inhibits Expression of Hypoxia-Inducible Factor 1-α through Reducing Hypoxia-Inducible Factor-1α Stability and Inhibiting Its Protein Synthesis. *Mol. Cancer Ther.* **2007**, *6* (1), 220–226. https://doi.org/10.1158/1535-7163.MCT-06-0526.

72. Kang, M. K.; Park, S. H.; Kim, Y. H.; Lee, E. J.; Antika, L. D.; Kim, D. Y.; Choi, Y. J.; Kang, Y. H. Dietary Compound Chrysin Inhibits Retinal Neovascularization with Abnormal Capillaries in Db/Db Mice. *Nutrients* **2016**, *8* (12). https://doi.org/10.3390/nu8120782.

73. Amrutha, K.; Nanjan, P.; Shaji, S. K.; Sunilkumar, D.; Subhalakshmi, K.; Rajakrishna, L.; Banerji, A. Discovery of Lesser Known Flavones as Inhibitors of NF-ΚB Signaling in MDA-MB-231 Breast Cancer Cells - A SAR Study. *Bioorganic Med. Chem. Lett.* **2014**, *24* (19), 4735–4742. https://doi.org/10.1016/j.bmcl.2014.07.093.

74. Prasad, P.; Vasas, A.; Hohmann, J.; Bishayee, A.; Sinha, D. Cirsiliol Suppressed Epithelial to Mesenchymal Transition in B16F10 Malignant Melanoma Cells through Alteration of the PI3K/Akt/NF-Κb Signaling Pathway. *Int. J. Mol. Sci.* **2019**, *20* (3), 19. https://doi.org/10.3390/ijms20030608.

75. Shukla, K.; Sonowal, H.; Saxena, A.; Ramana, K. V. Didymin by Suppressing NF-ΚB Activation Prevents VEGF-Induced Angiogenesis in Vitro and in Vivo. *Vascul. Pharmacol.* **2019**, *115*, 18–25. https://doi.org/10.1016/j.vph.2019.01.002.

76. Singhal, J.; Nagaprashantha, L. D.; Vatsyayan, R.; Ashutosh; Awasthi, S.; Singhal, S. S. Didymin Induces Apoptosis by Inhibiting N-Myc and Upregulating RKIP in Neuroblastoma. *Cancer Prev. Res.* **2012**, *5* (3), 473–483. https://doi.org/10.1158/1940-6207.CAPR-11-0318.

77. Yan, Y.; Liu, X.; Gao, J.; Wu, Y.; Li, Y. Inhibition of TGF-α Signaling in Gliomas by the Flavonoid Diosmetin Isolated from Dracocephalum Peregrinum L. *Molecules* **2020**, *25* (1), 192–204. https://doi.org/10.3390/molecules25010192.

78. Ge, A.; Ma, Y.; Liu, Y. N.; Li, Y. S.; Gu, H.; Zhang, J. X.; Wang, Q. X.; Zeng, X. N.; Huang, M. Diosmetin Prevents TGF-Β1-Induced Epithelial-Mesenchymal Transition via ROS/MAPK Signaling Pathways. *Life Sci.* **2016**, *153*, 1–8. https://doi.org/10.1016/j.lfs.2016.04.023.

79. Choi, J.; Lee, D.; Park, S.; Seol, J. Diosmetin Inhibits Tumor Development and Block Tumor Angiogenesis in Skin Cancer. *Biomed. Pharmacother.* **2019**, *117* (March), 109091. https://doi.org/10.1016/j.biopha.2019.109091.

80. Jiang, H.; Wu, D.; Xu, D.; Yu, H.; Zhao, Z.; Ma, D.; Jin, J. Eupafolin Exhibits Potent Anti-Angiogenic and Antitumor Activity in Hepatocellular Carcinoma. *Int. J. Biol. Sci.* **2017**, *13* (6), 701–711. https://doi.org/10.7150/ijbs.17534.

81. Cheong, J. H.; Hong, S. Y.; Zheng, Y.; Noh, S. H. Eupatilin Inhibits Gastric Cancer Cell Growth by Blocking STAT3-Mediated VEGF Expression. *J. Gastric Cancer* **2011**, *11* (1), 16–22. https://doi.org/10.5230/jgc.2011.11.1.16.

82. Fei, X.; Wang, J.; Chen, C.; Ding, B.; Fu, X.; Chen, W.; Wang, C.; Xu, R. Eupatilin Inhibits Glioma Proliferation, Migration, and Invasion by Arresting Cell Cycle at G1/S Phase and Disrupting the Cytoskeletal Structure. *Cancer Manag. Res.* **2019**, *11*, 4781–4796. https://doi.org/10.2147/CMAR.S207257.

83. Dolečková, I.; Rárová, L.; Grúz, J.; Vondrusová, M.; Strnad, M.; Kryštof, V. Antiproliferative and Antiangiogenic Effects of Flavone Eupatorin, an Active Constituent of Chloroform Extract of Orthosiphon Stamineus Leaves. *Fitoterapia* **2012**, *83* (6), 1000–1007. https://doi.org/10.1016/j.fitote.2012.06.002.

84. Du, Y.; Feng, J.; Wang, R.; Zhang, H.; Liu, J. Effects of Flavonoids from Potamogeton Crispus L. On Proliferation, Migration, and Invasion of Human Ovarian Cancer Cells. *PLoS One* **2015**, *10* (6), 1–17. https://doi.org/10.1371/journal.pone.0130685.

85. Dogan Turacli, I.; Demirtas Korkmaz, F.; Candar, T.; Ekmekci, A. Flavopiridol’s Effects on Metastasis in KRAS Mutant Lung Adenocarcinoma Cells. *J. Cell. Biochem.* **2019**, *120* (4), 5628–5635. https://doi.org/10.1002/jcb.27846.

86. Li, L.; Chen, P.; Ling, Y.; Song, X.; Lu, Z.; He, Q.; Li, Z.; Lu, N.; Guo, Q. Inhibitory Effects of GL-V9 on the Invasion of Human Breast Carcinoma Cells by Downregulating the Expression and Activity of Matrix Metalloproteinase-2/9. *Eur. J. Pharm. Sci.* **2011**, *43* (5), 393–399. https://doi.org/10.1016/j.ejps.2011.06.001.

87. Xie, J.; Gao, H.; Peng, J.; Han, Y.; Chen, X.; Jiang, Q.; Wang, C. Hispidulin Prevents Hypoxia-Induced Epithelial-Mesenchymal Transition in Human Colon Carcinoma Cells. *Am. J. Cancer Res.* **2015**, *5* (3), 1047–1061.

88. Han, M.; Gao, H.; Ju, P.; Gao, M. quan; Yuan, Y. ping; Chen, X. hong; Liu, K. li; Han, Y. tao; Han, Z. wu. Hispidulin Inhibits Hepatocellular Carcinoma Growth and Metastasis through AMPK and ERK Signaling Mediated Activation of PPARγ. *Biomed. Pharmacother.* **2018**, *103*, 272–283. https://doi.org/10.1016/j.biopha.2018.04.014.

89. He, L.; Wu, Y.; Lin, L.; Wang, J.; Wu, Y.; Chen, Y.; Yi, Z.; Liu, M.; Pang, X. Hispidulin, a Small Flavonoid Molecule, Suppresses the Angiogenesis and Growth of Human Pancreatic Cancer by Targeting Vascular Endothelial Growth Factor Receptor 2-Mediated PI3K/Akt/MTOR Signaling Pathway. *Cancer Sci.* **2011**, *102* (1), 219–225. https://doi.org/10.1111/j.1349-7006.2010.01778.x.

90. Gao, M. Q.; Gao, H.; Han, M.; Liu, K. L.; Peng, J. J.; Han, Y. T. Hispidulin Suppresses Tumor Growth and Metastasis in Renal Cell Carcinoma by Modulating Ceramide-Sphingosine 1-Phosphate Rheostat. *Am. J. Cancer Res.* **2017**, *7* (7), 1501–1514.

91. Li, C.; Li, F.; Zhao, K.; Yao, J.; Cheng, Y.; Zhao, L.; Li, Z.; Lu, N.; Guo, Q. LFG-500 Inhibits the Invasion of Cancer Cells via down-Regulation of PI3K/AKT/NF-ΚB Signaling Pathway. *PLoS One* **2014**, *9* (3), 1–12. https://doi.org/10.1371/journal.pone.0091332.

92. Zhen, Z. G.; Ren, S. H.; Ji, H. M.; Ma, J. H.; Ding, X. M.; Feng, F. Q.; Chen, S. L.; Zou, P.; Ren, J. R.; Jia, L. Linarin Suppresses Glioma through Inhibition of NF-ΚB/P65 and up-Regulating P53 Expression in Vitro and in Vivo. *Biomed. Pharmacother.* **2017**, *95*, 363–374. https://doi.org/10.1016/j.biopha.2017.08.023.

93. Cook, M. T. Mechanism of Metastasis Suppression by Luteolin in Breast Cancer. *Breast Cancer Targets Ther.* **2018**, *10*, 89–100. https://doi.org/10.2147/BCTT.S144202.

94. Li, X.; Chen, M.; Lei, X.; Huang, M.; Ye, W.; Zhang, R.; Zhang, D. Luteolin Inhibits Angiogenesis by Blocking Gas6/Axl Signaling Pathway. *Int. J. Oncol.* **2017**, *51* (2), 677–685. https://doi.org/10.3892/ijo.2017.4041.

95. Zhu, M.; Chen, D.; Li, D.; Ding, H.; Zhang, T.; Xu, T.; Zhang, Y. Luteolin Inhibits Angiotensin II-Induced Human Umbilical Vein Endothelial Cell Proliferation and Migration through Downregulation of Src and Akt Phosphorylation. *Circ. J.* **2013**, *77* (3), 772–779. https://doi.org/10.1253/circj.CJ-12-0310.

96. Ravishankar, D.; Watson, K. A.; Boateng, S. Y.; Green, R. J.; Greco, F.; Osborn, H. M. I. Exploring Quercetin and Luteolin Derivatives as Antiangiogenic Agents. *Eur. J. Med. Chem.* **2015**, *97*, 259–274. https://doi.org/10.1016/j.ejmech.2015.04.056.

97. Li, H.; Lin, D.; Kuang, G.; Wan, J.; Zhang, X.; Li, H.; Xia, G. Luteolin Suppresses the Metastasis of Triple-Negative Breast Cancer by Reversing Epithelial-to-Mesenchymal Transition via Downregulation of β-Catenin Expression. *Oncol. Rep.* **2017**, *37* (2), 895–902. https://doi.org/10.3892/or.2016.5311.

98. Naso, L. G.; Badiola, I.; Marquez Clavijo, J.; Valcarcel, M.; Salado, C.; Ferrer, E. G.; Williams, P. A. M. Inhibition of the Metastatic Progression of Breast and Colorectal Cancer in Vitro and in Vivo in Murine Model by the Oxidovanadium(IV) Complex with Luteolin. *Bioorganic Med. Chem.* **2016**, *24* (22), 6004–6011. https://doi.org/10.1016/j.bmc.2016.09.058.

99. Lee, L. T.; Huang, Y. T.; Hwang, J. J.; Lee, A. Y. L.; Ke, F. C.; Huang, C. J.; Kandaswami, C.; Lee, P. P. H.; Lee, M. T. Transinactivation of the Epidermal Growth Factor Receptor Tyrosine Kinase and Focal Adhesion Kinase Phosphorylation by Dietary Flavonoids: Effect on Invasive Potential of Human Carcinoma Cells. *Biochem. Pharmacol.* **2004**, *67* (11), 2103–2114. https://doi.org/10.1016/j.bcp.2004.02.023.

100. Attoub, S.; Hassan, A. H.; Vanhoecke, B.; Iratni, R.; Takahashi, T.; Gaben, A. M.; Bracke, M.; Awad, S.; John, A.; Kamalboor, H. A.; Al Sultan, M. A.; Arafat, K.; Gespach, C.; Petroianu, G. Inhibition of Cell Survival, Invasion, Tumor Growth and Histone Deacetylase Activity by the Dietary Flavonoid Luteolin in Human Epithelioid Cancer Cells. *Eur. J. Pharmacol.* **2011**, *651* (1–3), 18–25. https://doi.org/10.1016/j.ejphar.2010.10.063.

101. Bagli, E.; Stefaniotou, M.; Morbidelli, L.; Ziche, M.; Psillas, K.; Murphy, C.; Fotsis, T. Luteolin Inhibits Vascular Endothelial Growth Factor-Induced Angiogenesis; Inhibition of Endothelial Cell Survival and Proliferation by Targeting Phosphatidylinositol 3′-Kinase Activity. *Cancer Res.* **2004**, *64* (21), 7936–7946. https://doi.org/10.1158/0008-5472.CAN-03-3104.

102. Yao, Y.; Rao, C.; Zheng, G.; Wang, S. Luteolin Suppresses Colorectal Cancer Cell Metastasis via Regulation of the MiR-384/Pleiotrophin Axis. *Oncol. Rep.* **2019**, *42* (1), 131–141. https://doi.org/10.3892/or.2019.7136.

103. Lodhi, S.; Singhai, A. K. Wound Healing Effect of Flavonoid Rich Fraction and Luteolin Isolated from Martynia Annua Linn. on Streptozotocin Induced Diabetic Rats. *Asian Pac. J. Trop. Med.* **2013**, *6* (4), 253–259. https://doi.org/10.1016/S1995-7645(13)60053-X.

104. Lu, J.; Li, G.; He, K.; Jiang, W.; Xu, C.; Li, Z.; Wang, H.; Wang, W.; Wang, H.; Teng, X.; Teng, L. Luteolin Exerts a Marked Antitumor Effect in CMet-Overexpressing Patient-Derived Tumor Xenograft Models of Gastric Cancer. *J. Transl. Med.* **2015**, *13* (1), 1–11. https://doi.org/10.1186/s12967-015-0398-z.

105. Zang, M. de; Hu, L.; Fan, Z. yuan; Wang, H. xiao; Zhu, Z. lun; Cao, S.; Wu, X. yan; Li, J. fang; Su, L. ping; Li, C.; Zhu, Z. gang; Yan, M.; Liu, B. ya. Luteolin Suppresses Gastric Cancer Progression by Reversing Epithelial-Mesenchymal Transition via Suppression of the Notch Signaling Pathway. *J. Transl. Med.* **2017**, *15* (1), 1–11. https://doi.org/10.1186/s12967-017-1151-6.

106. Pu, Y.; Zhang, T.; Wang, J.; Mao, Z.; Duan, B.; Long, Y.; Xue, F.; Liu, D.; Liu, S.; Gao, Z. Luteolin Exerts an Anticancer Effect on Gastric Cancer Cells through Multiple Signaling Pathways and Regulating MiRNAs. *J. Cancer* **2018**, *9* (20), 3669–3675. https://doi.org/10.7150/jca.27183.

107. Wang, Q.; Wang, H.; Jia, Y.; Ding, H.; Zhang, L.; Pan, H. Luteolin Reduces Migration of Human Glioblastoma Cell Lines via Inhibition of the P-IGF-1R/PI3K/AKT/MTOR Signaling Pathway. *Oncol. Lett.* **2017**, *14* (3), 3545–3551. https://doi.org/10.3892/ol.2017.6643.

108. Selvi, R. B.; Swaminathan, A.; Chatterjee, S.; Shanmugam, M. K.; Li, F.; Ramakrishnan, G. B.; Siveen, K. S.; Chinnathambi, A.; Emam Zayed, M.; Alharbi, S. A.; Basha, J.; Bhat, A.; Vasudevan, M.; Dharmarajan, A.; Sethi, G.; Kundu, T. K. Inhibition of P300 Lysine Acetyltransferase Activity by Luteolin Reduces Tumor Growth in Head and Neck Squamous Cell Carcinoma (HNSCC) Xenograft Mouse Model. *Oncotarget* **2015**, *6* (41), 43806–43818. https://doi.org/10.18632/oncotarget.6245.

109. Cheng, W. Y.; Chiao, M. T.; Liang, Y. J.; Yang, Y. C.; Shen, C. C.; Yang, C. Y. Luteolin Inhibits Migration of Human Glioblastoma U-87 MG and T98G Cells through Downregulation of Cdc42 Expression and PI3K/AKT Activity. *Mol. Biol. Rep.* **2013**, *40* (9), 5315–5326. https://doi.org/10.1007/s11033-013-2632-1.

110. Franco, Y. E. M.; de Lima, C. A.; Rosa, M. N.; Silva, V. A. O.; Reis, R. M.; Priolli, D. G.; Carvalho, P. O.; do Nascimento, J. R.; da Rocha, C. Q.; Longato, G. B. Investigation of U-251 Cell Death Triggered by Flavonoid Luteolin: Towards a Better Understanding on Its Anticancer Property against Glioblastomas. *Nat. Prod. Res.* **2020**, 1–7. https://doi.org/10.1080/14786419.2020.1727470.

111. Lee, W. J.; Wu, L. F.; Chen, W. K.; Wang, C. J.; Tseng, T. H. Inhibitory Effect of Luteolin on Hepatocyte Growth Factor/Scatter Factor-Induced HepG2 Cell Invasion Involving Both MAPK/ERKs and PI3K-Akt Pathways. *Chem. Biol. Interact.* **2006**, *160* (2), 123–133. https://doi.org/10.1016/j.cbi.2006.01.002.

112. Meng, G.; Chai, K.; Li, X.; Zhu, Y.; Huang, W. Luteolin Exerts Pro-Apoptotic Effect and Anti-Migration Effects on A549 Lung Adenocarcinoma Cells through the Activation of MEK/ERK Signaling Pathway. *Chem. Biol. Interact.* **2016**, *257*, 26–34. https://doi.org/10.1016/j.cbi.2016.07.028.

113. Ruan, J.; Zhang, L.; Yan, L.; Liu, Y.; Yue, Z.; Chen, L.; Wang, A. Y.; Chen, W.; Zheng, S.; Wang, S.; Lu, Y. Inhibition of Hypoxia-Induced Epithelial Mesenchymal Transition by Luteolin in Non-Small Cell Lung Cancer Cells. *Mol. Med. Rep.* **2012**, *6* (1), 232–238. https://doi.org/10.3892/mmr.2012.884.

114. Lin, Y. C.; Tsai, P. H.; Lin, C. Y.; Cheng, C. H.; Lin, T. H.; Lee, K. P. H.; Huang, K. Y.; Chen, S. H.; Hwang, J. J.; Kandaswami, C. C.; Lee, M. T. Impact of Flavonoids on Matrix Metalloproteinase Secretion and Invadopodia Formation in Highly Invasive A431-III Cancer Cells. *PLoS One* **2013**, *8* (8), 71903–71915. https://doi.org/10.1371/journal.pone.0071903.

115. Li, C.; Wang, Q.; Shen, S.; Wei, X.; Li, G. HIF-1α/VEGF Signaling-Mediated Epithelial–Mesenchymal Transition and Angiogenesis Is Critically Involved in Anti-Metastasis Effect of Luteolin in Melanoma Cells. *Phyther. Res.* **2019**, *33* (3), 798–807. https://doi.org/10.1002/ptr.6273.

116. Yao, X.; Jiang, W.; Yu, D.; Yan, Z. Luteolin Inhibits Proliferation and Induces Apoptosis of Human Melanoma Cells: In Vivo and in Vitro by Suppressing MMP-2 and MMP-9 through the PI3K/AKT Pathway. *Food Funct.* **2019**, *10* (2), 703–712. https://doi.org/10.1039/c8fo02013b.

117. Ruan, J. S.; Liu, Y. P.; Zhang, L.; Yan, L. G.; Fan, F. T.; Shen, C. S.; Wang, A. Y.; Zheng, S. Z.; Wang, S. M.; Lu, Y. Luteolin Reduces the Invasive Potential of Malignant Melanoma Cells by Targeting Β3 Integrin and the Epithelial-Mesenchymal Transition. *Acta Pharmacol. Sin.* **2012**, *33* (10), 1325–1331. https://doi.org/10.1038/aps.2012.93.

118. Velmurugan, B. K.; Lin, J.-T.; Mahalakshmi, B.; Chuang, Y.-C.; Lin, C.-C.; Lo, Y.-S.; Hsieh, M.-J.; Chen, M.-K. Luteolin-7-O-Glucoside Inhibits Oral Cancer Cell Migration and Invasion by Regulating Matrix Metalloproteinase-2 Expression and Extracellular Signal-Regulated Kinase Pathway. *Biomolecules* **2020**, *10* (4), 502. https://doi.org/10.3390/biom10040502.

119. Huang, Y. T.; Lee, L. T.; Lee, P. P. H.; Lin, Y. S.; Lee, M. T. Targeting of Focal Adhesion Kinase by Flavonoids and Small-Interfering RNAs Reduces Tumor Cell Migration Ability. *Anticancer Res.* **2005**, *25* (3 B), 2017–2025.

120. Pratheeshkumar, P.; Son, Y. O.; Budhraja, A.; Wang, X.; Ding, S.; Wang, L.; Hitron, A.; Lee, J. C.; Kim, D.; Divya, S. P.; Chen, G.; Zhang, Z.; Luo, J.; Shi, X. Luteolin Inhibits Human Prostate Tumor Growth by Suppressing Vascular Endothelial Growth Factor Receptor 2-Mediated Angiogenesis. *PLoS One* **2012**, *7* (12), 52279. https://doi.org/10.1371/journal.pone.0052279.

121. Joussen, A. M. Treatment of Corneal Neovascularization with Dietary Isoflavonoids and Flavonoids. *Exp. Eye Res.* **2000**, *71* (5), 483–487. https://doi.org/10.1006/exer.2000.0900.

122. Fan, S. H.; Wang, Y. Y.; Lu, J.; Zheng, Y. L.; Wu, D. M.; Li, M. Q.; Hu, B.; Zhang, Z.; Cheng, W.; Shan, Q. Luteoloside Suppresses Proliferation and Metastasis of Hepatocellular Carcinoma Cells by Inhibition of NLRP3 Inflammasome. *PLoS One* **2014**, *9* (2), 1–11. https://doi.org/10.1371/journal.pone.0089961.

123. Zhao, K.; Yuan, Y.; Lin, B.; Miao, Z.; Li, Z.; Guo, Q.; Lu, N. LW-215 , a Newly Synthesized Flavonoid , Exhibits Potent Anti-Angiogenic Activity in Vitro and in Vivo. *Gene* **2018**, *642*, 533–541. https://doi.org/10.1016/j.gene.2017.11.065.

124. Chen, Y.; Lu, N.; Ling, Y.; Wang, L.; You, Q.; Li, Z.; Guo, Q. LYG-202, a Newly Synthesized Flavonoid, Exhibits Potent Anti-Angiogenic Activity in Vitro and in Vivo. *J. Pharmacol. Sci.* **2010**, *112* (1), 37–45. https://doi.org/10.1254/jphs.09213FP.

125. Zhao, K.; Yao, Y.; Luo, X.; Lin, B.; Huang, Y.; Zhou, Y.; Li, Z.; Guo, Q.; Lu, N. LYG-202 Inhibits Activation of Endothelial Cells and Angiogenesis through CXCL12/CXCR7 Pathway in Breast Cancer. *Carcinogenesis* **2018**, *39* (4), 588–600. https://doi.org/10.1093/carcin/bgy007.

126. Lin, W. L.; Lai, D. Y.; Lee, Y. J.; Chen, N. F.; Tseng, T. H. Antitumor Progression Potential of Morusin Suppressing STAT3 and NFκB in Human Hepatoma SK-Hep1 Cells. *Toxicol. Lett.* **2015**, *232* (2), 490–498. https://doi.org/10.1016/j.toxlet.2014.11.031.

127. Lam, K. H.; Alex, D.; Lam, I. K.; Tsui, S. K. W.; Yang, Z. F.; Lee, S. M. Y. Nobiletin, a Polymethoxylated Flavonoid from Citrus, Shows Anti-Angiogenic Activity in a Zebrafish in Vivo Model and HUVEC in Vitro Model. *J. Cell. Biochem.* **2011**, *112* (11), 3313–3321. https://doi.org/10.1002/jcb.23257.

128. Nipin, S. P.; Kang, D. Y.; Joung, Y. H.; Park, J. H.; Kim, W. S.; Lee, H. K.; Song, K. D.; Park, Y. M.; Yang, Y. M. Nobiletin Inhibits Angiogenesis by Regulating Src/FAK/STAT3-Mediated Signaling through PXN In ER+ Breast Cancer Cells. *Int. J. Mol. Sci.* **2017**, *18* (5). https://doi.org/10.3390/ijms18050935.

129. Sp, N.; Kang, D. Y.; Kim, D. H.; Park, J. H.; Lee, H. G.; Kim, H. J.; Darvin, P.; Park, Y. M.; Yang, Y. M. Nobiletin Inhibits CD36-Dependent Tumor Angiogenesis, Migration, Invasion, and Sphere Formation through the Cd36/Stat3/Nf-Kb Signaling Axis. *Nutrients* **2018**, *10* (6). https://doi.org/10.3390/nu10060772.

130. Liu, J.; Wang, S.; Tian, S.; He, Y.; Lou, H.; Yang, Z.; Kong, Y.; Cao, X. Nobiletin Inhibits Breast Cancer via P38 Mitogen-Activated Protein Kinase, Nuclear Transcription Factor-Κb, and Nuclear Factor Erythroid 2-Related Factor 2 Pathways in MCF-7 Cells. *Food Nutr. Res.* **2018**, *62* (13), 1–10. https://doi.org/10.29219/fnr.v62.1323.

131. Lee, Y. C.; Cheng, T. H.; Lee, J. S.; Chen, J. H.; Liao, Y. C.; Fong, Y.; Wu, C. H.; Shih, Y. W. Nobiletin, a Citrus Flavonoid, Suppresses Invasion and Migration Involving FAK/PI3K/Akt and Small GTPase Signals in Human Gastric Adenocarcinoma AGS Cells. *Mol. Cell. Biochem.* **2011**, *347* (1–2), 103–115. https://doi.org/10.1007/s11010-010-0618-z.

132. Lien, L. M.; Wang, M. J.; Chen, R. J.; Chiu, H. C.; Wu, J. L.; Shen, M. Y.; Chou, D. S.; Sheu, J. R.; Lin, K. H.; Lu, W. J. Nobiletin, a Polymethoxylated Flavone, Inhibits Glioma Cell Growth and Migration via Arresting Cell Cycle and Suppressing MAPK and Akt Pathways. *Phyther. Res.* **2016**, *30* (2), 214–221. https://doi.org/10.1002/ptr.5517.

133. Zhang, X.; Zheng, K.; Li, C.; Zhao, Y.; Li, H.; Liu, X.; Long, Y.; Yao, J. Nobiletin Inhibits Invasion via Inhibiting AKT/GSK3β/β-Catenin Signaling Pathway in Slug-Expressing Glioma Cells. *Oncol. Rep.* **2017**, *37* (5), 2847–2856. https://doi.org/10.3892/or.2017.5522.

134. Shi, M. Der; Liao, Y. C.; Shih, Y. W.; Tsai, L. Y. Nobiletin Attenuates Metastasis via Both ERK and PI3K/Akt Pathways in HGF-Treated Liver Cancer HepG2 Cells. *Phytomedicine* **2013**, *20* (8–9), 743–752. https://doi.org/10.1016/j.phymed.2013.02.004.

135. Da, C.; Liu, Y.; Zhan, Y.; Liu, K.; Wang, R. Nobiletin Inhibits Epithelial-Mesenchymal Transition of Human Non-Small Cell Lung Cancer Cells by Antagonizing the TGF-Β1/Smad3 Signaling Pathway. *Oncol. Rep.* **2016**, *35* (5), 2767–2774. https://doi.org/10.3892/or.2016.4661.

136. Chen, J.; Chen, A. Y.; Huang, H.; Ye, X.; Rollyson, W. D.; Perry, H. E.; Brown, K. C.; Rojanasakul, Y.; Rankin, G. O.; Dasgupta, P.; Chen, Y. C. The Flavonoid Nobiletin Inhibits Tumor Growth and Angiogenesis of Ovarian Cancers via the Akt Pathway. *Int. J. Oncol.* **2015**, *46* (6), 2629–2638. https://doi.org/10.3892/ijo.2015.2946.

137. Liu, F.; Zhang, S.; Yin, M.; Guo, L.; Xu, M.; Wang, Y. Nobiletin Inhibits Hypoxia-Induced Epithelial-Mesenchymal Transition in Renal Cell Carcinoma Cells. *J. Cell. Biochem.* **2019**, *120* (2), 2039–2046. https://doi.org/10.1002/jcb.27511.

138. Jiang, R.; Lin, C.; Jiang, C.; Huang, Z.; Gao, W.; Lin, D. Nobiletin Enhances the Survival of Random Pattern Skin Fl Aps : Involvement of Enhancing Angiogenesis and Inhibiting Oxidative Stress. *Int. Immunopharmacol.* **2020**, *78*, 106010. https://doi.org/10.1016/j.intimp.2019.106010.

139. Kim, S. J.; Pham, T. H.; Bak, Y.; Ryu, H. W.; Oh, S. R.; Yoon, D. Y. Orientin Inhibits Invasion by Suppressing MMP-9 and IL-8 Expression via the PKCα/ ERK/AP-1/STAT3-Mediated Signaling Pathways in TPA-Treated MCF-7 Breast Cancer Cells. *Phytomedicine* **2018**, *50*, 35–42. https://doi.org/10.1016/j.phymed.2018.09.172.

140. Huang, H. K.; Lee, S. Y.; Huang, S. F.; Lin, Y. S.; Chao, S. C.; Huang, S. F.; Lee, S. C.; Cheng, T. H.; Loh, S. H.; Tsai, Y. T. Isoorientin Decreases Cell Migration via Decreasing Functional Activity and Molecular Expression of Proton-Linked Monocarboxylate Transporters in Human Lung Cancer Cells. *Am. J. Chin. Med.* **2020**, *48* (1), 201–222. https://doi.org/10.1142/S0192415X20500111.

141. Lu, Z.; Lu, N.; Li, C.; Li, F.; Zhao, K.; Lin, B.; Guo, Q. Oroxylin A Inhibits Matrix Metalloproteinase-2/9 Expression and Activation by up-Regulating Tissue Inhibitor of Metalloproteinase-2 and Suppressing the ERK1/2 Signaling Pathway. *Toxicol. Lett.* **2012**, *209* (3), 211–220. https://doi.org/10.1016/j.toxlet.2011.12.022.

142. Li, Y.; Gan, C.; Zhang, Y.; Yu, Y.; Fan, C.; Deng, Y.; Zhang, Q.; Yu, X.; Zhang, Y.; Wang, L.; He, F.; Xie, Y.; Ye, T.; Yin, W. Inhibition of Stat3 Signaling Pathway by Natural Product Pectolinarigenin Attenuates Breast Cancer Metastasis. *Front. Pharmacol.* **2019**, *10*, 1–13. https://doi.org/10.3389/fphar.2019.01195.

143. Gan, C.; Li, Y.; Yu, Y.; Yu, X.; Liu, H.; Zhang, Q.; Yin, W.; Yu, L.; Ye, T. Natural Product Pectolinarigenin Exhibits Potent Anti-Metastatic Activity in Colorectal Carcinoma Cells in Vitro and in Vivo. *Bioorganic Med. Chem.* **2019**, *27* (21). https://doi.org/10.1016/j.bmc.2019.115089.

144. Liu, S.; Zhang, J.; Yang, H.; Zhang, Q.; Chen, M. Pectolinarigenin Flavonoid Exhibits Selective Anti-Proliferative Activity in Cisplatin-Resistant Hepatocellular Carcinoma, Autophagy Activation, Inhibiting Cell Migration and Invasion, G2/M Phase Cell Cycle Arrest and Targeting ERK1/2 MAP Kinases. *J. B.U.ON.* **2020**, *25* (1), 415–420.

145. Wu, X.; Yu, N.; Zhang, Y.; Ye, Y.; Sun, W.; Ye, L.; Wu, H.; Yang, Z.; Wu, L.; Wang, F. Radix Tetrastigma Hemsleyani Flavone Exhibits Antitumor Activity in Colorectal Cancer via Wnt/β-Catenin Signaling Pathway. *Onco. Targets. Ther.* **2018**, *11*, 6437–6446. https://doi.org/10.2147/OTT.S172048.

146. Gao, Z. X. Z.; Huang, D. Y.; Li, H. X.; Zhang, L. N.; Lv, Y. H.; Cui, H. D.; Zheng, J. H. Scutellarin Promotes in Vitro Angiogenesis in Human Umbilical Vein Endothelial Cells. *Biochem. Biophys. Res. Commun.* **2010**, *400* (1), 151–156. https://doi.org/10.1016/j.bbrc.2010.08.034.

147. Lv, W. L.; Liu, Q.; An, J. H.; Song, X. Y. Scutellarin Inhibits Hypoxia-Induced Epithelial-Mesenchymal Transition in Bladder Cancer Cells. *J. Cell. Physiol.* **2019**, *234* (12), 23169–23175. https://doi.org/10.1002/jcp.28883.

148. Tang, S. L.; Gao, Y. L.; Hu, W. Z. Scutellarin Inhibits the Metastasis and Cisplatin Resistance in Glioma Cells. *Onco. Targets. Ther.* **2019**, *12*, 587–598. https://doi.org/10.2147/OTT.S187426.

149. Ke, Y.; Bao, T.; Wu, X.; Tang, H.; Wang, Y.; Ge, J.; Fu, B.; Meng, X.; Chen, L.; Zhang, C.; Tan, Y.; Chen, H.; Guo, Z.; Ni, F.; Lei, X.; Shi, Z.; Wei, D.; Wang, L. Scutellarin Suppresses Migration and Invasion of Human Hepatocellular Carcinoma by Inhibiting the STAT3/Girdin/Akt Activity. *Biochem. Biophys. Res. Commun.* **2017**, *483* (1), 509–515. https://doi.org/10.1016/j.bbrc.2016.12.114.

150. Li, C. Y.; Wang, Q.; Wang, X.; Li, G.; Shen, S.; Wei, X. Scutellarin Inhibits the Invasive Potential of Malignant Melanoma Cells through the Suppression Epithelial-Mesenchymal Transition and Angiogenesis via the PI3K/Akt/MTOR Signaling Pathway. *Eur. J. Pharmacol.* **2019**, *858* (22), 172463. https://doi.org/10.1016/j.ejphar.2019.172463.

151. Deng, W.; Han, W.; Fan, T.; Wang, X.; Cheng, Z.; Wan, B.; Chen, J. Scutellarin Inhibits Human Renal Cancer Cell Proliferation and Migration via Upregulation of PTEN. *Biomed. Pharmacother.* **2018**, *107*, 1505–1513. https://doi.org/10.1016/j.biopha.2018.08.127.

152. Thirusangu, P.; Vigneshwaran, V.; Vijay Avin, B. R.; Rakesh, H.; Vikas, H. M.; Prabhakar, B. T. Scutellarein Antagonizes the Tumorigenesis by Modulating Cytokine VEGF Mediated Neoangiogenesis and DFF-40 Actuated Nucleosomal Degradation. *Biochem. Biophys. Res. Commun.* **2017**, *484* (1), 85–92. https://doi.org/10.1016/j.bbrc.2017.01.067.

153. Chung, T. Te; Chuang, C. Y.; Teng, Y. H.; Hsieh, M. J.; Lai, J. C.; Chuang, Y. T.; Chen, M. K.; Yang, S. F. Tricetin Suppresses Human Oral Cancer Cell Migration by Reducing Matrix Metalloproteinase-9 Expression through the Mitogen-Activated Protein Kinase Signaling Pathway. *Environ. Toxicol.* **2017**, *32* (11), 2392–2399. https://doi.org/10.1002/tox.22452.

154. Chang, P. Y.; Hsieh, M. J.; Hsieh, Y. S.; Chen, P. N.; Yang, J. S.; Lo, F. C.; Yang, S. F.; Lu, K. H. Tricetin Inhibits Human Osteosarcoma Cells Metastasis by Transcriptionally Repressing MMP-9 via P38 and Akt Pathways. *Environ. Toxicol.* **2017**, *32* (8), 2032–2040. https://doi.org/10.1002/tox.22380.

155. Chung, D. J.; Wang, C. J.; Yeh, C. W.; Tseng, T. H. Inhibition of the Proliferation and Invasion of C6 Glioma Cells by Tricin via the Upregulation of Focal-Adhesion-Kinase-Targeting MicroRNA-7. *J. Agric. Food Chem.* **2018**, *66* (26), 6708–6716. https://doi.org/10.1021/acs.jafc.8b00604.

156. Seki, N.; Toh, U.; Kawaguchi, K.; Ninomiya, M.; Koketsu, M.; Watanabe, K.; Aoki, M.; Fujii, T.; Nakamura, A.; Akagi, Y.; Kusukawa, J.; Kage, M.; Shirouzu, K.; Yamana, H. Tricin Inhibits Proliferation of Human Hepatic Stellate Cells in Vitro by Blocking Tyrosine Phosphorylation of PDGF Receptor and Its Signaling Pathways. *J. Cell. Biochem.* **2012**, *113* (7), 2346–2355. https://doi.org/10.1002/jcb.24107.

157. Li, F.; Li, C.; Zhang, H.; Lu, Z.; Li, Z.; You, Q.; Lu, N.; Guo, Q. VI-14, a Novel Flavonoid Derivative, Inhibits Migration and Invasion of Human Breast Cancer Cells. *Toxicol. Appl. Pharmacol.* **2012**, *261* (2), 217–226. https://doi.org/10.1016/j.taap.2012.04.011.

158. Luo, Y.; Ren, Z.; Du, B.; Xing, S.; Huang, S.; Li, Y.; Lei, Z.; Li, D.; Chen, H.; Huang, Y.; Wei, G. Structure Identification of ViceninII Extracted from Dendrobium Officinale and the Reversal of TGF-Β1-Induced Epithelial–Mesenchymal Transition in Lung Adenocarcinoma Cells through TGF-β/Smad and PI3K/Akt/MTOR Signaling Pathways. *Molecules* **2019**, *24* (1), 1–17. https://doi.org/10.3390/molecules24010144.

159. Choi, H. J.; Eun, J. S.; Kim, B. G.; Kim, S. Y.; Jeon, H.; Soh, Y. Vitexin, an HIF-1α Inhibitor, Has Anti-Metastatic Potential in PC12 Cells. *Mol. Cells* **2006**, *22* (3), 291–299.

160. Huynh, D. L.; Sharma, N.; Kumar Singh, A.; Singh Sodhi, S.; ZHANG, J. J.; Mongre, R. K.; Ghosh, M.; Kim, N.; Ho Park, Y.; Kee Jeong, D. Anti-Tumor Activity of Wogonin, an Extract from Scutellaria Baicalensis, through Regulating Different Signaling Pathways. *Chin. J. Nat. Med.* **2017**, *15* (1), 15–40. https://doi.org/10.1016/S1875-5364(17)30005-5.

161. Lin, C. M.; Chang, H.; Chen, Y. H.; Li, S. Y.; Wu, I. H.; Chiu, J. H. Protective Role of Wogonin against Lipopolysaccharide-Induced Angiogenesis via VEGFR-2, Not VEGFR-1. *Int. Immunopharmacol.* **2006**, *6* (11), 1690–1698. https://doi.org/10.1016/j.intimp.2006.07.003.

162. Song, X.; Yao, J.; Wang, F.; Zhou, M.; Zhou, Y.; Wang, H.; Wei, L.; Zhao, L.; Li, Z.; Lu, N.; Guo, Q. Wogonin Inhibits Tumor Angiogenesis via Degradation of HIF-1α Protein. *Toxicol. Appl. Pharmacol.* **2013**, *271* (2), 144–155. https://doi.org/10.1016/j.taap.2013.04.031.

163. Shen, S. C.; Lin, C. W.; Lee, H. M.; Chien, L. L.; Chen, Y. C. Lipopolysaccharide plus 12-o-Tetradecanoylphorbol 13-Acetate Induction of Migration and Invasion of Glioma Cells in Vitro and in Vivo: Differential Inhibitory Effects of Flavonoids. *Neuroscience* **2006**, *140* (2), 477–489. https://doi.org/10.1016/j.neuroscience.2006.02.028.

164. Fu, R.; Chen, Y.; Wang, X.; An, T.; Tao, L.; Zhou, Y. Wogonin Inhibits Multiple Myeloma-Stimulated Angiogenesis via c-Myc / VHL / HIF-1 a Signaling Axis. *Oncotarget* **2016**, *7* (5), 5715–5727.

165. Chen, Y.; Lu, N.; Ling, Y.; Gao, Y.; Wang, L.; Sun, Y.; Qi, Q.; Feng, F.; Liu, W.; Liu, W.; You, Q.; Guo, Q. Wogonoside Inhibits Lipopolysaccharide-Induced Angiogenesis in Vitro and in Vivo via Toll-like Receptor 4 Signal Transduction. *Toxicology* **2009**, *259* (1–2), 10–17. https://doi.org/10.1016/j.tox.2009.01.010.

166. Huang, Y.; Zhao, K.; Hu, Y.; Zhou, Y.; Luo, X.; Li, X.; Wei, L.; Li, Z.; You, Q.; Guo, Q.; Lu, N. Wogonoside Inhibits Angiogenesis in Breast Cancer via Suppressing Wnt/β-Catenin Pathway. *Mol. Carcinog.* **2016**, *55* (11), 1598–1612. https://doi.org/10.1002/mc.22412.

167. Chen, S.; Wu, Z.; Ke, Y.; Shu, P.; Chen, C.; Lin, R.; Shi, Q. Wogonoside Inhibits Tumor Growth and Metastasis in Endometrial Cancer via ER Stress-Hippo Signaling Axis. *Acta Biochim. Biophys. Sin. (Shanghai).* **2019**, *51* (11), 1096–1105. https://doi.org/10.1093/abbs/gmz109.

168. Chen, Y.; Cheng, Y.; Hung, A. C.; Wu, Y.; Hou, M. The Synthetic Flavonoid WYC02-9 Inhibits Cervical Cancer Cell Migration / Invasion and Angiogenesis via MAPK14 Signaling. *Gynecol. Oncol.* **2013**, *131* (3), 734–743. https://doi.org/10.1016/j.ygyno.2013.10.012.

169. Hattori, H.; Okuda, K.; Murase, T.; Shigetsura, Y.; Narise, K.; Semenza, G. L.; Nagasawa, H. Isolation, Identification, and Biological Evaluation of HIF-1-Modulating Compounds from Brazilian Green Propolis. *Bioorganic Med. Chem.* **2011**, *19* (18), 5392–5401. https://doi.org/10.1016/j.bmc.2011.07.060.

170. Ramchandani, S.; Naz, I.; Lee, J. H.; Khan, M. R.; Ahn, K. S. An Overview of the Potential Antineoplastic Effects of Casticin. *Molecules* **2020**, *25* (6), 1287. https://doi.org/10.3390/molecules25061287.

171. Yang, D. S.; Li, Z. L.; Peng, W. B.; Yang, Y. P.; Wang, X.; Liu, K. C.; Li, X. L.; Xiao, W. L. Three New Prenylated Flavonoids from Macaranga Denticulata and Their Anticancer Effects. *Fitoterapia* **2015**, *103*, 165–170. https://doi.org/10.1016/j.fitote.2015.04.001.

172. Kashyap, D.; Sharma, A.; Sak, K.; Tuli, H. S.; Buttar, H. S.; Bishayee, A. Fisetin: A Bioactive Phytochemical with Potential for Cancer Prevention and Pharmacotherapy. *Life Sci.* **2018**, *194*, 75–87. https://doi.org/10.1016/j.lfs.2017.12.005.

173. Rengarajan, T.; Yaacob, N. S. The Flavonoid Fisetin as an Anticancer Agent Targeting the Growth Signaling Pathways. *Eur. J. Pharmacol.* **2016**, *789*, 8–16. https://doi.org/10.1016/j.ejphar.2016.07.001.

174. Syed, D. N.; Adhami, V. M.; Khan, N.; Khan, M. I.; Mukhtar, H. Exploring the Molecular Targets of Dietary Flavonoid Fisetin in Cancer. *Semin. Cancer Biol.* **2016**, *40*, 130–140. https://doi.org/10.1016/j.semcancer.2016.04.003.

175. Rahul K. Lall, Vaqar Mustafa Adhami, and H. M. Dietary Flavonoid Fisetin for Cancer Prevention and Treatment. *Mol. Nutr. Food Res.* **2016**, *60* (6), 1396–1405. https://doi.org/10.1016/j.physbeh.2017.03.040.

176. Tsai, C. F.; Chen, J. H.; Chang, C. N.; Lu, D. Y.; Chang, P. C.; Wang, S. L.; Yeh, W. L. Fisetin Inhibits Cell Migration via Inducing HO-1 and Reducing MMPs Expression in Breast Cancer Cell Lines. *Food Chem. Toxicol.* **2018**, *120*, 528–535. https://doi.org/10.1016/j.fct.2018.07.059.

177. Sun, X.; Ma, X.; Li, Q.; Yang, Y.; Xu, X.; Sun, J.; Yu, M.; Cao, K.; Yang, L.; Yang, G.; Zhang, G.; Wang, X. Anti‑cancer Effects of Fisetin on Mammary Carcinoma Cells via Regulation of the PI3K/Akt/MTOR Pathway: In Vitro and in Vivo Studies. *Int. J. Mol. Med.* **2018**, *42* (2), 811–820. https://doi.org/10.3892/ijmm.2018.3654.

178. Li, J.; Gong, X.; Jiang, R.; Lin, D.; Zhou, T.; Zhang, A.; Li, H.; Zhang, X.; Wan, J.; Kuang, G.; Li, H. Fisetin Inhibited Growth and Metastasis of Triple-Negative Breast Cancer by Reversing Epithelial-to-Mesenchymal Transition via PTEN/Akt/GSK3β Signal Pathway. *Front. Pharmacol.* **2018**, *9*, 1–14. https://doi.org/10.3389/fphar.2018.00772.

179. Chou, R. H.; Hsieh, S. C.; Yu, Y. L.; Huang, M. H.; Huang, Y. C.; Hsieh, Y. H. Fisetin Inhibits Migration and Invasion of Human Cervical Cancer Cells by Down-Regulating Urokinase Plasminogen Activator Expression through Suppressing the P38 MAPK-Dependent NF-ΚB Signaling Pathway. *PLoS One* **2013**, *8* (8), 1–12. https://doi.org/10.1371/journal.pone.0071983.

180. Chen, C. M.; Hsieh, Y. H.; Hwang, J. M.; Jan, H. J.; Hsieh, S. C.; Lin, S. H.; Lai, C. Y. Fisetin Suppresses ADAM9 Expression and Inhibits Invasion of Glioma Cancer Cells through Increased Phosphorylation of ERK1/2. *Tumor Biol.* **2015**, *36* (5), 3407–3415. https://doi.org/10.1007/s13277-014-2975-9.

181. Liu, X. F.; Long, H. J.; Miao, X. Y.; Liu, G. L.; Yao, H. L. Fisetin Inhibits Liver Cancer Growth in a Mouse Model: Relation to Dopamine Receptor. *Oncol. Rep.* **2017**, *38* (1), 53–62. https://doi.org/10.3892/or.2017.5676.

182. Klimaszewska‑Wiśniewska, A.; Grzanka, D.; Czajkowska, P.; Hałas‑Wiśniewska, M.; Durślewicz, J.; Antosik, P.; Grzanka, A.; Gagat, M. Cellular and Molecular Alterations Induced by Low‑dose Fisetin in Human Chronic Myeloid Leukemia Cells. *Int. J. Oncol.* **2019**, *55* (6), 1261–1274. https://doi.org/10.3892/ijo.2019.4889.

183. Tabasum, S.; Singh, R. P. Fisetin Suppresses Migration, Invasion and Stem-Cell-like Phenotype of Human Non-Small Cell Lung Carcinoma Cells via Attenuation of Epithelial to Mesenchymal Transition. *Chem. Biol. Interact.* **2019**, *303*, 14–21. https://doi.org/10.1016/j.cbi.2019.02.020.

184. Wang, J.; Huang, S. Fisetin Inhibits the Growth and Migration in the A549 Human Lung Cancer Cell Line via the ERK1/2 Pathway. *Exp. Ther. Med.* **2018**, *15* (3), 2667–2673. https://doi.org/10.3892/etm.2017.5666.

185. Chien, C. S.; Shen, K. H.; Huang, J. S.; Ko, S. C.; Shih, Y. W. Antimetastatic Potential of Fisetin Involves Inactivation of the PI3K/Akt and JNK Signaling Pathways with Downregulation of MMP-2/9 Expressions in Prostate Cancer PC-3 Cells. *Mol. Cell. Biochem.* **2010**, *333* (1–2), 169–180. https://doi.org/10.1007/s11010-009-0217-z.

186. Hsieh, Y. S.; Tsai; Yang; Chiou; Lin; Hsieh; Chang. Fisetin Suppresses the Proliferation and Metastasis of Renal Cell Carcinoma through Upregulation of MEK/ERK-Targeting CTSS and ADAM9. *Cells* **2019**, *8* (9), 948. https://doi.org/10.3390/cells8090948.

187. Fang, D.; Xiong, Z.; Xu, J.; Yin, J.; Luo, R. Chemopreventive Mechanisms of Galangin against Hepatocellular Carcinoma: A Review. *Biomed. Pharmacother.* **2019**, *109*, 2054–2061. https://doi.org/10.1016/j.biopha.2018.09.154.

188. Kim, J. D.; Liu, L.; Guo, W.; Meydani, M. Chemical Structure of Flavonols in Relation to Modulation of Angiogenesis and Immune-Endothelial Cell Adhesion. *J. Nutr. Biochem.* **2006**, *17* (3), 165–176. https://doi.org/10.1016/j.jnutbio.2005.06.006.

189. Chen, D.; Li, D.; Xu, X. bing; Qiu, S.; Luo, S.; Qiu, E.; Rong, Z.; Zhang, J.; Zheng, D. Galangin Inhibits Epithelial-Mesenchymal Transition and Angiogenesis by Downregulating CD44 in Glioma. *J. Cancer* **2019**, *10* (19), 4499–4508. https://doi.org/10.7150/jca.31487.

190. Lei, D.; Zhang, F.; Yao, D.; Xiong, N.; Jiang, X.; Zhao, H. Galangin Increases ERK1/2 Phosphorylation to Decrease ADAM9 Expression and Prevents Invasion in A172 Glioma Cells. *Mol. Med. Rep.* **2018**, *17* (1), 667–673. https://doi.org/10.3892/mmr.2017.7920.

191. Chien, S. T.; Shi, M. Der; Lee, Y. C.; Te, C. C.; Shih, Y. W. Galangin, a Novel Dietary Flavonoid, Attenuates Metastatic Feature via PKC/ERK Signaling Pathway in TPA-Treated Liver Cancer HepG2 Cells. *Cancer Cell Int.* **2015**, *15* (1), 1–11. https://doi.org/10.1186/s12935-015-0168-2.

192. Huang, H.; Chen, A. Y.; Rojanasakul, Y.; Ye, X.; Rankin, G. O.; Chen, Y. C. Dietary Compounds Galangin and Myricetin Suppress Ovarian Cancer Cell Angiogenesis. *J. Funct. Foods* **2015**, *15*, 464–475. https://doi.org/10.1016/j.jff.2015.03.051.

193. Cao, J.; Wang, H.; Chen, F.; Fang, J.; Xu, A.; Xi, W.; Zhang, S.; Wu, G.; Wang, Z. Galangin Inhibits Cell Invasion by Suppressing the Epithelial-Mesenchymal Transition and Inducing Apoptosis in Renal Cell Carcinoma. *Mol. Med. Rep.* **2016**, *13* (5), 4238–4244. https://doi.org/10.3892/mmr.2016.5042.

194. Zhu, Y.; Rao, Q.; Zhang, X.; Zhou, X. Galangin Induced Antitumor Effects in Human Kidney Tumor Cells Mediated via Mitochondrial Mediated Apoptosis, Inhibition of Cell Migration and Invasion and Targeting PI3K/ AKT/MTOR Signalling Pathway. *J. B.U.ON.* **2018**, *23* (3), 795–799.

195. Li Wang, Xiangyu Wang, Hanyong Chen, Xueyin Zu, Fayang Ma, Kangdong Liu, Ann M. Bode, Zigang Dong, and D. J. K. Gossypin Inhibits Gastric Cancer Growth by Direct Targeting AURKA and RSK2. *Phyther. Res.* **2019**, *33* (3), 640–650. https://doi.org/10.1016/j.gde.2016.03.011.

196. Li, L.; Fan, P.; Chou, H.; Li, J.; Wang, K.; Li, H. Herbacetin Suppressed MMP9 Mediated Angiogenesis of Malignant Melanoma through Blocking EGFR-ERK / AKT Signaling Pathway. *Biochimie* **2019**, *162*, 198–207. https://doi.org/10.1016/j.biochi.2019.05.003.

197. Jin, X. N.; Yan, E. Z.; Wang, H. M.; Sui, H. J.; Liu, Z.; Gao, W.; Jin, Y. Hyperoside Exerts Anti-Inflammatory and Anti-Arthritic Effects in LPS-Stimulated Human Fibroblast-like Synoviocytes in Vitro and in Mice with Collagen-Induced Arthritis. *Acta Pharmacol. Sin.* **2016**, *37* (5), 674–686. https://doi.org/10.1038/aps.2016.7.

198. Zhang, X.; Liu, T.; Huang, Y.; Wismeijer, D.; Liu, Y. Icariin: Does It Have an Osteoinductive Potential for Bone Tissue Engineering? *Phyther. Res.* **2014**, *28* (4), 498–509. https://doi.org/10.1002/ptr.5027.

199. Chen, M.; Wu, J.; Luo, Q.; Mo, S.; Lyu, Y.; Wei, Y.; Dong, J. The Anticancer Properties of Herba Epimedii and Its Main Bioactive Componentsicariin and Icariside II. *Nutrients* **2016**, *8* (9), 1–19. https://doi.org/10.3390/nu8090563.

200. Chung, B. H.; Kim, J. D.; Kim, C. K.; Kim, J. W.; Won, M. H.; Lee, H. S.; Dong, M. S.; Ha, K. S.; Kwon, Y. G.; Kim, Y. M. Icariin Stimulates Angiogenesis by Activating the MEK/ERK- and PI3K/Akt/ENOS-Dependent Signal Pathways in Human Endothelial Cells. *Biochem. Biophys. Res. Commun.* **2008**, *376* (2), 404–408. https://doi.org/10.1016/j.bbrc.2008.09.001.

201. Gu, Z. F.; Zhang, Z. T.; Wang, J. Y.; Xu, B. Bin. Icariin Exerts Inhibitory Effects on the Growth and Metastasis of KYSE70 Human Esophageal Carcinoma Cells via PI3K/AKT and STAT3 Pathways. *Environ. Toxicol. Pharmacol.* **2017**, *54*, 7–13. https://doi.org/10.1016/j.etap.2017.06.004.

202. Wang, P.; Zhang, J.; Xiong, X.; Yuan, W.; Qin, S.; Cao, W.; Dai, L.; Xie, F.; Li, A.; Liu, Z. Icariin Suppresses Cell Cycle Transition and Cell Migration in Ovarian Cancer Cells. *Oncol. Rep.* **2019**, *41* (4), 2321–2328. https://doi.org/10.3892/or.2019.6986.

203. Singh, W. R.; Devi, H. S.; Kumawat, S.; Sadam, A.; Appukuttan, A. V.; Patel, M. R.; Lingaraju, M. C.; Singh, T. U.; Kumar, D. Angiogenic and MMPs Modulatory Effects of Icariin Improved Cutaneous Wound Healing in Rats. *Eur. J. Pharmacol.* **2019**, *858*, 172466. https://doi.org/10.1016/j.ejphar.2019.172466.

204. Quan, K.; Zhang, X.; Fan, K.; Liu, P.; Yue, Q.; Li, B.; Wu, J.; Liu, B.; Xu, Y.; Hua, W.; Zhu, W. Icariside II Induces Cell Cycle Arrest and Apoptosis in Human Glioblastoma Cells through Suppressing Akt Activation and Potentiating FOXO3A Activity. *Am. J. Transl. Res.* **2017**, *9* (5), 2508–2519.

205. Xing, S.; Yu, W.; Zhang, X.; Luo, Y.; Lei, Z.; Huang, D.; Lin, J.; Huang, Y.; Huang, S.; Nong, F.; Zhou, C.; Wei, G. Isoviolanthin Extracted from Dendrobium Officinale Reverses TGF-Β1-Mediated Epithelial–Mesenchymal Transition in Hepatocellular Carcinoma Cells via Deactivating the TGF-β/Smad and PI3K/Akt/MTOR Signaling Pathways. *Int. J. Mol. Sci.* **2018**, *19* (6), 1–17. https://doi.org/10.3390/ijms19061556.

206. Chen, A. Y.; Chen, Y. C. A Review of the Dietary Flavonoid, Kaempferol on Human Health and Cancer Chemoprevention. *Food Chem.* **2013**, *138* (4), 2099–2107. https://doi.org/10.1016/j.foodchem.2012.11.139.

207. Kashyap, D.; Sharma, A.; Tuli, H. S.; Sak, K.; Punia, S.; Mukherjee, T. K. Kaempferol – A Dietary Anticancer Molecule with Multiple Mechanisms of Action: Recent Trends and Advancements. *J. Funct. Foods* **2017**, *30*, 203–219. https://doi.org/10.1016/j.jff.2017.01.022.

208. Chin, H. K.; Horng, C. T.; Liu, Y. S.; Lu, C. C.; Su, C. Y.; Chen, P. S.; Chiu, H. Y.; Tsai, F. J.; Shieh, P. C.; Yang, J. S. Kaempferol Inhibits Angiogenic Ability by Targeting VEGF Receptor-2 and Downregulating the PI3K/AKT, MEK and ERK Pathways in VEGF-Stimulated Human Umbilical Vein Endothelial Cells. *Oncol. Rep.* **2018**, *39* (5), 2351–2357. https://doi.org/10.3892/or.2018.6312.

209. Liang, F.; Han, Y.; Gao, H.; Xin, S.; Chen, S.; Wang, N.; Qin, W.; Zhong, H.; Lin, S.; Yao, X.; Li, S. Kaempferol Identified by Zebrafish Assay and Fine Fractionations Strategy from Dysosma Versipellis Inhibits Angiogenesis through VEGF and FGF Pathways. *Sci. Rep.* **2015**, *5*, 1–10. https://doi.org/10.1038/srep14468.

210. Kumazawa, S.; Kubota, S.; Yamamoto, H.; Okamura, N.; Sugiyama, Y.; Kobayashi, H.; Nakanishi, M.; Ohta, T. Antiangiogenic Activity of Flavonoids from Melia Azedarach. *Nat. Prod. Commun.* **2013**, *8* (12), 1719–1720. https://doi.org/10.1177/1934578x1300801215.

211. Özay, Y.; Güzel, S.; Yumrutaş, Ö.; Pehlivanoğlu, B.; Erdoğdu, İ. H.; Yildirim, Z.; Türk, B. A.; Darcan, S. Wound Healing Effect of Kaempferol in Diabetic and Nondiabetic Rats. *J. Surg. Res.* **2019**, *233*, 284–296. https://doi.org/10.1016/j.jss.2018.08.009.

212. Sharma, V.; Joseph, C.; Ghosh, S.; Agarwal, A.; Mishra, M. K.; Sen, E. Kaempferol Induces Apoptosis in Glioblastoma Cells through Oxidative Stress. *Mol. Cancer Ther.* **2007**, *6* (9), 2544–2553. https://doi.org/10.1158/1535-7163.MCT-06-0788.

213. Qin, Y.; Cui, W.; Yang, X.; Tong, B. Kaempferol Inhibits the Growth and Metastasis of Cholangiocarcinoma in Vitro and in Vivo. *Acta Biochim. Biophys. Sin. (Shanghai).* **2015**, *48* (3), 238–245. https://doi.org/10.1093/abbs/gmv133.

214. Zhu, G.; Liu, X.; Li, H.; Yan, Y.; Hong, X.; Lin, Z. Kaempferol Inhibits Proliferation, Migration, and Invasion of Liver Cancer HepG2 Cells by down-Regulation of MicroRNA-21. *Int. J. Immunopathol. Pharmacol.* **2018**, *32*, 2058738418814341. https://doi.org/10.1177/2058738418814341.

215. Jo, E.; Park, S. J.; Choi, Y. S.; Jeon, W. K.; Kim, B. C. Kaempferol Suppresses Transforming Growth Factor-Β1-Induced Epithelial-to-Mesenchymal Transition and Migration of A549 Lung Cancer Cells by Inhibiting Akt1-Mediated Phosphorylation of Smad3 at Threonine-179. *Neoplasia* **2015**, *17* (7), 525–537. https://doi.org/10.1016/j.neo.2015.06.004.

216. Labbé, D.; Provençal, M.; Lamy, S.; Boivin, D.; Gingras, D.; Béliveau, R. The Flavonols Quercetin, Kaempferol, and Myricetin Inhibit Hepatocyte Growth. *J. Nutr. Biochem. Mol. Genet. Mech.* **2009**, *139*, 646–652. https://doi.org/10.3945/jn.108.102616.kinase.

217. Lin, C. W.; Chen, P. N.; Chen, M. K.; Yang, W. E.; Tang, C. H.; Yang, S. F.; Hsieh, Y. S. Kaempferol Reduces Matrix Metalloproteinase-2 Expression by down-Regulating ERK1/2 and the Activator Protein-1 Signaling Pathways in Oral Cancer Cells. *PLoS One* **2013**, *8* (11), 80883. https://doi.org/10.1371/journal.pone.0080883.

218. Chen, H. J.; Lin, C. M.; Lee, C. Y.; Shih, N. C.; Peng, S. F.; Tsuzuki, M.; Amagaya, S.; Huang, W. W.; Yang, J. S. Kaempferol Suppresses Cell Metastasis via Inhibition of the ERK-P38-JNK and AP-1 Signaling Pathways in U-2 OS Human Osteosarcoma Cells. *Oncol. Rep.* **2013**, *30* (2), 925–932. https://doi.org/10.3892/or.2013.2490.

219. Luo, H.; Rankin, G. O.; Liu, L.; Daddysman, M. K.; Jiang, B. H.; Chen, Y. C. Kaempferol Inhibits Angiogenesis and VEGF Expression through Both HIF Dependent and Independent Pathways in Human Ovarian Cancer Cells. *Nutr. Cancer* **2009**, *61* (4), 554–563. https://doi.org/10.1080/01635580802666281.

220. Lee, J.; Kim, J. H. Kaempferol Inhibits Pancreatic Cancer Cell Growth and Migration through the Blockade of EGFR-Related Pathway in Vitro. *PLoS One* **2016**, *11* (5), 1–14. https://doi.org/10.1371/journal.pone.0155264.

221. Hung, T. W.; Chen, P. N.; Wu, H. C.; Wu, S. W.; Tsai, P. Y.; Hsieh, Y. S.; Chang, H. R. Kaempferol Inhibits the Invasion and Migration of Renal Cancer Cells through the Downregulation of AKT and FAK Pathways. *Int. J. Med. Sci.* **2017**, *14* (10), 984–993. https://doi.org/10.7150/ijms.20336.

222. Chien, H. W.; Wang, K.; Chang, Y. Y.; Hsieh, Y. H.; Yu, N. Y.; Yang, S. F.; Lin, H. W. Kaempferol Suppresses Cell Migration through the Activation of the ERK Signaling Pathways in ARPE-19 Cells. *Environ. Toxicol.* **2019**, *34* (3), 312–318. https://doi.org/10.1002/tox.22686.

223. Clericuzio, M.; Tinello, S.; Burlando, B.; Ranzato, E.; Martinotti, S.; Cornara, L.; La Rocca, A. Flavonoid Oligoglycosides from Ophioglossum Vulgatum L. Having Wound Healing Properties. *Planta Med.* **2012**, *78* (15), 1639–1644. https://doi.org/10.1055/s-0032-1315149.

224. Zeng, N.; Tong, B.; Zhang, X.; Dou, Y.; Wu, X.; Xia, Y.; Dai, Y.; Wei, Z. Antiarthritis Effect of Morin Is Associated with Inhibition of Synovial Angiogensis. *Drug Dev. Res.* **2015**, *76* (8), 463–473. https://doi.org/10.1002/ddr.21282.

225. Yue, M.; Zeng, N.; Xia, Y.; Wei, Z.; Dai, Y. Morin Exerts Anti-Arthritic Effects by Attenuating Synovial Angiogenesis via Activation of Peroxisome Proliferator Activated Receptor-γ. *Mol. Nutr. Food Res.* **2018**, *62* (21), 1–13. https://doi.org/10.1002/mnfr.201800202.

226. Capitani, N.; Lori, G.; Paoli, P.; Patrussi, L.; Troilo, A.; Baldari, C. T.; Raugei, G.; D’Elios, M. M. LMW-PTP Targeting Potentiates the Effects of Drugs Used in Chronic Lymphocytic Leukemia Therapy. *Cancer Cell Int.* **2019**, *19* (1), 1–9. https://doi.org/10.1186/s12935-019-0786-1.

227. Li, H. W.; Zou, T. Bin; Jia, Q.; Xia, E. Q.; Cao, W. J.; Liu, W.; He, T. P.; Wang, Q. Anticancer Effects of Morin-7-Sulphate Sodium, a Flavonoid Derivative, in Mouse Melanoma Cells. *Biomed. Pharmacother.* **2016**, *84*, 909–916. https://doi.org/10.1016/j.biopha.2016.10.001.

228. Kang, N. J.; Jung, S. K.; Lee, K. W.; Lee, H. J. Myricetin Is a Potent Chemopreventive Phytochemical in Skin Carcinogenesis. *Ann. N. Y. Acad. Sci.* **2011**, *1229* (1), 124–132. https://doi.org/10.1111/j.1749-6632.2011.06122.x.

229. Zhou, Z.; Mao, W.; Li, Y.; Qi, C.; He, Y. Myricetin Inhibits Breast Tumor Growth and Angiogenesis by Regulating VEGF/VEGFR2 and P38MAPK Signaling Pathways. *Anat. Rec.* **2019**, *302* (12), 2186–2192. https://doi.org/10.1002/ar.24222.

230. Ci, Y.; Zhang, Y.; Liu, Y.; Lu, S.; Cao, J.; Li, H.; Zhang, J.; Huang, Z.; Zhu, X.; Gao, J.; Han, M. Myricetin Suppresses Breast Cancer Metastasis through Down-Regulating the Activity of Matrix Metalloproteinase (MMP)-2/9. *Phyther. Res.* **2018**, *32* (7), 1373–1381. https://doi.org/10.1002/ptr.6071.

231. Yamada, N.; Matsushima-Nishiwaki, R.; Kozawa, O. Quercetin Suppresses the Migration of Hepatocellular Carcinoma Cells Stimulated by Hepatocyte Growth Factor or Transforming Growth Factor-α: Attenuation of AKT Signaling Pathway. *Arch. Biochem. Biophys.* **2020**, *682*, 108296. https://doi.org/10.1016/j.abb.2020.108296.

232. Ma, H.; Zhu, L.; Ren, J.; Rao, B.; Sha, M.; Kuang, Y.; Shen, W.; Xu, Z. Myricetin Inhibits Migration and Invasion of Hepatocellular Carcinoma MHCC97H Cell Line by Inhibiting the EMT Process. *Oncol. Lett.* **2019**, *18* (6), 6614–6620. https://doi.org/10.3892/ol.2019.10998.

233. Shih, Y. W.; Wu, P. F.; Lee, Y. C.; Shi, M. Der; Chiang, T. A. Myricetin Suppresses Invasion and Migration of Human Lung Adenocarcinoma A549 Cells: Possible Mediation by Blocking the Erk Signaling Pathway. *J. Agric. Food Chem.* **2009**, *57* (9), 3490–3499. https://doi.org/10.1021/jf900124r.

234. Ezzati, M.; Yousefi, B.; Velaei, K.; Safa, A. A Review on Anti-Cancer Properties of Quercetin in Breast Cancer. *Life Sci.* **2020**, *248*, 117463. https://doi.org/10.1016/j.lfs.2020.117463.

235. Tang, S.; Deng, X.; Zhou, J.; Li, Q.; Ge, X.; Miao, L. Pharmacological Basis and New Insights of Quercetin Action in Respect to Its Anti-Cancer Effects. *Biomed. Pharmacother.* **2020**, *121*, 109604. https://doi.org/10.1016/j.biopha.2019.109604.

236. Kashyap, D.; Mittal, S.; Sak, K.; Singhal, P.; Tuli, H. S. Molecular Mechanisms of Action of Quercetin in Cancer: Recent Advances. *Tumor Biol.* **2016**, *37* (10), 12927–12939. https://doi.org/10.1007/s13277-016-5184-x.

237. Darband, S. G.; Kaviani, M.; Yousefi, B.; Sadighparvar, S.; Pakdel, F. G.; Attari, J. A.; Mohebbi, I.; Naderi, S.; Majidinia, M. Quercetin: A Functional Dietary Flavonoid with Potential Chemo-Preventive Properties in Colorectal Cancer. *J. Cell. Physiol.* **2018**, *233* (9), 6544–6560. https://doi.org/10.1002/jcp.26595.

238. Song, N. R.; Chung, M. Y.; Kang, N. J.; Seo, S. G.; Jang, T. S.; Lee, H. J.; Lee, K. W. Quercetin Suppresses Invasion and Migration of H-Ras-Transformed MCF10A Human Epithelial Cells by Inhibiting Phosphatidylinositol 3-Kinase. *Food Chem.* **2014**, *142*, 66–71. https://doi.org/10.1016/j.foodchem.2013.07.002.

239. Lin, C.; Wu, M.; Dong, J. Quercetin-4′-o-β-d-Glucopyranoside (QODG) Inhibits Angiogenesis by Suppressing VEGFR2-Mediated Signaling in Zebrafish and Endothelial Cells. *PLoS One* **2012**, *7* (2), 31708. https://doi.org/10.1371/journal.pone.0031708.

240. Lee, Y. H.; Tuyet, P. T. Synthesis and Biological Evaluation of Quercetin–Zinc (II) Complex for Anti-Cancer and Anti-Metastasis of Human Bladder Cancer Cells. *Vitr. Cell. Dev. Biol. - Anim.* **2019**, *55* (6), 395–404. https://doi.org/10.1007/s11626-019-00363-2.

241. Zhao, X.; Wang, Q.; Yang, S.; Chen, C.; Li, X.; Liu, J.; Zou, Z.; Cai, D. Quercetin Inhibits Angiogenesis by Targeting Calcineurin in the Xenograft Model of Human Breast Cancer. *Eur. J. Pharmacol.* **2016**, *781*, 60–68. https://doi.org/10.1016/j.ejphar.2016.03.063.

242. Oh, S. J.; Kim, O.; Lee, J. S.; Kim, J. A.; Kim, M. R.; Choi, H. S.; Shim, J. H.; Kang, K. W.; Kim, Y. C. Inhibition of Angiogenesis by Quercetin in Tamoxifen-Resistant Breast Cancer Cells. *Food Chem. Toxicol.* **2010**, *48* (11), 3227–3234. https://doi.org/10.1016/j.fct.2010.08.028.

243. Srinivasan, A.; Thangavel, C.; Liu, Y.; Shoyele, S.; Den, R. B.; Selvakumar, P.; Lakshmikuttyamma, A. Quercetin Regulates β-Catenin Signaling and Reduces the Migration of Triple Negative Breast Cancer. *Mol. Carcinog.* **2016**, *55* (5), 743–756. https://doi.org/10.1002/mc.22318.

244. Lin, C. W.; Hou, W. C.; Shen, S. C.; Juan, S. H.; Ko, C. H.; Wang, L. M.; Chen, Y. C. Quercetin Inhibition of Tumor Invasion via Suppressing PKCδ/ERK/ AP-1-Dependent Matrix Metalloproteinase-9 Activation in Breast Carcinoma Cells. *Carcinogenesis* **2008**, *29* (9), 1807–1815. https://doi.org/10.1093/carcin/bgn162.

245. Rivera Rivera, A.; Castillo-Pichardo, L.; Gerena, Y.; Dharmawardhane, S. Anti-Breast Cancer Potential of Quercetin via the Akt/AMPK/Mammalian Target of Rapamycin (MTOR) Signaling Cascade. *PLoS One* **2016**, *11* (6), 0157251. https://doi.org/10.1371/journal.pone.0157251.

246. Zhao, D.; Qin, C.; Fan, X.; Li, Y.; Gu, B. Inhibitory Effects of Quercetin on Angiogenesis in Larval Zebra Fi Sh and Human Umbilical Vein Endothelial Cells. *Eur. J. Pharmacol.* **2014**, *723*, 360–367. https://doi.org/10.1016/j.ejphar.2013.10.069.

247. Tan, W. F.; Lin, L. P.; Li, M. H.; Zhang, Y. X.; Tong, Y. G.; Xiao, D.; Ding, J. Quercetin, a Dietary-Derived Flavonoid, Possesses Antiangiogenic Potential. *Eur. J. Pharmacol.* **2003**, *459* (2–3), 255–262. https://doi.org/10.1016/S0014-2999(02)02848-0.

248. Lee, D. E.; Chung, M. Y.; Lim, T. G.; Huh, W. B.; Lee, H. J.; Lee, K. W. Quercetin Suppresses Intracellular Ros Formation, MMP Activation, and Cell Motility in Human Fibrosarcoma Cells. *J. Food Sci.* **2013**, *78* (9). https://doi.org/10.1111/1750-3841.12223.

249. Kee, J. Y.; Han, Y. H.; Kim, D. S.; Mun, J. G.; Park, J.; Jeong, M. Y.; Um, J. Y.; Hong, S. H. Inhibitory Effect of Quercetin on Colorectal Lung Metastasis through Inducing Apoptosis, and Suppression of Metastatic Ability. *Phytomedicine* **2016**, *23* (13), 1680–1690. https://doi.org/10.1016/j.phymed.2016.09.011.

250. Pan, H. C.; Jiang, Q.; Yu, Y.; Mei, J. P.; Cui, Y. K.; Zhao, W. J. Quercetin Promotes Cell Apoptosis and Inhibits the Expression of MMP-9 and Fibronectin via the AKT and ERK Signalling Pathways in Human Glioma Cells. *Neurochem. Int.* **2015**, *80*, 60–71. https://doi.org/10.1016/j.neuint.2014.12.001.

251. Liu, Y.; Tang, Z. G.; Lin, Y.; Qu, X. G.; Lv, W.; Wang, G. Bin; Li, C. L. Effects of Quercetin on Proliferation and Migration of Human Glioblastoma U251 Cells. *Biomed. Pharmacother.* **2017**, *92*, 33–38. https://doi.org/10.1016/j.biopha.2017.05.044.

252. Michaud-Levesque, J.; Bousquet-Gagnon, N.; Béliveau, R. Quercetin Abrogates IL-6/STAT3 Signaling and Inhibits Glioblastoma Cell Line Growth and Migration. *Exp. Cell Res.* **2012**, *318* (8), 925–935. https://doi.org/10.1016/j.yexcr.2012.02.017.

253. da Silva, A. B.; Cerqueira Coelho, P. L.; das Neves Oliveira, M.; Oliveira, J. L.; Oliveira Amparo, J. A.; da Silva, K. C.; Soares, J. R. P.; Pitanga, B. P. S.; dos Santos Souza, C.; de Faria Lopes, G. P.; da Silva, V. D. A.; de Fátima Dias Costa, M.; Junier, M. P.; Chneiweiss, H.; Moura-Neto, V.; Costa, S. L. The Flavonoid Rutin and Its Aglycone Quercetin Modulate the Microglia Inflammatory Profile Improving Antiglioma Activity. *Brain. Behav. Immun.* **2020**, *85*, 170–185. https://doi.org/10.1016/j.bbi.2019.05.003.

254. Liu, Y.; Tang, Z. G.; Yang, J. Q.; Zhou, Y.; Meng, L. H.; Wang, H.; Li, C. L. Low Concentration of Quercetin Antagonizes the Invasion and Angiogenesis of Human Glioblastoma U251 Cells. *Onco. Targets. Ther.* **2017**, *10*, 4023–4028. https://doi.org/10.2147/OTT.S136821.

255. Lu, J.; Wang, Z.; Li, S.; Xin, Q.; Yuan, M.; Li, H.; Song, X.; Gao, H.; Pervaiz, N.; Sun, X.; Lv, W.; Jing, T.; Zhu, Y. Quercetin Inhibits the Migration and Invasion of HCCLM3 Cells by Suppressing the Expression of P-Akt1, Matrix Metalloproteinase (MMP) MMP-2, and MMP-9. *Med. Sci. Monit.* **2018**, *24*, 2583–2589. https://doi.org/10.12659/MSM.906172.

256. Klimaszewska-Wiśniewska, A.; Hałas-Wiśniewska, M.; Izdebska, M.; Gagat, M.; Grzanka, A.; Grzanka, D. Antiproliferative and Antimetastatic Action of Quercetin on A549 Non-Small Cell Lung Cancer Cells through Its Effect on the Cytoskeleton. *Acta Histochem.* **2017**, *119* (2), 99–112. https://doi.org/10.1016/j.acthis.2016.11.003.

257. Hwang, M. K.; Song, N. R.; Kang, N. J.; Lee, K. W.; Lee, H. J. Activation of Phosphatidylinositol 3-Kinase Is Required for Tumor Necrosis Factor-α-Induced Upregulation of Matrix Metalloproteinase-9: Its Direct Inhibition by Quercetin. *Int. J. Biochem. Cell Biol.* **2009**, *41* (7), 1592–1600. https://doi.org/10.1016/j.biocel.2009.01.014.

258. Cao, H. H.; Cheng, C. Y.; Su, T.; Fu, X. Q.; Guo, H.; Li, T.; Tse, A. K. W.; Kwan, H. Y.; Yu, H.; Yu, Z. L. Quercetin Inhibits HGF/c-Met Signaling and HGFstimulated Melanoma Cell Migration and Invasion. *Mol. Cancer* **2015**, *14* (1), 1–12. https://doi.org/10.1186/s12943-015-0367-4.

259. Cao, H. H.; Tse, A. K. W.; Kwan, H. Y.; Yu, H.; Cheng, C. Y.; Su, T.; Fong, W. F.; Yu, Z. L. Quercetin Exerts Anti-Melanoma Activities and Inhibits STAT3 Signaling. *Biochem. Pharmacol.* **2014**, *87* (3), 424–434. https://doi.org/10.1016/j.bcp.2013.11.008.

260. Zhao, J.; Fang, Z.; Zha, Z.; Sun, Q.; Wang, H.; Sun, M.; Qiao, B. Quercetin Inhibits Cell Viability, Migration and Invasion by Regulating MiR-16/HOXA10 Axis in Oral Cancer. *Eur. J. Pharmacol.* **2019**, *847*, 11–18. https://doi.org/10.1016/j.ejphar.2019.01.006.

261. Nam, T. W.; Yoo, C. Il; Kim, H. T.; Kwon, C. H.; Park, J. Y.; Kim, Y. K. The Flavonoid Quercetin Induces Apoptosis and Inhibits Migration through a MAPK-Dependent Mechanism in Osteoblasts. *J. Bone Miner. Metab.* **2008**, *26* (6), 551–560. https://doi.org/10.1007/s00774-008-0864-2.

262. Li, S.; Pei, Y.; Wang, W.; Liu, F.; Zheng, K.; Zhang, X. Quercetin Suppresses the Proliferation and Metastasis of Metastatic Osteosarcoma Cells by Inhibiting Parathyroid Hormone Receptor 1. *Biomed. Pharmacother.* **2019**, *114*, 108839. https://doi.org/10.1016/j.biopha.2019.108839.

263. Lan, H.; Hong, W.; Fan, P.; Qian, D.; Zhu, J.; Bai, B. Quercetin Inhibits Cell Migration and Invasion in Human Osteosarcoma Cells. *Cell. Physiol. Biochem.* **2017**, *43* (2), 553–567. https://doi.org/10.1159/000480528.

264. Berndt, K.; Campanile, C.; Muff, R.; Strehler, E.; Born, W.; Fuchs, B. Evaluation of Quercetin as a Potential Drug in Osteosarcoma Treatment. *Anticancer Res.* **2013**, *33* (4), 1297–1306.

265. Dinglai Yu, Tingting Ye, Yukai Xiang, Zhehao Shi, Jie Zhang, Bin Lou, Fan Zhang, Bicheng Chen, and M. Z. Quercetin Inhibits Epithelial–Mesenchymal Transition, Decreases Invasiveness and Metastasis, and Reverses IL-6 Induced Epithelial–Mesenchymal Transition, Expression of MMP by Inhibiting STAT3 Signaling in Pancreatic Cancer Cells. *Onco. Targets. Ther.* **2017**, *10*, 4719–4729. https://doi.org/10.2147/OTT.S136840.

266. Bhat, F. A.; Sharmila, G.; Balakrishnan, S.; Arunkumar, R.; Elumalai, P.; Suganya, S.; Raja Singh, P.; Srinivasan, N.; Arunakaran, J. Quercetin Reverses EGF-Induced Epithelial to Mesenchymal Transition and Invasiveness in Prostate Cancer (PC-3) Cell Line via EGFR/PI3K/Akt Pathway. *J. Nutr. Biochem.* **2014**, *25* (11), 1132–1139. https://doi.org/10.1016/j.jnutbio.2014.06.008.

267. Yang, F.; Jiang, X.; Song, L.; Wang, H.; Mei, Z.; Xu, Z.; Xing, N. Quercetin Inhibits Angiogenesis through Thrombospondin-1 Upregulation to Antagonize Human Prostate Cancer PC-3 Cell Growth in Vitro and in Vivo. *Oncol. Rep.* **2016**, *35* (3), 1602–1610. https://doi.org/10.3892/or.2015.4481.

268. Song, W.; Zhao, X.; Xu, J.; Zhang, H. Quercetin Inhibits Angiogenesis–Mediated Human Retinoblastoma Growth by Targeting Vascular Endothelial Growth Factor Receptor. *Oncol. Lett.* **2017**, *14* (3), 3343–3348. https://doi.org/10.3892/ol.2017.6623.

269. Ben Sghaier, M.; Pagano, A.; Mousslim, M.; Ammari, Y.; Kovacic, H.; Luis, J. Rutin Inhibits Proliferation, Attenuates Superoxide Production and Decreases Adhesion and Migration of Human Cancerous Cells. *Biomed. Pharmacother.* **2016**, *84*, 1972–1978. https://doi.org/10.1016/j.biopha.2016.11.001.

270. Chen, H.; Miao, Q.; Geng, M.; Liu, J.; Hu, Y.; Tian, L.; Pan, J.; Yang, Y. Anti-Tumor Effect of Rutin on Human Neuroblastoma Cell Lines through Inducing G2/M Cell Cycle Arrest and Promoting Apoptosis. *Sci. World J.* **2013**, *2013*. https://doi.org/10.1155/2013/269165.

271. Singhal, J.; Nagaprashantha, L.; Chikara, S.; Awasthi, S.; Horne, D.; Singhal, S. S. 2’-Hydroxyflavanone: A Novel Strategy for Targeting Breast Cancer. *Oncotarget* **2017**, *8* (43), 75025–75037. https://doi.org/10.18632/oncotarget.20499.

272. Wu, S.; Huang, J.; Hui, K.; Yue, Y.; Gu, Y.; Ning, Z.; Wang, X.; He, D.; Wu, K. 2’-Hydroxyflavanone Inhibits Epithelial-Mesenchymal Transition, and Cell Migration and Invasion via Suppression of the Wnt/ß-Catenin Signaling Pathway in Prostate Cancer. *Oncol. Rep.* **2018**, *40* (5), 2836–2843. https://doi.org/10.3892/or.2018.6678.

273. Zhang, X. L.; Cao, M. A.; Pu, L. P.; Huang, S. S.; Gao, Q. X.; Yuan, C. S.; Wang, C. M. A Novel Flavonoid Isolated from Sophora Flavescens Exhibited Anti-Angiogenesis Activity, Decreased VEGF Expression and Caused G0/G1 Cell Cycle Arrest in Vitro. *Pharmazie* **2013**, *68* (5), 369–375. https://doi.org/10.1691/ph.2013.2845.

274. Zhao, X.; Guo, X.; Shen, J.; Hua, D. Alpinetin Inhibits Proliferation and Migration of Ovarian Cancer Cells via Suppression of STAT3 Signaling. *Mol. Med. Rep.* **2018**, *18* (4), 4030–4036. https://doi.org/10.3892/mmr.2018.9420.

275. Li, Q.; Wang, Y.; Xiao, H.; Li, Y.; Kan, X.; Wang, X.; Zhang, G.; Wang, Z.; Yang, Q.; Chen, X.; Weng, X.; Chen, Y.; Zhou, B.; Guo, Y.; Liu, X.; Zhu, X. Chamaejasmenin B, a Novel Candidate, Inhibits Breast Tumor Metastasis by Rebalancing TGF-Beta Paradox. *Oncotarget* **2016**, *7* (30), 48180–48192. https://doi.org/10.18632/oncotarget.10193.

276. Li, W.; Du, Q.; Li, X.; Zheng, X.; Lv, F.; Xi, X.; Huang, G.; Yang, J.; Liu, S. Eriodictyol Inhibits Proliferation, Metastasis and Induces Apoptosis of Glioma Cells via PI3K/Akt/NF-ΚB Signaling Pathway. *Front. Pharmacol.* **2020**, *11*, 1–16. https://doi.org/10.3389/fphar.2020.00114.

277. Li, W.; Kandhare, A. D.; Mukherjee, A. A.; Bodhankar, S. L. Hesperidin, a Plant Flavonoid Accelerated the Cutaneous Wound Healing in Streptozotocin-Induced Diabetic Rats: Role of TGF-B/SMADS and ANG-1/TIE-2 Signaling Pathways. *EXCLI J.* **2018**, *17*, 399–419. https://doi.org/10.17179/excli2018-1036.

278. Byun, E. B.; Kim, H. M.; Song, H. Y.; Kim, W. S. Hesperidin Structurally Modified by Gamma Irradiation Induces Apoptosis in Murine Melanoma B16BL6 Cells and Inhibits Both Subcutaneous Tumor Growth and Metastasis in C57BL/6 Mice. *Food Chem. Toxicol.* **2019**, *127*, 19–30. https://doi.org/10.1016/j.fct.2019.02.042.

279. Lowe, H. I. C.; Toyang, N. J.; Watson, C. T.; Ayeah, K. N.; Bryant, J. HLBT-100: A Highly Potent Anti-Cancer Flavanone from Tillandsia Recurvata (L.) L. *Cancer Cell Int.* **2017**, *17* (1), 1–12. https://doi.org/10.1186/s12935-017-0404-z.

280. Shi, Q.; Jiang, Z.; Yang, J.; Cheng, Y.; Pang, Y.; Zheng, N.; Chen, J.; Chen, W.; Jia, L. A Flavonoid Glycoside Compound from Murraya Paniculata (L.) Interrupts Metastatic Characteristics of A549 Cells by Regulating STAT3/NF-ΚB/COX-2 and EGFR Signaling Pathways. *AAPS J.* **2017**, *19* (6), 1779–1790. https://doi.org/10.1208/s12248-017-0134-0.

281. Xie, S. R.; Wang, Y.; Liu, C. W.; Luo, K.; Cai, Y. Q. Liquiritigenin Inhibits Serum-Induced HIF-1α and VEGF Expression vi the AKT/MTOR-P70S6K Signalling Pathway in HeLa Cells. *Phyther. Res.* **2012**, *26* (8), 1133–1141. https://doi.org/10.1002/ptr.3696.

282. Aroui, S.; Aouey, B.; Chtourou, Y.; Meunier, A. C.; Fetoui, H.; Kenani, A. Naringin Suppresses Cell Metastasis and the Expression of Matrix Metalloproteinases (MMP-2 and MMP-9) via the Inhibition of ERK-P38-JNK Signaling Pathway in Human Glioblastoma. *Chem. Biol. Interact.* **2016**, *244*, 195–203. https://doi.org/10.1016/j.cbi.2015.12.011.

283. Aroui, S.; Najlaoui, F.; Chtourou, Y.; Meunier, A. C.; Laajimi, A.; Kenani, A.; Fetoui, H. Naringin Inhibits the Invasion and Migration of Human Glioblastoma Cell via Downregulation of MMP-2 and MMP-9 Expression and Inactivation of P38 Signaling Pathway. *Tumor Biol.* **2016**, *37* (3), 3831–3839. https://doi.org/10.1007/s13277-015-4230-4.

284. Pafumi, I.; Festa, M.; Papacci, F.; Lagostena, L.; Giunta, C.; Gutla, V.; Cornara, L.; Favia, A.; Palombi, F.; Gambale, F.; Filippini, A.; Carpaneto, A. Naringenin Impairs Two-Pore Channel 2 Activity and Inhibits VEGF-Induced Angiogenesis /631/67/2328 /631/80/86/1999 /9/74 /96/34 /64/60 /14/63 /96/63 Article. *Sci. Rep.* **2017**, *7* (1), 1–11. https://doi.org/10.1038/s41598-017-04974-1.

285. Chen, K. S.; Shi, M. Der; Chien, C. S.; Shih, Y. W. Pinocembrin Suppresses TGF-Β1-Induced Epithelial-Mesenchymal Transition and Metastasis of Human Y-79 Retinoblastoma Cells through Inactivating Αvβ3 Integrin/FAK/P38α Signaling Pathway. *Cell Biosci.* **2014**, *4* (1), 1–13. https://doi.org/10.1186/2045-3701-4-41.

286. Karas, D.; Ulrichová, J.; Valentová, K. Galloylation of Polyphenols Alters Their Biological Activity. *Food Chem. Toxicol.* **2017**, *105*, 223–240. https://doi.org/10.1016/j.fct.2017.04.021.

287. Hou, Z.; Lambert, J. D.; Chin, K. V.; Yang, C. S. Effects of Tea Polyphenols on Signal Transduction Pathways Related to Cancer Chemoprevention. *Mutat. Res. - Fundam. Mol. Mech. Mutagen.* **2004**, *555* (1–2), 3–19. https://doi.org/10.1016/j.mrfmmm.2004.06.040.

288. Neergheen, V. S.; Bahorun, T.; Taylor, E. W.; Jen, L. S.; Aruoma, O. I. Targeting Specific Cell Signaling Transduction Pathways by Dietary and Medicinal Phytochemicals in Cancer Chemoprevention. *Toxicology* **2010**, *278* (2), 229–241. https://doi.org/10.1016/j.tox.2009.10.010.

289. Chowdhury, A.; Sarkar, J.; Chakraborti, T.; Pramanik, P. K.; Chakraborti, S. Protective Role of Epigallocatechin-3-Gallate in Health and Disease: A Perspective. *Biomed. Pharmacother.* **2016**, *78*, 50–59. https://doi.org/10.1016/j.biopha.2015.12.013.

290. Doss, M. X.; Potta, S. P.; Hescheler, J.; Sachinidis, A. Trapping of Growth Factors by Catechins: A Possible Therapeutical Target for Prevention of Proliferative Diseases. *J. Nutr. Biochem.* **2005**, *16* (5), 259–266. https://doi.org/10.1016/j.jnutbio.2004.11.003.

291. Abdulkhaleq, L. A.; Assi, M. A.; Noor, M. H. M.; Abdullah, R.; Saad, M. Z.; Taufiq-Yap, Y. H. Therapeutic Uses of Epicatechin in Diabetes and Cancer. *Vet. World* **2017**, *10* (8), 869–872. https://doi.org/10.14202/vetworld.2017.869-872.

292. Katiyar, S.; Elmets, C. A.; Katiyar, S. K. Green Tea and Skin Cancer: Photoimmunology, Angiogenesis and DNA Repair. *J. Nutr. Biochem.* **2007**, *18* (5), 287–296. https://doi.org/10.1016/j.jnutbio.2006.08.004.

293. Saeed, M.; Naveed, M.; Arif, M.; Kakar, M. U.; Manzoor, R.; Abd El-Hack, M. E.; Alagawany, M.; Tiwari, R.; Khandia, R.; Munjal, A.; Karthik, K.; Dhama, K.; Iqbal, H. M. N.; Dadar, M.; Sun, C. Green Tea (Camellia Sinensis) and L-Theanine: Medicinal Values and Beneficial Applications in Humans—A Comprehensive Review. *Biomed. Pharmacother.* **2017**, *95*, 1260–1275. https://doi.org/10.1016/j.biopha.2017.09.024.

294. Singh, A. K.; Seth, P.; Anthony, P.; Husain, M. M.; Madhavan, S.; Mukhtar, H.; Maheshwari, R. K. Green Tea Constituent Epigallocatechin-3-Gallate Inhibits Angiogenic Differentiation of Human Endothelial Cells. *Arch. Biochem. Biophys.* **2002**, *401* (1), 29–37. https://doi.org/10.1016/S0003-9861(02)00013-9.

295. Shi, J.; Deng, H.; Pan, H.; Xu, Y.; Zhang, M. Epigallocatechin-3-Gallate Attenuates Microcystin-LR Induced Oxidative Stress and Inflammation in Human Umbilical Vein Endothelial Cells. *Chemosphere* **2017**, *168*, 25–31. https://doi.org/10.1016/j.chemosphere.2016.10.037.

296. Luo, K. W.; Wei Chen; Lung, W. Y.; Wei, X. Y.; Cheng, B. H.; Cai, Z. M.; Huang, W. R. EGCG Inhibited Bladder Cancer SW780 Cell Proliferation and Migration Both in Vitro and in Vivo via Down-Regulation of NF-ΚB and MMP-9. *J. Nutr. Biochem.* **2017**, *41*, 56–64. https://doi.org/10.1016/j.jnutbio.2016.12.004.

297. Luo, K. W.; Lung, W. Y.; Chun-Xie; Luo, X. Le; Huang, W. R. EGCG Inhibited Bladder Cancer T24 and 5637 Cell Proliferation and Migration via PI3K/AKT Pathway. *Oncotarget* **2018**, *9* (15), 12261–12272. https://doi.org/10.18632/oncotarget.24301.

298. Zhang, Y.; Han, G.; Fan, B.; Zhou, Y.; Zhou, X.; Wei, L.; Zhang, J. Green Tea (-)-Epigallocatechin-3-Gallate down-Regulates VASP Expression and Inhibits Breast Cancer Cell Migration and Invasion by Attenuating Rac1 Activity. *Eur. J. Pharmacol.* **2009**, *606* (1–3), 172–179. https://doi.org/10.1016/j.ejphar.2008.12.033.

299. Yamakawa, S.; Asai, T.; Uchida, T.; Matsukawa, M.; Akizawa, T.; Oku, N. (-)-Epigallocatechin Gallate Inhibits Membrane-Type 1 Matrix Metalloproteinase, MT1-MMP, and Tumor Angiogenesis. *Cancer Lett.* **2004**, *210* (1), 47–55. https://doi.org/10.1016/j.canlet.2004.03.008.

300. Tudoran, O.; Soritau, O.; Balacescu, O.; Balacescu, L.; Braicu, C.; Rus, M.; Gherman, C.; Virag, P.; Irimie, F.; Berindan-Neagoe, I. Early Transcriptional Pattern of Angiogenesis Induced by EGCG Treatment in Cervical Tumour Cells. *J. Cell. Mol. Med.* **2012**, *16* (3), 520–530. https://doi.org/10.1111/j.1582-4934.2011.01346.x.

301. Annabi, B.; Lachambre, M. P.; Bousquet-Gagnon, N.; Pageé, M.; Gingras, D.; Beéliveau, R. Green Tea Polyphenol (-)-Epigallocatechin 3-Gallate Inhibits MMP-2 Secretion and MT1-MMP-Driven Migration in Glioblastoma Cells. *Biochim. Biophys. Acta - Mol. Cell Res.* **2002**, *1542* (1–3), 209–220. https://doi.org/10.1016/S0167-4889(01)00187-2.

302. Li, H.; Li, Z.; Xu, Y. M.; Wu, Y.; Yu, K. K.; Zhang, C.; Ji, Y. H.; Ding, G.; Chen, F. X. Epigallocatechin-3-Gallate Induces Apoptosis, Inhibits Proliferation and Decreases Invasion of Glioma Cell. *Neurosci. Bull.* **2014**, *30* (1), 67–73. https://doi.org/10.1007/s12264-013-1394-z.

303. Zhen, M. C.; Huang, X. H.; Wang, Q.; Sun, K.; Liu, Y. J.; Li, W.; Zhang, L. J.; Cao, L. Q.; Chen, X. L. Green Tea Polyphenol Epigallocatechin-3-Gallate Suppresses Rat Hepatic Stellate Cell Invasion by Inhibition of MMP-2 Expression and Its Activation. *Acta Pharmacol. Sin.* **2006**, *27* (12), 1600–1607. https://doi.org/10.1111/j.1745-7254.2006.00439.x.

304. Fassina, G.; Venè, R.; Morini, M.; Minghelli, S.; Benelli, R.; Noonan, D. M.; Albini, A. Mechanisms of Inhibition of Tumor Angiogenesis and Vascular Tumor Growth by Epigallocatechin-3-Gallate. *Clin. Cancer Res.* **2004**, *10* (14), 4865–4873. https://doi.org/10.1158/1078-0432.CCR-03-0672.

305. Li, X.; Feng, Y.; Liu, J.; Feng, X.; Zhou, K.; Tang, X. Epigallocatechin-3-Gallate Inhibits IGF-I-Stimulated Lung Cancer Angiogenesis through Downregulation of HIF-1α and VEGF Expression. *lifestyle Genomics* **2013**, *6* (3), 169–178. https://doi.org/10.1159/000354402.

306. Shi, J.; Liu, F.; Zhang, W.; Liu, X.; Lin, B.; Tang, X. Epigallocatechin-3-Gallate Inhibits Nicotine-Induced Migration and Invasion by the Suppression of Angiogenesis and Epithelial-Mesenchymal Transition in Non-Small Cell Lung Cancer Cells. *Oncol. Rep.* **2015**, *33* (6), 2972–2980. https://doi.org/10.3892/or.2015.3889.

307. Deng, Y. T.; Lin, J. K. EGCG Inhibits the Invasion of Highly Invasive CL1-5 Lung Cancer Cells through Suppressing MMP-2 Expression via JNK Signaling and Induces G2/M Arrest. *J. Agric. Food Chem.* **2011**, *59* (24), 13318–13327. https://doi.org/10.1021/jf204149c.

308. Pilorget, A.; Berthet, V.; Luis, J.; Moghrabi, A.; Annabi, B.; Béliveau, R. Medulloblastoma Cell Invasion Is Inhibited by Green Tea (-)Epigallocatechin-3-Gallate. *J. Cell. Biochem.* **2003**, *90* (4), 745–755. https://doi.org/10.1002/jcb.10667.

309. Ohga, N.; Hida, K.; Hida, Y.; Muraki, C.; Tsuchiya, K.; Matsuda, K.; Ohiro, Y.; Totsuka, Y.; Shindoh, M. Inhibitory Effects of Epigallocatechin-3 Gallate, a Polyphenol in Green Tea, on Tumor-Associated Endothelial Cells and Endothelial Progenitor Cells. *Cancer Sci.* **2009**, *100* (10), 1963–1970. https://doi.org/10.1111/j.1349-7006.2009.01255.x.

310. Lin, C. H.; Shen, Y. A.; Hung, P. H.; Yu, Y. Bin; Chen, Y. J. Epigallocathechin Gallate, Polyphenol Present in Green Tea, Inhibits Stem-like Characteristics and Epithelial-Mesenchymal Transition in Nasopharyngeal Cancer Cell Lines. *BMC Complement. Altern. Med.* **2012**, *12* (1), 1. https://doi.org/10.1186/1472-6882-12-201.

311. Fang, C. Y.; Wu, C. C.; Hsu, H. Y.; Chuang, H. Y.; Huang, S. Y.; Tsai, C. H.; Chang, Y.; Tsao, G. S. W.; Chen, C. L.; Chen, J. Y. EGCG Inhibits Proliferation, Invasiveness and Tumor Growth by up-Regulation of Adhesion Molecules, Suppression of Gelatinases Activity, and Induction of Apoptosis in Nasopharyngeal Carcinoma Cells. *Int. J. Mol. Sci.* **2015**, *16* (2), 2530–2558. https://doi.org/10.3390/ijms16022530.

312. Lin, C. H.; Wang, H. H.; Chen, T. H.; Chiang, M. C.; Hung, P. H.; Chen, Y. J. Involvement of Microrna-296 in the Inhibitory Effect of Epigallocatechin Gallate against the Migratory Properties of Anoikis-Resistant Nasopharyngeal Carcinoma Cells. *Cancers (Basel).* **2020**, *12* (4), 973. https://doi.org/10.3390/cancers12040973.

313. Hossain, M. M.; Banik, N. L.; Ray, S. K. Survivin Knockdown Increased Anti-Cancer Effects of (-)-Epigallocatechin-3-Gallate in Human Malignant Neuroblastoma SK-N-BE2 and SH-SY5Y Cells. *Exp. Cell Res.* **2012**, *318* (13), 1597–1610. https://doi.org/10.1016/j.yexcr.2012.03.033.

314. Garbisa, S.; Sartor, L.; Biggin, S.; Salvato, B.; Benelli, R.; Albini, A. Tumor Gelatinases and Invasion Inhibited by the Green Tea Flavanol Epigallocatechin-3-Gallate. *Cancer* **2001**, *91* (4), 822–832. https://doi.org/10.1002/1097-0142(20010215)91:4<822::AID-CNCR1070>3.0.CO;2-G.

315. Ho, Y. C.; Yang, S. F.; Peng, C. Y.; Chou, M. Y.; Chang, Y. C. Epigallocatechin-3-Gallate Inhibits the Invasion of Human Oral Cancer Cells and Decreases the Productions of Matrix Metalloproteinases and Urokinase-Plasminogen Activator. *J. Oral Pathol. Med.* **2007**, *36* (10), 588–593. https://doi.org/10.1111/j.1600-0714.2007.00588.x.

316. Chen, P. N.; Chu, S. C.; Kuo, W. H.; Chou, M. Y.; Lin, J. K.; Hsieh, Y. S. Epigallocatechin-3 Gallate Inhibits Invasion, Epithelial-Mesenchymal Transition, and Tumor Growth in Oral Cancer Cells. *J. Agric. Food Chem.* **2011**, *59* (8), 3836–3844. https://doi.org/10.1021/jf1049408.

317. Kawabata, T.; Otsuka, T.; Fujita, K.; Sakai, G.; Matsushima-Nishiwaki, R.; Kozawa, O.; Tokuda, H. (-)-Epigallocatechin Gallate but Not Chlorogenic Acid Suppresses EGF-Stimulated Migration of Osteoblasts via Attenuation of P38 MAPK Activity. *Int. J. Mol. Med.* **2018**, *42* (6), 3149–3156. https://doi.org/10.3892/ijmm.2018.3884.

318. Kawabata, T.; Tokuda, H.; Sakai, G.; Fujita, K.; Matsushima-Nishiwaki, R.; Otsuka, T.; Kozawa, O. Repression of IGF-I-Induced Osteoblast Migration by (-)-Epigallocatechin Gallate through P44/P42 MAP Kinase Signaling. *Biomed. Reports* **2018**, *9* (4), 318–326. https://doi.org/10.3892/br.2018.1140.

319. Spinella, F.; Rosanò, L.; Di Castro, V.; Decandia, S.; Albini, A.; Nicotra, M. R.; Natali, P. G.; Bagnato, A. Green Tea Polyphenol Epigallocatechin-3-Gallate Inhibits the Endothelin Axis and Downstream Signaling Pathways in Ovarian Carcinoma. *Mol. Cancer Ther.* **2006**, *5* (6), 1483–1492. https://doi.org/10.1158/1535-7163.MCT-06-0053.

320. Masamune, A.; Kikuta, K.; Satoh, M.; Suzuki, N.; Shimosegawa, T. Green Tea Polyphenol Epigallocatechin-3-Gallate Blocks PDGF-Induced Proliferation and Migration of Rat Pancreatic Stellate Cells. *World J. Gastroenterol.* **2005**, *11* (22), 3368–3374. https://doi.org/10.3748/wjg.v11.i22.3368.

321. Duhon, D.; Bigelow, R. L. H.; Coleman, D. T.; Steffan, J. J.; Yu, C.; Langston, W.; Kevil, C. G.; Cardelli, J. A. The Polyphenol Epigallocatechin-3-Gallate Affects Lipid Rafts to Block Activation of the c-Met Receptor in Prostate Cancer Cells. *Mol. Carcinog.* **2010**, *49* (8), 739–749. https://doi.org/10.1002/mc.20649.

322. Chan, C. M.; Huang, J. H.; Chiang, H. S.; Wu, W. Bin; Lin, H. H.; Hong, J. Y.; Hung, C. F. Effects of (-)-Epigallocatechin Gallate on Rpe Cell Migration and Adhesion. *Mol. Vis.* **2010**, *16* (510), 586–595.

323. Lee, H. S.; Jun, J. H.; Jung, E. H.; Koo, B. A.; Kim, Y. S. Epigalloccatechin-3-Gallate Inhibits Ocular Neovascularization and Vascular Permeability in Human Retinal Pigment Epithelial and Human Retinal Microvascular Endothelial Cells via Suppression of MMP-9 and VEGF Activation. *Molecules* **2014**, *19* (8), 12150–12172. https://doi.org/10.3390/molecules190812150.

324. Appleton, K. J. M. I. Epicatechin Gallate Improves Healing and Reduces Scar Formation of Incisional Wounds in Type 2 Diabetes Mellitus Rat Model. *Wounds* **2012**, *24* (3), 55–57.

325. Huang, S. F.; Horng, C. T.; Hsieh, Y. S.; Hsieh, Y. H.; Chu, S. C.; Chen, P. N. Epicatechin-3-Gallate Reverses TGF-Β1-Induced Epithelial-to-Mesenchymal Transition and Inhibits Cell Invasion and Protease Activities in Human Lung Cancer Cells. *Food Chem. Toxicol.* **2016**, *94*, 1–10. https://doi.org/10.1016/j.fct.2016.05.009.

326. Jiang, C.; Agarwal, R.; Lü, J. Anti-Angiogenic Potential of a Cancer Chemopreventive Flavonoid Antioxidant, Silymarin: Inhibition of Key Attributes of Vascular Endothelial Cells and Angiogenic Cytokine Secretion by Cancer Epithelial Cells. *Biochem. Biophys. Res. Commun.* **2000**, *276* (1), 371–378. https://doi.org/10.1006/bbrc.2000.3474.

327. Ting, H.; Deep, G.; Agarwal, R. Molecular Mechanisms of Silibinin-Mediated Cancer Chemoprevention with Major Emphasis on Prostate Cancer. *AAPS J.* **2013**, *15* (3), 707–716. https://doi.org/10.1208/s12248-013-9486-2.

328. Wu, K.; Ning, Z.; Zeng, J.; Fan, J.; Zhou, J.; Zhang, T.; Zhang, L.; Chen, Y.; Gao, Y.; Wang, B.; Guo, P.; Li, L.; Wang, X.; He, D. Silibinin Inhibits β-Catenin/ZEB1 Signaling and Suppresses Bladder Cancer Metastasis via Dual-Blocking Epithelial-Mesenchymal Transition and Stemness. *Cell. Signal.* **2013**, *25* (12), 2625–2633. https://doi.org/10.1016/j.cellsig.2013.08.028.

329. Li, F.; Sun, Y.; Jia, J.; Yang, C.; Tang, X.; Jin, B.; Wang, K.; Guo, P.; Ma, Z.; Chen, Y.; Wang, X.; Chang, L.; He, D.; Zeng, J. Silibinin Attenuates TGF-Β1-Induced Migration and Invasion via EMT Suppression and Is Associated with COX-2 Downregulation in Bladder Transitional Cell Carcinoma. *Oncol. Rep.* **2018**, *40* (6), 3543–3550. https://doi.org/10.3892/or.2018.6728.

330. Dastpeyman, M.; Motamed, N.; Azadmanesh, K.; Mostafavi, E.; Kia, V.; Jahanian-Najafabadi, A.; Shokrgozar, M. A. Inhibition of Silibinin on Migration and Adhesion Capacity of Human Highly Metastatic Breast Cancer Cell Line, MDA-MB-231, by Evaluation of Β1-Integrin and Downstream Molecules, Cdc42, Raf-1 and D4GDI. *Med. Oncol.* **2012**, *29* (4), 2512–2518. https://doi.org/10.1007/s12032-011-0113-8.

331. Byun, H. J.; Darvin, P.; Kang, D. Y.; Sp, N.; Joung, Y. H.; Park, J. H.; Kim, S. J.; Yang, Y. M. Silibinin Downregulates MMP2 Expression via Jak2/STAT3 Pathway and Inhibits the Migration and Invasive Potential in MDA-MB-231 Cells. *Oncol. Rep.* **2017**, *37* (6), 3270–3278. https://doi.org/10.3892/or.2017.5588.

332. Lin, C. M.; Chen, Y. H.; Ma, H. P.; Wang, B. W.; Chiu, J. H.; Chua, S. K.; Ong, J. R.; Shyu, K. G. Silibinin Inhibits the Invasion of IL-6-Stimulated Colon Cancer Cells via Selective JNK/AP-1/MMP-2 Modulation in Vitro. *J. Agric. Food Chem.* **2012**, *60* (51), 12451–12457. https://doi.org/10.1021/jf300964f.

333. Kim, K. W.; Choi, C. H.; Kim, T. H.; Kwon, C. H.; Woo, J. S.; Kim, Y. K. Silibinin Inhibits Glioma Cell Proliferation via Ca2+/Ros/Mapk- Dependent Mechanism in Vitro and Glioma Tumor Growth in Vivo. *Neurochem. Res.* **2009**, *34* (8), 1479–1490. https://doi.org/10.1007/s11064-009-9935-6.

334. Deep, G.; Kumar, R.; Jain, A. K.; Agarwal, C.; Agarwal, R. Silibinin Inhibits Fibronectin Induced Motility, Invasiveness and Survival in Human Prostate Carcinoma PC3 Cells via Targeting Integrin Signaling. *Mutat. Res. - Fundam. Mol. Mech. Mutagen.* **2014**, *768* (C), 35–46. https://doi.org/10.1016/j.mrfmmm.2014.05.002.

335. Deep, G.; Kumar, R.; Nambiar, D. K.; Jain, A. K.; Ramteke, A. M.; Serkova, N. J.; Agarwal, C.; Agarwal, R. Silibinin Inhibits Hypoxia-Induced HIF-1α-Mediated Signaling, Angiogenesis and Lipogenesis in Prostate Cancer Cells: In Vitro Evidence and in Vivo Functional Imaging and Metabolomics. *Mol. Carcinog.* **2017**, *56* (3), 833–848. https://doi.org/10.1002/mc.22537.

336. Mokhtari, M. J.; Motamed, N.; Shokrgozar, M. A. Evaluation of Silibinin on the Viability, Migration and Adhesion of the Human Prostate Adenocarcinoma (PC-3) Cell Line. *Cell Biol. Int.* **2008**, *32* (8), 888–892. https://doi.org/10.1016/j.cellbi.2008.03.019.

337. Liang, L.; Li, L.; Zeng, J.; Gao, Y.; Chen, Y. Le; Wang, Z. Q.; Wang, X. Y.; Chang, L. S.; He, D. Inhibitory Effect of Silibinin on EGFR Signal-Induced Renal Cell Carcinoma Progression via Suppression of the EGFR/MMP-9 Signaling Pathway. *Oncol. Rep.* **2012**, *28* (3), 999–1005. https://doi.org/10.3892/or.2012.1874.

338. Lin, C. H.; Li, C. H.; Liao, P. L.; Tse, L. S.; Huang, W. K.; Cheng, H. W.; Cheng, Y. W. Silibinin Inhibits VEGF Secretion and Age-Related Macular Degeneration in a Hypoxia-Dependent Manner through the PI-3 Kinase/Akt/MTOR Pathway. *Br. J. Pharmacol.* **2013**, *168* (4), 920–931. https://doi.org/10.1111/j.1476-5381.2012.02227.x.

339. Chen, X.; Gu, N.; Xue, C.; Li, B. R. Plant Flavonoid Taxifolin Inhibits the Growth, Migration and Invasion of Human Osteosarcoma Cells. *Mol. Med. Rep.* **2018**, *17* (2), 3239–3245. https://doi.org/10.3892/mmr.2017.8271.

340. Maués, L. A. L.; Alves, G. M.; Couto, N. M. G.; da Silva, B. J. M.; Arruda, M. S. P.; Macchi, B. M.; Sena, C. B. C.; Prado, A. F.; Crespo-Lopez, M. E.; Silva, E. O.; do Nascimento, J. L. M. Flavonoids from the Amazon Plant Brosimum Acutifolium Induce C6 Glioma Cell Line Apoptosis by Disrupting Mitochondrial Membrane Potential and Reducing AKT Phosphorylation. *Biomed. Pharmacother.* **2019**, *113*, 108728. https://doi.org/10.1016/j.biopha.2019.108728.

341. Gao, M.; Chang, Y.; Wang, X.; Ban, C.; Zhang, F. Reduction of COX-2 through Modulating MiR-124/ SPHK1 Axis Contributes to the Antimetastatic Effect of Alpinumisoflavone in Melanoma. *Am. J. Transl. Res.* **2017**, *9* (3), 986–998.

342. Wang, Y.; Li, J. J.; Chen, Y. M. Biochanin A Extirpates the Epithelial-Mesenchymal Transition in a Human Lung Cancer. *Exp. Ther. Med.* **2018**, *15* (3), 2830–2836. https://doi.org/10.3892/etm.2018.5731.

343. Chen, C. Y.; Chen, C. C.; Shieh, T. M.; Hsueh, C.; Wang, S. H.; Leu, Y. L.; Lian, J. H.; Wang, T. H. Corylin Suppresses Hepatocellular Carcinoma Progression via the Inhibition of Epithelial-Mesenchymal Transition, Mediated by Long Noncoding RNA GAS5. *Int. J. Mol. Sci.* **2018**, *19* (2), 1–13. https://doi.org/10.3390/ijms19020380.

344. Sophors, P.; Kim, Y. M.; Seo, G. Y.; Huh, J. S.; Lim, Y.; Koh, D. S.; Cho, M. A Synthetic Isoflavone, DCMF, Promotes Human Keratinocyte Migration by Activating Src/FAK Signaling Pathway. *Biochem. Biophys. Res. Commun.* **2016**, *472* (2), 332–338. https://doi.org/10.1016/j.bbrc.2016.02.106.

345. Zhao, J.; Xu, J.; Lv, J. Identification of Profilin 1 as the Primary Target for the Anti-Cancer Activities of Furowanin A in Colorectal Cancer. *Pharmacol. Reports* **2019**, *71* (5), 940–949. https://doi.org/10.1016/j.pharep.2019.05.007.

346. Pavese, J. M.; Farmer, R. L.; Bergan, R. C. Inhibition of Cancer Cell Invasion and Metastasis by Genistein. *Cancer Metastasis Rev.* **2010**, *29* (3), 465–482. https://doi.org/10.1007/s10555-010-9238-z.

347. Shao, Z.; Wu, J.; Shen, Z. Genistein Exerts Multiple Suppressive Effects on Human Breast Carcinoma Cells. *Zhonghua Zhong Liu Za Zhi* **2000**, *22* (5), 362–365.

348. Zhang, Q.; Bao, J.; Yang, J. Genistein-Triggered Anticancer Activity against Liver Cancer Cell Line HepG2 Involves ROS Generation, Mitochondrial Apoptosis, G2/M Cell Cycle Arrest and Inhibition of Cell Migrationand Inhibition of Cell Migration. *Arch. Med. Sci.* **2019**, *15* (4), 1001–1009. https://doi.org/10.5114/aoms.2018.78742.

349. Panda, S. P.; Panigrahy, U. P.; Prasanth, D. S. N. B. K.; Gorla, U. S.; Guntupalli, C.; Panda, D. P.; Jena, B. R. A Trimethoxy Flavonoid Isolated from Stem Extract of Tabebuia Chrysantha Suppresses Angiogenesis in Angiosarcoma. *J. Pharm. Pharmacol.* **2020**, *72*, 990–999. https://doi.org/10.1111/jphp.13272.

350. Bui, N. T.; Ho, M. T.; Kim, Y. M.; Lim, Y.; Cho, M. Flavonoids Promoting HaCaT Migration: II. Molecular Mechanism of 4′,6,7-Trimethoxyisoflavone via NOX2 Activation. *Phytomedicine* **2014**, *21* (4), 570–577. https://doi.org/10.1016/j.phymed.2013.10.010.

351. WANG, Y.; MA, W.; ZHENG, W. Deguelin, a Novel Anti-Tumorigenic Agent Targeting Apoptosis, Cell Cycle Arrest and Anti-Angiogenesis for Cancer Chemoprevention. *Mol. Clin. Oncol.* **2013**, *1* (2), 215–219. https://doi.org/10.3892/mco.2012.36.

352. Dell’Eva, R.; Minghelli, S.; Minghelli, S.; Noonan, D. M.; Albini, A.; Ferrari, N. The Akt Inhibitor Deguelin, Is an Angiopreventive Agent Also Acting on the NF-ΚB Pathway. *Carcinogenesis* **2007**, *28* (2), 404–413. https://doi.org/10.1093/carcin/bgl162.

353. Zheng, W.; Lu, S.; Cai, H.; Kang, M.; Qin, W.; Li, C.; Wu, Y. Deguelin Inhibits Proliferation and Migration of Human Pancreatic Cancer Cells in Vitro Targeting Hedgehog Pathway. *Oncol. Lett.* **2016**, *12* (4), 2761–2765. https://doi.org/10.3892/ol.2016.4928.

354. Hsu, Y. L.; Wu, L. Y.; Hou, M. F.; Tsai, E. M.; Lee, J. N.; Liang, H. L.; Jong, Y. J.; Hung, C. H.; Kuo, P. L. Glabridin, an Isoflavan from Licorice Root, Inhibits Migration, Invasion and Angiogenesis of MDA-MB-231 Human Breast Adenocarcinoma Cells by Inhibiting Focal Adhesion Kinase/Rho Signaling Pathway. *Mol. Nutr. Food Res.* **2011**, *55* (2), 318–327. https://doi.org/10.1002/mnfr.201000148.

355. Tsai, Y. M.; Yang, C. J.; Hsu, Y. L.; Wu, L. Y.; Tsai, Y. C.; Hung, J. Y.; Lien, C. T.; Huang, M. S.; Kuo, P. L. Glabridin Inhibits Migration, Invasion, and Angiogenesis of Human Non-Small Cell Lung Cancer A549 Cells by Inhibiting the FAK/Rho Signaling Pathway. *Integr. Cancer Ther.* **2011**, *10* (4), 341–349. https://doi.org/10.1177/1534735410384860.

356. Nozomu Matsunaga, Kazuhiro Tsuruma, Masamitsu Shimazawa, Shigeru Yokota and Hara, H. Inhibitory Actions of Bilberry Anthocyanidins on Angiogenesis. *Phyther. Res.* **2010**, *24*, S42–S47. https://doi.org/10.1002/ptr.

357. Patel, K.; Jain, A.; Patel, D. K. Medicinal Significance, Pharmacological Activities, and Analytical Aspects of Anthocyanidins ‘Delphinidin’: A Concise Report. *J. Acute Dis.* **2013**, *2* (3), 169–178. https://doi.org/10.1016/s2221-6189(13)60123-7.

358. Lamy, S.; Blanchette, M.; Michaud-Levesque, J.; Lafleur, R.; Durocher, Y.; Moghrabi, A.; Barrette, S.; Gingras, D.; Béliveau, R. Delphinidin, a Dietary Anthocyanidin, Inhibits Vascular Endothelial Growth Factor Receptor-2 Phosphorylation. *Carcinogenesis* **2006**, *27* (5), 989–996. https://doi.org/10.1093/carcin/bgi279.

359. Favot, L.; Martin, S.; Keravis, T.; Andriantsitohaina, R.; Lugnier, C. Involvement of Cyclin-Dependent Pathway in the Inhibitory Effect of Delphinidin on Angiogenesis. *Cardiovasc. Res.* **2003**, *59* (2), 479–487. https://doi.org/10.1016/S0008-6363(03)00433-4.

360. Viegas, O.; Faria, M. A.; Sousa, J. B.; Vojtek, M.; Gonçalves-Monteiro, S.; Suliburska, J.; Diniz, C.; Ferreira, I. M. P. L. V. O. Delphinidin-3-O-Glucoside Inhibits Angiogenesis via VEGFR2 Downregulation and Migration through Actin Disruption. *J. Funct. Foods* **2019**, *54*, 393–402. https://doi.org/10.1016/j.jff.2019.01.039.

361. Huang, C. C.; Hung, C. H.; Hung, T. W.; Lin, Y. C.; Wang, C. J.; Kao, S. H. Dietary Delphinidin Inhibits Human Colorectal Cancer Metastasis Associating with Upregulation of MiR-204-3p and Suppression of the Integrin/FAK Axis. *Sci. Rep.* **2019**, *9* (1), 1–11. https://doi.org/10.1038/s41598-019-55505-z.

362. Kim, M. H.; Jeong, Y. J.; Cho, H. J.; Hoe, H. S.; Park, K. K.; Park, Y. Y.; Choi, Y. H.; Kim, C. H.; Chang, H. W.; Park, Y. J.; Chung, I. K.; Chang, Y. C. Delphinidin Inhibits Angiogenesis through the Suppression of HIF-1α and VEGF Expression in A549 Lung Cancer Cells. *Oncol. Rep.* **2017**, *37* (2), 777–784. https://doi.org/10.3892/or.2016.5296.

363. Kang, H. M.; Park, B. S.; Kang, H. K.; Park, H. R.; Yu, S. Bin; Kim, I. R. Delphinidin Induces Apoptosis and Inhibits Epithelial-to-Mesenchymal Transition via the ERK/P38 MAPK-Signaling Pathway in Human Osteosarcoma Cell Lines. *Environ. Toxicol.* **2018**, *33* (6), 640–649. https://doi.org/10.1002/tox.22548.

364. Morbidelli, L. Polyphenol-Based Nutraceuticals for the Control of Angiogenesis: Analysis of the Critical Issues for Human Use. *Pharmacol. Res.* **2016**, *111*, 384–393. https://doi.org/10.1016/j.phrs.2016.07.011.

365. Mirossay, L.; Varinská, L.; Mojžiš, J. Antiangiogenic Effect of Flavonoids and Chalcones: An Update. *Int. J. Mol. Sci.* **2018**, *19* (1), 27. https://doi.org/10.3390/ijms19010027.

366. Dulak, J. Nutraceuticals as Anti-Angiogenic Agents: Hopes and Reality. *J. Physiol. Pharmacol.* **2005**, *56* (1), 51–67.

367. Diniz, C.; Suliburska, J.; Ferreira, I. M. New Insights into the Antiangiogenic and Proangiogenic Properties of Dietary Polyphenols. *Mol. Nutr. Food Res.* **2017**, *61* (6), 1–17. https://doi.org/10.1002/mnfr.201600912.

368. Kapinova, A.; Stefanicka, P.; Kubatka, P.; Zubor, P.; Uramova, S.; Kello, M.; Mojzis, J.; Blahutova, D.; Qaradakhi, T.; Zulli, A.; Caprnda, M.; Danko, J.; Lasabova, Z.; Busselberg, D.; Kruzliak, P. Are Plant-Based Functional Foods Better Choice against Cancer than Single Phytochemicals? A Critical Review of Current Breast Cancer Research. *Biomed. Pharmacother.* **2017**, *96*, 1465–1477. https://doi.org/10.1016/j.biopha.2017.11.134.

369. Mojzis, J.; Varinska, L.; Mojzisova, G.; Kostova, I.; Mirossay, L. Antiangiogenic Effects of Flavonoids and Chalcones. *Pharmacol. Res.* **2008**, *57* (4), 259–265. https://doi.org/10.1016/j.phrs.2008.02.005.

370. Ravishankar, D.; Rajora, A. K.; Greco, F.; Osborn, H. M. I. Flavonoids as Prospective Compounds for Anti-Cancer Therapy. *Int. J. Biochem. Cell Biol.* **2013**, *45* (12), 2821–2831. https://doi.org/10.1016/j.biocel.2013.10.004Review.

371. Cao, Y.; Cao, R.; Bråkenhielm, E. Antiangiogenic Mechanisms of Diet-Derived Polyphenols. *J. Nutr. Biochem.* **2002**, *13* (7), 380–390. https://doi.org/10.1016/S0955-2863(02)00204-8.

372. Le Marchand, L. Cancer Preventive Effects of Flavonoids - A Review. *Biomed. Pharmacother.* **2002**, *56* (6), 296–301. https://doi.org/10.1016/S0753-3322(02)00186-5.

373. Nandakumar, V.; Singh, T.; Katiyar, S. K. Multi-Targeted Prevention and Therapy of Cancer by Proanthocyanidins. *Cancer Lett.* **2008**, *269* (2), 378–387. https://doi.org/10.1016/j.canlet.2008.03.049.

374. Martin, M. A.; Goya, L.; Ramos, S. Potential for Preventive Effects of Cocoa and Cocoa Polyphenols in Cancer. *Food Chem. Toxicol.* **2013**, *56*, 336–351. https://doi.org/10.1016/j.fct.2013.02.020.

375. Zhang, J. P.; Tian, X. H.; Yang, Y. X.; Liu, Q. X.; Wang, Q.; Chen, L. P.; Li, H. L.; Zhang, W. D. Gleditsia Species: An Ethnomedical, Phytochemical and Pharmacological Review. *J. Ethnopharmacol.* **2016**, *178*, 155–171. https://doi.org/10.1016/j.jep.2015.11.044.

376. Mirza-Aghazadeh-Attari, M.; Ekrami, E. M.; Aghdas, S. A. M.; Mihanfar, A.; Hallaj, S.; Yousefi, B.; Safa, A.; Majidinia, M. Targeting PI3K/Akt/MTOR Signaling Pathway by Polyphenols: Implication for Cancer Therapy. *Life Sci.* **2020**, 117481. https://doi.org/10.1016/j.lfs.2020.117481.

377. Kang, N. J.; Shin, S. H.; Lee, H. J.; Lee, K. W. Polyphenols as Small Molecular Inhibitors of Signaling Cascades in Carcinogenesis. *Pharmacol. Ther.* **2011**, *130* (3), 310–324. https://doi.org/10.1016/j.pharmthera.2011.02.004.

378. Hussain, S. S.; Kumar, A. P.; Ghosh, R. Food-Based Natural Products for Cancer Management: Is the Whole Greater than the Sum of the Parts? *Semin. Cancer Biol.* **2016**, *40*, 233–246. https://doi.org/10.1016/j.semcancer.2016.06.002.

379. Lewandowska, H.; Kalinowska, M.; Lewandowski, W.; Stepkowski, T. M.; Brzóska, K. The Role of Natural Polyphenols in Cell Signaling and Cytoprotection against Cancer Development. *J. Nutr. Biochem.* **2016**, *32*, 1–19. https://doi.org/10.1016/j.jnutbio.2015.11.006.

380. Lai, C. S.; Pan, M. H. Mechanism for Possible Chemopreventive Effects of Natural Dietary Compounds on Smoking-Induced Tumorigenesis. *J. Exp. Clin. Med.* **2011**, *3* (6), 262–271. https://doi.org/10.1016/j.jecm.2011.10.003.

381. Kanadaswami, C.; Lee, L. T.; Lee, P. P. H.; Hwang, J. J.; Ke, F. C.; Huang, Y. T.; Lee, M. T. The Antitumor Activities of Flavonoids. *In Vivo (Brooklyn).* **2005**, *19* (5), 895–910.

382. Stoner, G. D.; Wang, L. S.; Casto, B. C. Laboratory and Clinical Studies of Cancer Chemoprevention by Antioxidants in Berries. *Carcinogenesis* **2008**, *29* (9), 1665–1674. https://doi.org/10.1093/carcin/bgn142.

383. Kaur, J.; Kaur, G. An Insight into the Role of Citrus Bioactives in Modulation of Colon Cancer. *J. Funct. Foods* **2015**, *13*, 239–261. https://doi.org/10.1016/j.jff.2014.12.043.

384. Araújo, J. R.; Gonçalves, P.; Martel, F. Chemopreventive Effect of Dietary Polyphenols in Colorectal Cancer Cell Lines. *Nutr. Res.* **2011**, *31* (2), 77–87. https://doi.org/10.1016/j.nutres.2011.01.006.

385. Xu, Z.; Sun, T.; Li, W.; Sun, X. Inhibiting Effects of Dietary Polyphenols on Chronic Eye Diseases. *J. Funct. Foods* **2017**, *39*, 186–197. https://doi.org/10.1016/j.jff.2017.10.031.

386. Stagos, D.; Amoutzias, G. D.; Matakos, A.; Spyrou, A.; Tsatsakis, A. M.; Kouretas, D. Chemoprevention of Liver Cancer by Plant Polyphenols. *Food Chem. Toxicol.* **2012**, *50* (6), 2155–2170. https://doi.org/10.1016/j.fct.2012.04.002.

387. García, E. R.; Gutierrez, E. A.; Melo, F. C. S. A. De; Novaes, R. D.; Gonçalves, R. V. Flavonoids Effects on Hepatocellular Carcinoma in Murine Models: A Systematic Review. *Evidence-based Complement. Altern. Med.* **2018**, *2018*. https://doi.org/10.1155/2018/6328970.

388. Pojero, F.; Poma, P.; Spanò, V.; Montalbano, A.; Barraja, P.; Notarbartolo, M. Targeting Multiple Myeloma with Natural Polyphenols. *Eur. J. Med. Chem.* **2019**, *180*, 465–485. https://doi.org/10.1016/j.ejmech.2019.07.041.

389. Sulaiman, R. S.; Basavarajappa, H. D.; Corson, T. W. Natural Product Inhibitors of Ocular Angiogenesis. *Exp. Eye Res.* **2014**, *129*, 161–171. https://doi.org/10.1016/j.exer.2014.10.002.

390. Rajesh, G.; Harshala, S.; Dhananjay, G.; Jadhav, A.; Vikram, G. Effect of Hydroxyl Substitution of Flavone on Angiogenesis and Free Radical Scavenging Activities: A Structure-Activity Relationship Studies Using Computational Tools. *Eur. J. Pharm. Sci.* **2010**, *39* (1–3), 37–44. https://doi.org/10.1016/j.ejps.2009.10.008.

391. Lam, I. K.; Alex, D.; Wang, Y. H.; Liu, P.; Liu, A. L.; Du, G. H.; Yuen Lee, S. M. In Vitro and in Vivo Structure and Activity Relationship Analysis of Polymethoxylated Flavonoids: Identifying Sinensetin as a Novel Antiangiogenesis Agent. *Mol. Nutr. Food Res.* **2012**, *56* (6), 945–956. https://doi.org/10.1002/mnfr.201100680.

392. Fotsis, T.; Pepper, M. S.; Aktas, E.; Breit, S.; Rasku, S.; Adlercreutz, H.; Wähälä, K.; Montesano, R.; Schweigerer, L. Flavonoids, Dietary-Derived Inhibitors of Cell Proliferation and in Vitro Angiogenesis. *Cancer Res.* **1997**, *57* (14), 2916–2921.

393. Suktap, C.; Lee, H. K.; Amnuaypol, S.; Suttisri, R.; Sukrong, S. Wound Healing Effect of Flavonoid Glycosides from Afgekia Mahidolae B.L.Burtt & Chermsir. Leaves. *Rec. Nat. Prod.* **2018**, *12* (4), 391–396. https://doi.org/10.25135/rnp.39.17.10.166.

394. Gacche, R. N.; Meshram, R. J.; Shegokar, H. D.; Gond, D. S.; Kamble, S. S.; Dhabadge, V. N.; Utage, B. G.; Patil, K. K.; More, R. A. Flavonoids as a Scaffold for Development of Novel Anti-Angiogenic Agents: An Experimental and Computational Enquiry. *Arch. Biochem. Biophys.* **2015**, *577*–*578*, 35–48. https://doi.org/10.1016/j.abb.2015.04.009.

395. Zhang, J.; Wu, Y.; Zhao, X.; Luo, F.; Li, X.; Zhu, H.; Sun, C.; Chen, K. Chemopreventive Effect of Flavonoids from Ougan (Citrus Reticulata Cv. Suavissima) Fruit against Cancer Cell Proliferation and Migration. *J. Funct. Foods* **2014**, *10*, 511–519. https://doi.org/10.1016/j.jff.2014.08.006.

396. Gacche, R. N.; Shegokar, H. D.; Gond, D. S.; Yang, Z.; Jadhav, A. D. Evaluation of Selected Flavonoids as Antiangiogenic, Anticancer, and Radical Scavenging Agents: An Experimental and In Silico Analysis. *Cell Biochem. Biophys.* **2011**, *61* (3), 651–663. https://doi.org/10.1007/s12013-011-9251-z.

397. Santos, B. L.; Oliveira, M. N.; Coelho, P. L. C.; Pitanga, B. P. S.; Da Silva, A. B.; Adelita, T.; Silva, V. D. A.; Costa, M. D. F. D.; El-Bachá, R. S.; Tardy, M.; Chneiweiss, H.; Junier, M. P.; Moura-Neto, V.; Costa, S. L. Flavonoids Suppress Human Glioblastoma Cell Growth by Inhibiting Cell Metabolism, Migration, and by Regulating Extracellular Matrix Proteins and Metalloproteinases Expression. *Chem. Biol. Interact.* **2015**, *242*, 123–138. https://doi.org/10.1016/j.cbi.2015.07.014.

398. Ouanouki, A.; Lamy, S.; Annabi, B. Anthocyanidins Inhibit Epithelial–Mesenchymal Transition through a TGFβ/Smad2 Signaling Pathway in Glioblastoma Cells. *Mol. Carcinog.* **2017**, *56* (3), 1088–1099. https://doi.org/10.1002/mc.22575.

399. Li, N.; Zhang, P.; Wu, H.; Wang, J.; Liu, F.; Wang, W. Natural Flavonoids Function as Chemopreventive Agents from Gancao ( Glycyrrhiza Inflata Batal ). *J. Funct. Foods* **2015**, *19*, 563–574. https://doi.org/10.1016/j.jff.2015.09.045.

400. Wang, C. G.; Yao, W. N.; Zhang, B.; Hua, J.; Liang, D.; Wang, H. S. Lung Cancer and Matrix Metalloproteinases Inhibitors of Polyphenols from Selaginella Tamariscina with Suppression Activity of Migration. *Bioorganic Med. Chem. Lett.* **2018**, *28* (14), 2413–2417. https://doi.org/10.1016/j.bmcl.2018.06.024.

401. Hsiao, Y. C.; Kuo, W. H.; Chen, P. N.; Chang, H. R.; Lin, T. H.; Yang, W. E.; Hsieh, Y. S.; Chu, S. C. Flavanone and 2′-OH Flavanone Inhibit Metastasis of Lung Cancer Cells via down-Regulation of Proteinases Activities and MAPK Pathway. *Chem. Biol. Interact.* **2007**, *167* (3), 193–206. https://doi.org/10.1016/j.cbi.2007.02.012.
