# Supplementary material for: Antiangiogenic Activity of Flavonoids: A Systematic Review and Meta-Analysis
Source: Molecules. 2020 Oct 14;25(20):4712. doi: 10.3390/molecules25204712 (PMC7594036; doi:10.3390/molecules25204712)
Supplement: Supplementary file 1 [file molecules-25-04712-s001.zip › Table S5.docx]

**Table 5.** Search strategy used on ScienceDirect, PubMed and Web of Science electronic databases.

| **Database** | **Search Terms** | **Field** | **Limited to** | **Number of Items found** |
| --- | --- | --- | --- | --- |
| **ScienceDirect** | All possible combinations of 1 keyword from (flavonoid, flavone, flavonol, flavanol, anthocyanidin, polyphenol)  AND  1 keyword from (angiogenesis, antiangiogenic, proangiogenic, (cell migration), (wound healing)) | Title, abstract or author-specified keywords | Research articles, review articles, mini reviews and short communications | 1277 |
| **PubMed** |  | Title/Abstract | Journal articles, meta-analysis, review and systematic reviews | 1984 |
| **Web of Science** |  | Title | Article, review | 119 |
|  |  |  |  | Total = 3380 |
