# Supplementary material for: Antiangiogenic Activity of Flavonoids: A Systematic Review and Meta-Analysis
Source: Molecules. 2020 Oct 14;25(20):4712. doi: 10.3390/molecules25204712 (PMC7594036; doi:10.3390/molecules25204712)
Supplement: Supplementary file 1 [file molecules-25-04712-s001.zip › Tables S2-S4.docx]

**Table 2.** Low concentration range (10 -20 µM) sensitivity analysis.

| Subgroup | Before Overall summary (95%CI X - Y, *I^2^*) | After Overall summary (95%CI X - Y, *I^2^*) | Left-out-study |
| --- | --- | --- | --- |
| Isoflavones | 0.71 (0.66 - 0.76, 99%) | 0.73 (0.71 – 0.76, 93%)­­ | Gacche 2011 (Genistein) |
|  |  | 0.70 (0.62 – 0.80, 99%) | Gacche 2015 (Biochanin-A) |
|  |  | 0.69 (0.63 – 0.75, 98%) | Gacche 2015 (Formononetin) |
| Flavones | 0.73 (0.70 – 0.77, 98%) | 0.73 (0.69 – 0.76, 98%) | Gacche 2010 (5-Hydroxyflavone) |
|  |  | 0.72 (0.69 – 0.76, 97%) | Gacche 2010 (6-Hydroxyflavone) |
|  |  | 0.73 (0.70 – 0.77, 98%) | Gacche 2010 (7-Hydroxyflavone) |
|  |  | 0.74 (0.71 – 0.77, 97%) | Gacche 2010 (Flavone) |
|  |  | 0.74 (0.70 – 0.77, 98%) | Gacche 2011 (5,7-Dihydroxyflavone) |
|  |  | 0.74 (0.71 – 0.77, 97%) | Gacche 2011 (Apigenin) |
|  |  | 0.74 (0.70 – 0.77, 98%) | Gacche 2011 (Luteolin) |
|  |  | 0.73 (0.69 – 0.76, 97%) | Gacche 2015 (4'-Methoxyflavone) |
|  |  | 0.73 (0.69 – 0.77, 98%) | Gacche 2015 (Diosmin) |
|  |  | 0.73 (0.70 – 0.77, 98%) | Pratheeshkumar 2012 (Luteolin) |
|  |  | 0.73 (0.70 – 0.77, 98%) | Zhu 2016 (Baicalein) |
|  |  | 0.73 (0.70 – 0.77, 98%) | Zhu 2016 (Baicalin) |
| Flavonols | 0.74 (0.69 – 0.79, 99%) | 0.75 (0.70 – 0.80, 99%) | Gacche 2010 (3-Hydroxyflavone) |
|  |  | 0.72 (0.68 – 0.77, 99%) | Gacche 2011 (3,6-Dihydroxyflavone) |
|  |  | 0.73 (0.68 – 0.78, 99%) | Gacche 2011 (3,7-Dihydroxyflavone) |
|  |  | 0.74 (0.70 – 0.79, 99%) | Gacche 2011 (Fisetin) |
|  |  | 0.75 (0.70 – 0.79, 99%) | Gacche 2011 (Kaempferol) |
|  |  | 0.75 (0.70 – 0.79, 99%) | Gacche 2011 (Quercetin) |
|  |  | 0.74 (0.69 – 0.79, 99%) | Gacche 2011 (Rutin) |
|  |  | 0.73 (0.68 – 0.78, 99%) | Gacche 2015 (3-Hydroxy-7-methoxy flavone) |
|  |  | 0.74 (0.69 – 0.79, 99%) | Gacche 2015 (Myricetin) |
| Flavanols | 0.74 (0.55 – 0.99, 100%) | 0.86 (0.84 – 0.88, NA) | Gacche 2015 (Silibinin) |
|  |  | 0.64 (0.63 – 0.65, NA) | Gacche 2015 (Taxifolin) |
| Flavanones | 0.84 (0.80 – 0.89, 98%) | 0.84 (0.79 – 0.90, 98%) | Gacche 2015 (2'-Hydroxyflavanone) |
|  |  | 0.84 (0.79 – 0.89, 98%) | Gacche 2015 (4'-Hydroxyflavanone) |
|  |  | 0.84 (0.79 – 0.89, 98%) | Gacche 2015 (7-Hydroxyflavanone) |
|  |  | 0.84 (0.79 – 0.89, 98%) | Gacche 2015 (Hesperidin) |
|  |  | 0.84 (0.79 – 0.90, 98%) | Gacche 2015 (Hesperitin) |
|  |  | 0.86 (0.81 – 0.91, 98%) | Gacche 2015 (Naringenin) |
|  |  | 0.86 (0.83 – 0.90, 96%) | Gacche 2015 (Naringin) |
| Anthocyanidines | 1.07 (0.86 – 1.33, 81%) | 1.14 (0.87 – 1.48, 84%) | Viegas 2019 (Cyanidin) |
|  |  | 0.99 (0.81 – 1.22, 70%) | Viegas 2019 (Cyanidin-3-*O*-glucoside) |
|  |  | 1.02 (0.79 – 1.31, 85%) | Viegas 2019 (Delphinidin) |
|  |  | 1.14 (0.89 – 1.47, 79%) | Viegas 2019 (Delphinidin-3-*O*-glucoside) |

NA: Not applicable; results highlighted in red showed >10% change in either re-pooled summary estimates or *I^2.^*

**Table 3.** Medium concentration range (40-50 µM) sensitivity analysis.

| Subgroup | Before Overall summary (95%CI X - Y, *I^2^*) | After Overall summary (95%CI X - Y, *I^2^*) | Left-out-study |
| --- | --- | --- | --- |
| Isoflavones | 0.46 (0.44 – 0.49, 92%) | 0.47 (0.41 – 0.55, 88%)­­ | Gacche 2011 (Genistein) |
|  |  | 0.48 (0.44 – 0.53, 68%) | Gacche 2015 (Biochanin-A) |
|  |  | 0.45 (0.43 – 0.48, 94%) | Gacche 2015 (Formononetin) |
| Flavonols | 0.50 (0.46 – 0.56, 100%) | 0.53 (0.48 – 0.58, 100%) | Gacche 2010 (3-Hydroxyflavone) |
|  |  | 0.49 (0.44 – 0.54, 100%) | Gacche 2011 (3,6-Dihydroxyflavone) |
|  |  | 0.50 (0.45 – 0.56, 100%) | Gacche 2011 (3,7-Dihydroxyflavone) |
|  |  | 0.50 (0.45 – 0.56, 100%) | Gacche 2011 (Fisetin) |
|  |  | 0.51 (0.45 – 0.57, 100%) | Gacche 2011 (Kaempferol) |
|  |  | 0.50 (0.45 – 0.57, 100%) | Gacche 2011 (Quercetin) |
|  |  | 0.50 (0.45 – 0.56, 100%) | Gacche 2011 (Rutin) |
|  |  | 0.50 (0.44 – 0.56, 100%) | Gacche 2015 (3-Hydroxy-7-methoxy flavone) |
|  |  | 0.52 (0.48 – 0.57, 100%) | Gacche 2015 (Myricetin) |
| Flavanols | 0.53 (0.27 – 1.02, 100%) | 0.74 (0.73 – 0.76, NA) | Gacche 2015 (Silibinin) |
|  |  | 0.38 (0.37 – 0.39, NA) | Gacche 2015 (Taxifolin) |
| Flavones | 0.55 (0.45 – 0.67, 100%) | 0.59 (0.51 – 0.68, 100%) | Bhat 2013 (Acacetin) |
|  |  | 0.52 (0.44 – 0.61, 100%) | Gacche 2010 (5-Hydroxyflavone) |
|  |  | 0.54 (0.43 – 0.67, 100%) | Gacche 2010 (6-Hydroxyflavone) |
|  |  | 0.54 (0.44 – 0.66, 100%) | Gacche 2010 (7-Hydroxyflavone) |
|  |  | 0.56 (0.45 – 0.69, 100%) | Gacche 2010 (Flavone) |
|  |  | 0.55 (0.44 – 0.69, 100%) | Gacche 2011 (5,7-Dihydroxyflavone) |
|  |  | 0.55 (0.44 – 0.69, 100%) | Gacche 2011 (Apigenin) |
|  |  | 0.55 (0.44 – 0.69, 100%) | Gacche 2011 (Luteolin) |
|  |  | 0.54 (0.44 – 0.67, 100%) | Gacche 2015 (4'-Methoxyflavone) |
|  |  | 0.56 (0.45 – 0.69, 100%) | Gacche 2015 (Diosmin) |
|  |  | 0.55 (0.45 – 0.68, 100%) | Pratheeshkumar 2012 (Luteolin) |
|  |  | 0.55 (0.45 – 0.67, 100%) | Zhu 2016 (Baicalein) |
| Flavanones | 0.74 (0.68 – 0.80, 99%) | 0.73 (0.67 – 0.80, 100%) | Gacche 2015 (2'-Hydroxyflavanone) |
|  |  | 0.74 (0.67 – 0.81, 99%) | Gacche 2015 (4'-Hydroxyflavanone) |
|  |  | 0.73 (0.66 – 0.81, 100%) | Gacche 2015 (7-Hydroxyflavanone) |
|  |  | 0.72 (0.66 – 0.79, 99%) | Gacche 2015 (Hesperidin) |
|  |  | 0.72 (0.66 – 0.80, 99%) | Gacche 2015 (Hesperitin) |
|  |  | 0.75 (0.69 – 0.82, 99%) | Gacche 2015 (Naringenin) |
|  |  | 0.76 (0.71 – 0.81, 99%) | Gacche 2015 (Naringin) |
| Anthocyanidines | 1.00 (0.94 – 1.07, 0%) | 1.00 (0.94 – 1.07, 0%) | Viegas 2019 (Cyanidin) |
|  |  | 1.00 (0.92 – 1.09, 0%) | Viegas 2019 (Cyanidin-3-*O*-glucoside) |
|  |  | 0.99 (0.93 – 1.06, 0%) | Viegas 2019 (Delphinidin) |
|  |  | 1.01 (0.93 – 1.10, 0%) | Viegas 2019 (Delphinidin-3-*O*-glucoside) |

NA: Not applicable; results highlighted in red showed >10% change in either re-pooled summary estimates or *I^2.^*

**Table 4.** High concentration (100 µM) sensitivity analysis.

| Subgroup | Before Overall summary (95%CI X - Y, *I^2^*) | After Overall summary (95%CI X - Y, *I^2^*) | Left-out-study |
| --- | --- | --- | --- |
| Isoflavones | 0.20 (0.14 - 0.29, 100%) | 0.25 (0.19 – 0.33, 100%)­­ | Gacche 2011 (Genistein) |
|  |  | 0.19 (0.09 – 0.42, 100%) | Gacche 2015 (Biochanin-A) |
|  |  | 0.17 (0.10 – 0.28, 100%) | Gacche 2015 (Formononetin) |
| Flavonols | 0.26 (0.19 – 0.35, 100%) | 0.26 (0.18 – 0.38, 100%) | Gacche 2010 (3-Hydroxyflavone) |
|  |  | 0.24 (0.18 – 0.32, 100%) | Gacche 2011 (3,6-Dihydroxyflavone) |
|  |  | 0.24 (0.18 – 0.34, 100%) | Gacche 2011 (3,7-Dihydroxyflavone) |
|  |  | 0.25 (0.18 – 0.36, 100%) | Gacche 2011 (Fisetin) |
|  |  | 0.28 (0.21 – 0.36, 100%) | Gacche 2011 (Kaempferol) |
|  |  | 0.27 (0.19 – 0.39, 100%) | Gacche 2011 (Quercetin) |
|  |  | 0.26 (0.18 – 0.37, 100%) | Gacche 2011 (Rutin) |
|  |  | 0.25 (0.18 – 0.35, 100%) | Gacche 2015 (3-Hydroxy-7-methoxy flavone) |
|  |  | 0.26 (0.18 – 0.36, 100%) | Gacche 2015 (Myricetin) |
| Flavanols | 0.28 (0.07 – 1.12, 100%) | 0.57 (0.56 – 0.58, NA) | Gacche 2015 (Silibinin) |
|  |  | 0.14 (0.14 – 0.14, NA) | Gacche 2015 (Taxifolin) |
| Flavones | 0.36 (0.29 – 0.44, 100%) | 0.35 (0.28 – 0.45, 100%) | Gacche 2010 (5-Hydroxyflavone) |
|  |  | 0.35 (0.28 – 0.44, 100%) | Gacche 2010 (6-Hydroxyflavone) |
|  |  | 0.34 (0.27 – 0.43, 100%) | Gacche 2010 (7-Hydroxyflavone) |
|  |  | 0.36 (0.28 – 0.46, 100%) | Gacche 2010 (Flavone) |
|  |  | 0.36 (0.28 – 0.45, 100%) | Gacche 2011 (5,7-Dihydroxyflavone) |
|  |  | 0.37 (0.29 – 0.47, 100%) | Gacche 2011 (Apigenin) |
|  |  | 0.39 (0.32 – 0.47, 100%) | Gacche 2011 (Luteolin) |
|  |  | 0.34 (0.27 – 0.42, 100%) | Gacche 2015 (4'-Methoxyflavone) |
|  |  | 0.37 (0.29 – 0.49, 100%) | Gacche 2015 (Diosmin) |
|  |  | 0.34 (0.27 – 0.43, 100%) | Zhu 2016 (Baicalin) |
| Flavanones | 0.58 (0.53 – 0.64, 99%) | 0.58 (0.52 – 0.66, 99%) | Gacche 2015 (2'-Hydroxyflavanone) |
|  |  | 0.58 (0.52 – 0.64, 99%) | Gacche 2015 (4'-Hydroxyflavanone) |
|  |  | 0.58 (0.51 – 0.65, 99%) | Gacche 2015 (7-Hydroxyflavanone) |
|  |  | 0.57 (0.51 – 0.63, 99%) | Gacche 2015 (Hesperidin) |
|  |  | 0.57 (0.51 – 0.64, 99%) | Gacche 2015 (Hesperitin) |
|  |  | 0.59 (0.53 – 0.66, 99%) | Gacche 2015 (Naringenin) |
|  |  | 0.60 (0.56 – 0.65, 99%) | Gacche 2015 (Naringin) |
| Anthocyanidines | 0.82 (0.74 – 0.90, 40%) | 0.83 (0.72 – 0.95, 60%) | Viegas 2019 (Cyanidin) |
|  |  | 0.81 (0.72 – 0.91, 54%) | Viegas 2019 (Cyanidin-3-*O*-glucoside) |
|  |  | 0.77 (0.71 – 0.84, 0%) | Viegas 2019 (Delphinidin) |
|  |  | 0.87 (0.78 – 0.96, 0%) | Viegas 2019 (Delphinidin-3-*O*-glucoside) |

NA: Not applicable; results highlighted in red showed >10% in either re-pooled summary estimates or *I^2.^*
